# Mendelian randomization analysis of female reproductive factors on osteoarthritis

**DOI:** 10.1097/MD.0000000000041362

**Published:** 2025-01-31

**Authors:** Liang Pang, Kai Wu, Pingping Su, Zhicheng Liao, Cunxian Lv

**Affiliations:** a Wenzhou TCM Hospital of Zhejiang Chinese Medical University, Wenzhou, China.

**Keywords:** causality, GWAS, Mendelian randomization, osteoarthritis, reproductive physiological phenomena

## Abstract

Epidemiology shows women have a higher incidence of osteoarthritis (OA) than men. However, there is not enough evidence to suggest a direct correlation between female reproductive factors and OA. Therefore, this study will employ Mendelian randomization (MR) analysis to investigate whether there is a causal relationship between the 2. This study used a 2-sample MR analysis with single nucleotide polymorphisms significantly associated with female reproductive factors as instrumental variables (IV). We used inverse variance weighted (IVW), MR-Egger regression, weighted median method to infer a causal relationship between female reproductive factors and OA, Cochran Q heterogeneity test by IVW and MR-Egger method, MR PRESSO method and IVW-radial method to detect outliers, MR_pleiotropy_test function and MR PRESSO method for multivariate validity test, and calculation of *F*-value was used to assess the presence of weak IVs. Finally, the stability of the findings was assessed using the leave-one-out method. Our research shows that there is no reliable causal relationship between an increase in Age at menarche (years) (AAM) and Age at menopause (years) (AM) and OA, that an increase in Age first had sexual intercourse (years) (AFS) is associated with a decreased risk of knee OA and/or hip OA and hand OA, that an increase in Age at first live birth (years) (AFB) is associated with a decreased risk of knee OA and/or hip OA and knee OA, and that an increase in Number of live births (NOB) is associated with an increased risk of knee OA and/or hip OA. This study provides genetic support for an increase in AFS as a reduced knee OA and/or hip OA and hand OA risk factor, an increase in AFB as a reduced knee OA and/or hip OA and knee OA risk factor, and an increase in NOB as an increased knee OA and/or hip OA risk factor. Further studies are needed to elucidate the potential mechanisms underlying the causal associations between AFS, AFB, and NOB and site-specific OA.

## 1. Introduction

Osteoarthritis (OA) is a degenerative condition characterized by cartilage degeneration, subchondral bone changes, and synovial inflammation. Clinical manifestations include slow-onset joint pain, stiffness, swelling, deformity, and limited mobility. As 1 of the common orthopedic diseases in clinical practice, osteoarthritis has a high incidence and disability rate worldwide.^[1]^ As the global population ages at an accelerated pace, the incidence of OA is increasing year by year, leading to a rising socioeconomic burden.^[2,3]^ For patients with severe hip and knee OA, undergoing hip or knee joint replacement surgery is often necessary to improve future quality of life. However, various postoperative complications significantly increase the associated risks.^[4–6]^ Therefore, early intervention for the disease becomes particularly important. However, the exact mechanisms underlying the onset of OA are not yet fully understood. By identifying high-risk factors that increase the risk of OA, we can promptly identify individuals at high risk for the development of OA. Early prevention measures for OA can then be implemented, helping to alleviate the burden imposed by this condition.

Epidemiological studies have shown a significant gender difference in the incidence of OA, with a much higher prevalence in females compared to males. According to statistics, in the population aged over 60, the probability of females developing OA is 18% higher than that of males. This trend persists in middle-aged populations, where females still have a higher likelihood of developing the condition.^[7,8]^ A wealth of research indicates a close relationship between female hormone levels and the occurrence of OA. Studies have found that estrogen has a potential protective effect on joint cartilage, and its deficiency can also impact other joint tissues in the process of OA, including the synovial lining, periarticular muscles, ligaments, and joint capsule.^[9,10]^ Simultaneously, numerous observational studies also suggest a potential causal relationship between female reproductive factors and the onset of OA. Some studies employ hormone replacement therapy to alleviate certain osteoarthritic symptoms.^[11]^ However, some scholars have found that the use of estrogen by women does not affect the development of OA.^[12]^ Due to the susceptibility of observational studies to various confounding factors and the focus of these scholars on individual factors of female estrogen, which undergo significant variations throughout a woman’s life, particularly during the reproductive period, thus observational studies do not provide a definitive and uniform conclusion regarding the causal association of female reproductive factors with OA.

Mendelian randomization (MR) is a method that utilizes genetic variations, typically single nucleotide polymorphisms (SNPs), as instrumental variables (IV) to study the causal effects between exposure factors and diseases.^[13]^ Genetic variants are randomly assigned to progeny during meiosis. This random assignment mitigates confounding factors and prevents reverse causality, enabling the MR method to mimic the effects of a randomized controlled trial.^[14]^ This distinct statistical advantage allows MR to yield more precise and reliable causal inferences. There was a previous MR study on this topic,^[15]^ but it had significant limitations. The OA data included in the study were not stratified by gender, and the proportion of females was relatively low (female proportion: 49.9% for knee OA and/or hip OA data; 54.3% for hip OA data; 47.6% for knee OA data). The reliability of the inferred conclusions is greatly compromised due to the lack of gender stratification, and the study did not comprehensively include all relevant female reproductive factors. Therefore, in this study, we chose a more comprehensive set of female reproductive factors as the exposure variable. The latest genetic consortium data on OA, which has been stratified by gender, was selected as the outcome variable. This study aims to explore the causal relationship between female reproductive factors and OA more comprehensively, the goal is to provide insights for the early prevention of OA in women.

## 2. Methods

### 2.1. Research design

**Figure 1.**
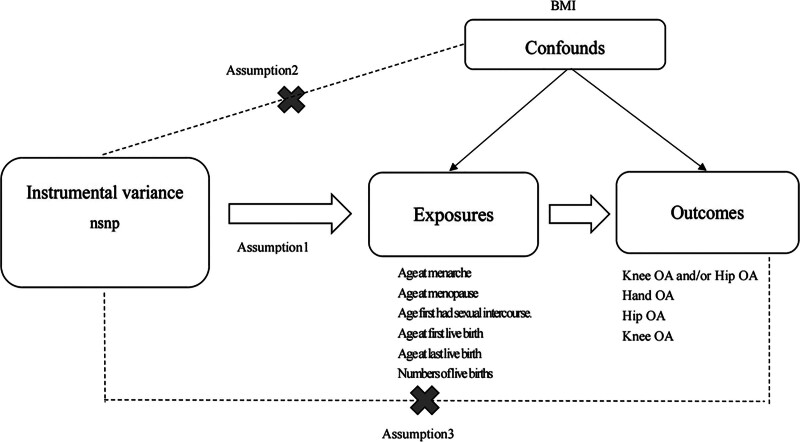
Basic assumptions for Mendelian randomization.

This study used a 2-sample MR analysis to select SNPs significantly associated with female reproductive factors as IVs. We separately investigated their causal relationships with knee OA and/or hip OA, hand OA, hip OA, and knee OA. The MR study design must satisfy 3 major assumptions: the relevance assumption – IVs are strongly correlated with the exposure factor; the independence assumption - IVs are unrelated to confounding factors. In this study, considering body mass index as a confounding factor influencing the risk of hand OA,^[16]^ knee OA,^[17]^ and hip OA^[18]^ to enhance the reliability of conclusions, we used phenoscanner website to eliminate confounding factors from the SNPs of the exposure factor before conducting MR analysis; the exclusivity assumption, which dictates that the genetic variation affects the outcome solely through the exposure factors, and not directly^[19]^ (Fig. 1).

### 2.2. Data sources

The GWAS database provides data on the entire human genome, offering the necessary conditions for studying causal relationships related to diseases. In this study, all data on female reproductive factors were sourced from the OpenGWAS database. The age at menarche (years) (AAM) was derived from a large-scale GWAS meta-analysis, encompassing 252,514 individuals, which identified 389 independent signals (*P* < 5E−08) associated with the AAM.^[20]^ The age at menopause (years) (AM), the age first had sexual intercourse (years) (AFS), the age at first live birth (years) (AFB), the age at last live birth (years) (ALB), and the number of live births (NOB) were all obtained from the UK Biobank. The UK Biobank is a large biomedical database and research resource containing in-depth, de-identified genetic and health information from 500,000 UK participants. AAM included 143,819 individuals, the AFS included 170,498 individuals, the AFB included 170,248 individuals, the ALB included 250,782 individuals, and the NOB included 406,457 individuals. The OA database originated from the latest large-scale GWAS meta-analysis by the Osteoarthritis Genetics Consortium. This meta-analysis involved 826,690 individuals (177,517 with osteoarthritis) and conducted a genome-wide association study, identifying 100 independent risk variants associated with 11 osteoarthritis phenotypes, including 52 variants not previously linked to the disease, The European population accounts for more than 78 %, OA was defined by either: self-reported osteoarthritis, clinically diagnosed, ICD10 codes or radiographic depending on the data available in the cohort. Controls were osteoarthritis-free or population-based with or without ICD code exclusions^[21]^ (Table S1, Supplemental Digital Content, http://links.lww.com/MD/O314).

### 2.3. Genetic variants selection criteria

The inclusion and exclusion criteria for IVs in this study are as follows: inclusion criteria: relevance criterion: SNPs significantly associated with the exposure variable (female reproductive factors) were selected. The statistical threshold for significance was set at *P* < 5E−08 to ensure a strong correlation with the exposure variable.^[22]^ Independence criterion: to minimize the impact of linkage disequilibrium (LD), SNPs were using the following criteria: LD measure *r*^2^ < 0.001 between any pair of SNPs. Physical distance between SNPs > 10,000 base pairs.^[23]^ Effect Allele Harmonization: Harmonization of SNPs was performed to ensure that all effect alleles corresponded to the same reference allele for both the exposure and outcome variables. Instrument strength: SNPs with an *F*-statistic >10 (calculated as *F* = β^2^/SE^2^) were included to avoid weak instrument bias.^[24]^ Exclusion criteria: weak instruments: SNPs with an *F*-statistic ≤ 10 were excluded to prevent bias from weak instruments. LD: SNPs with *r*^2^ ≥ 0.001 or within 10,000 base pairs of another SNP were excluded to eliminate LD-related redundancy. Genotype issues: SNPs with inconsistent genotype coding, mismatched rsIDs, or missing data were discarded without searching for proxy SNPs. (d) Potential Confounders: SNPs associated with confounding factors, such as BMI, were identified using the Phenoscanner database and excluded. When ALB was used as an exposure factor, according to the criteria for SNP selection in this study, no SNP was found that was sufficient for MR analyses after excluding 1 rs6446187 SNP for the confounder BMI.

### 2.4. Mendelian randomization analysis

After the data processing, all statistical analyses were conducted using the R software (version 4.3.2; R Foundation for Statistical Computing, Vienna, Austria).^[25]^ The MR analysis was performed with the TwoSampleMR package (version 0.6.8; developed by the MRC Integrative Epidemiology Unit, University of Bristol, UK), a specialized tool for analyzing GWAS summary data. Data on confounding factors were obtained from the Phenoscanner database (Phenoscanner V2; developed by the Department of Public Health and Primary Care, University of Cambridge, UK; accessible at http://www.phenoscanner.medschl.cam.ac.uk). Plots and figures, including forest plots and funnel plots, were generated using the ggplot2 package (version 3.4.0; maintained by the R Core Team, USA). All analysis and figure generation were performed on a Linux-based computing server running Ubuntu 22.04 (Canonical Ltd., London, United Kingdom), ensuring computational efficiency and reproducibility. In this study, the inverse variance weighting (IVW) method was employed as the primary analytical approach for MR analysis. The IVW method can deliver the most accurate estimates of causal association when all selected SNPs function as valid IVs. Additionally, the MR-Egger regression method and the weighted median method were utilized as supplementary analytical approaches. The weighted median method is effective in providing valid causal estimates when over half of the SNPs are considered valid IVs.^[26]^ In cases where all SNPs are deemed invalid, the MR-Egger regression method still offers reliable causal estimates.^[27]^ All raw data, including detailed SNP-level statistics and analytical results, are available in Supplemental Digital Content 1 and 2, Supplemental Digital Content, http://links.lww.com/MD/O316.

### 2.5. Sensitivity analysis

To verify the robustness of our findings, heterogeneity was assessed using both the IVW and MR-Egger regression methods. The magnitude of heterogeneity was quantified by calculating Cochran *Q* statistic, with a *P*-value <.05 indicating the presence of significant heterogeneity.^[28]^ Horizontal pleiotropy was evaluated by computing the intercept of the MR-Egger regression; a *P*-value <.05 for this intercept suggested the presence of horizontal pleiotropy, rendering the MR results potentially unreliable.^[27]^ The MR PRESSO global test and the IVW_radical method were utilized to detect outliers.^[29,30]^ An MR PRESSO global test *P*-value <.05 signified the presence of outliers, necessitating their exclusion and a subsequent reanalysis of the MR data. The absence of outliers on the radial plot confirmed their effective removal. Additionally, the leave-one-out method was employed to determine if the MR results were disproportionately influenced by any single SNP.

Given that our study involved multiple groups, we applied Bonferroni correction to adjust the significance threshold for correlation. A *P*-value of <.0025 for the IVW method (0.05/5*4) was required to establish a correlation. In summary, results demonstrating stable correlation should meet the following criteria: A *P*-value of <.0025 for the IVW method. A consistent direction of causal estimation across the 3 MR analysis methods, namely MR-Egger regression, the weighted median method, and the IVW method. Results from multiplicity and sensitivity tests indicate that the findings are not driven by single SNPs and do not exhibit multiplicative effects.

## 3. Results

### 3.1. Causal effects of AAM and AM on OA and its subtypes

**Figure 2.**
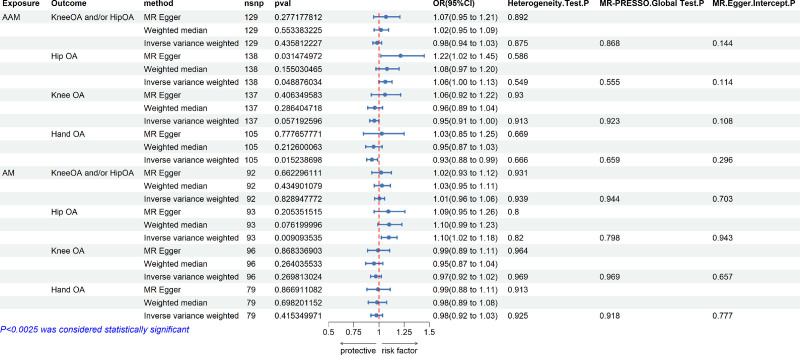
Causal effects of AAM and AM on OA and its subtypes. AAM = age at menarche, AM = age at menopause, OA = osteoarthritis.

Confounding BMI culling of AAM and AM before MR analysis, after conducting the analyses with AAM and AM as an exposure factor and osteoarthritis and its subtypes as outcome factors, the final data on valid SNPs for AAM, AM and OA were obtained. After excluding SNPs associated with BMI, the MR analysis did not identify any significant causal associations between AAM and OA or its subtypes, including knee OA and/or hip OA, hip OA, knee OA, and hand OA (Fig. 2). Similarly, no significant causal associations were observed between AM and any OA subtype. The 3 main methods of MR analysis – IVW, weighted median, and MR-Egger regression – showed direction of association (increase or decrease risk) (Fig. S1A–D and E–H, Supplemental Digital Content, http://links.lww.com/MD/O315). Radial plots show that there are no outliers in the data (Fig. S2A–D and E–H, Supplemental Digital Content, http://links.lww.com/MD/O315). The test for heterogeneity between AAM, AM and the 4 OA phenotypes showed that there was no heterogeneity between them (test: *P* > .05). The correlation funnel plots (Fig. S3A–D and E–H, Supplemental Digital Content, http://links.lww.com/MD/O315). The sensitivity test results for AAM, AM with the 4 OA phenotypes demonstrated that the results of the MR analysis were highly robust and not easily influenced by an individual SNP. The associated leave-one-out forest plots (Fig. S4A–D and E–H, Supplemental Digital Content, http://links.lww.com/MD/O315). Pleiotropy analysis by MR_pleiotropy_test function and MR-PRESSO method showed that the *P*-value of both pleiotropy_test and MR-PRESSO was >.05. Therefore, we conclude that there is no reliable causal association between AAM, AM, and OA.

### 3.2. Causal effects of AFS, AFB, and NOB on OA and its subtypes

**Figure 3.**
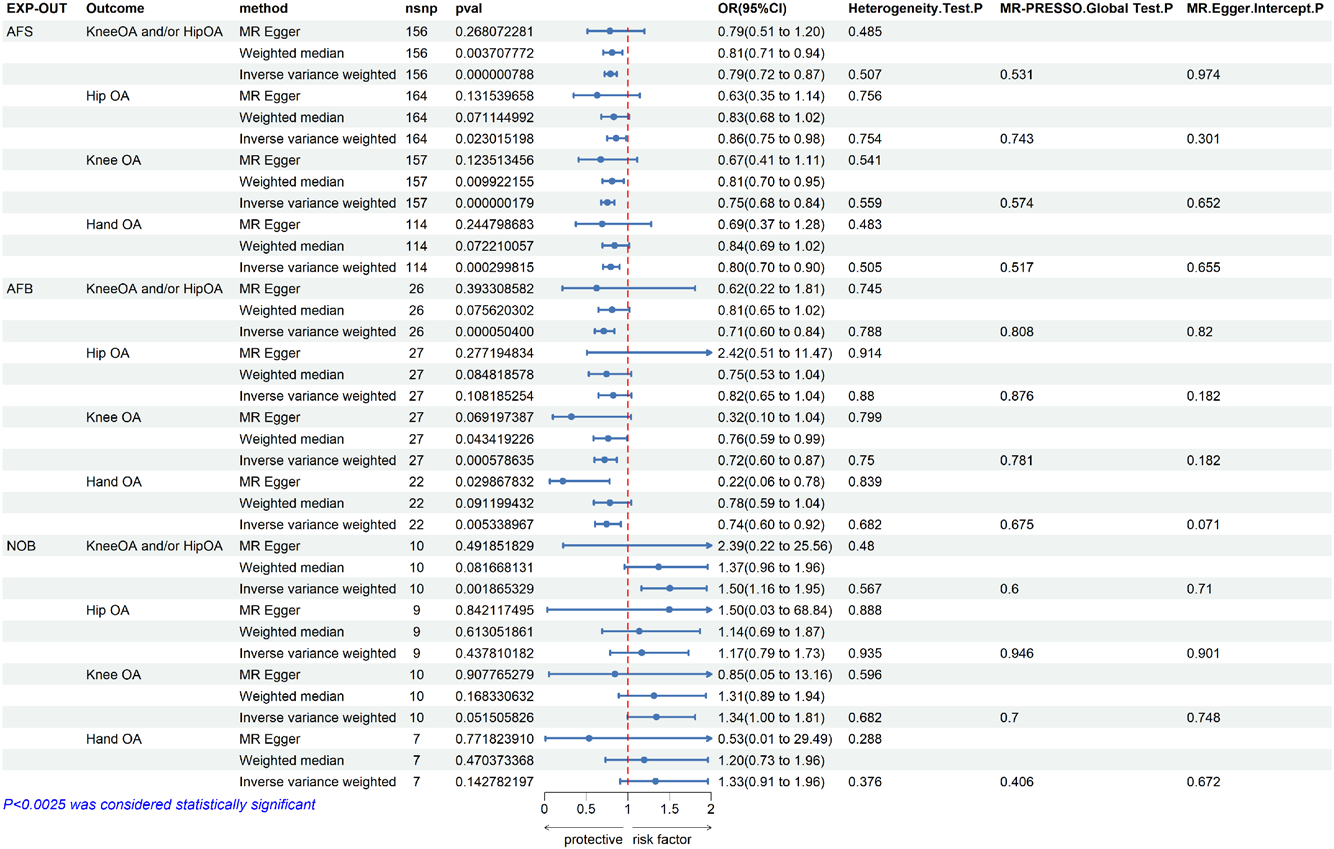
Causal effects of AFS, AFB, and NOB on OA and its subtypes. AFB = age at first live birth, AFS = age first had sexual intercourse, NOB = number of live births, OA = osteoarthritis.

Confounding BMI culling of AFS before MR analysis, after conducting the analyses with AFS as an exposure factor and osteoarthritis and its subtypes as outcome factors, the final data on valid SNPs for AFS and OA were obtained (156 valid SNPs related to knee OA and/or hip OA, 164 valid SNPs related to hip OA, and 157 valid SNPs related to knee OA, 114 valid SNPs related to hand OA). The results of the MR analysis for AFS and OA were as follows:

Knee OA and/or hip OA IVW: *P* < .0025, OR = 0.79, 95% CI: 0.72 to 0.87. Hand OA IVW: *P* < .0025, OR = 0.80, 95% CI: 0.70 to 0.90 (Fig. 3). The 3 main methods of MR analysis – IVW, weighted median, and MR-Egger regression – showed the same direction (decreasing risk) in 4 OA phenotypes (Fig. A1I–L, Supplemental Digital Content, http://links.lww.com/MD/O315). Radial plots show no outliers in the data (Fig. S2I–L, Supplemental Digital Content, http://links.lww.com/MD/O315). The test for heterogeneity between AFS and the 4 OA phenotypes showed that there was no heterogeneity between them (test: *P* > .05). The correlation funnel plots (Fig. S3I–L, Supplemental Digital Content, http://links.lww.com/MD/O315). The sensitivity test results for AFS with the 4 OA phenotypes demonstrated that the results of the MR analysis were highly robust and not easily influenced by an individual SNP. The associated leave-one-out forest plots (Fig. S4I–L, Supplemental Digital Content, http://links.lww.com/MD/O315). Pleiotropy analysis by MR_pleiotropy_test function and MR-PRESSO method showed that the *P*-value of both pleiotropy_test and MR-PRESSO was >.05. Therefore, we conclude that increased AFS may be a factor in reducing the risk of knee OA and/or hip OA and hand OA.

Confounding BMI culling of AFB prior to MR analysis, after conducting the analyses with AFB as an exposure factor and osteoarthritis and its subtypes as outcome factors, the final data on valid SNPs for AFB and OA were obtained (26 valid SNPs related to knee OA and/or hip OA, 27 valid SNPs related to hip OA, and 27 valid SNPs related to knee OA, 22 valid SNPs related to hand OA). The results of the MR analysis for AFB and OA were as follows:

Knee OA and/or hip OA IVW: *P* < .0025, OR = 0.71, 95% CI: 0.60 to 0.84. Knee OA IVW: *P* < .0025, OR = 0.72, 95% CI: 0.60 to 0.87 (Fig. 3). The 3 main methods of MR analysis – IVW, weighted median, and MR-Egger regression – showed the same direction (decreasing risk) in knee OA and/or hip OA, knee OA, and hand OA phenotypes (Fig. S1M–P, Supplemental Digital Content, http://links.lww.com/MD/O315). Radial plots show no outliers in the data (Fig. S2M–P, Supplemental Digital Content, http://links.lww.com/MD/O315). The test for heterogeneity between AFB and the 4 OA phenotypes showed that there was no heterogeneity between them (test: *P* > .05). The correlation funnel plots (Fig. S3M–P, Supplemental Digital Content, http://links.lww.com/MD/O315). The sensitivity test results for AFB with the 4 OA phenotypes demonstrated that the results of the MR analysis were highly robust and not easily influenced by an individual SNP. The associated leave-one-out forest plots (Fig. S4M–P, Supplemental Digital Content, http://links.lww.com/MD/O315). Pleiotropy analysis by MR_pleiotropy_test function and MR-PRESSO method showed that the *P*-value of both pleiotropy_test and MR-PRESSO was >.05. Therefore, we conclude that increased AFB may be a factor in reducing the risk of knee OA and/or hip OA and knee OA.

Confounding BMI culling of NOB before MR analysis culled out rs9862795 a SNP, after conducting the analyses with NOB as an exposure factor and osteoarthritis and its subtypes as outcome factors, the final data on valid SNPs for NOB and OA were obtained (10 valid SNPs related to knee OA and/or hip OA, 9 valid SNPs related to hip OA, and 10 valid SNPs related to knee OA, 7 valid SNPs related to hand OA). The results of the MR analysis for NOB and OA were as follows:

Knee OA and/or hip OA IVW: *P* < .0025, OR = 1.50, 95% CI: 1.16 to 1.95 (Fig. 3). The 3 main methods of MR analysis – IVW, weighted median, and MR-Egger regression – showed the same direction (increasing risk) in knee OA and/or hip OA phenotypes (Fig. S1Q–T, Supplemental Digital Content, http://links.lww.com/MD/O315). Radial plots show no outliers in the data (Fig. S2Q–T, Supplemental Digital Content, http://links.lww.com/MD/O315). The test for heterogeneity between NOB and the 4 OA phenotypes showed that there was no heterogeneity between them (test: *P* > .05). The correlation funnel plots (Fig. S3Q–T, Supplemental Digital Content, http://links.lww.com/MD/O315). The sensitivity test results for NOB with the 4 OA phenotypes demonstrated that the results of the MR analysis were highly robust and not easily influenced by an individual SNP. The associated leave-one-out forest plots (Fig. S4Q–T, Supplemental Digital Content, http://links.lww.com/MD/O315). Pleiotropy analysis by MR_pleiotropy_test function and MR-PRESSO method showed that the *P*-value of both pleiotropy_test and MR-PRESSO was >.05. Therefore, we conclude that increased NOB may be a factor in increasing the risk of knee OA and/or hip OA.

## 4. Discussion

In this Mendelian randomized study, we focused on the causal relationship between female reproductive factors (AAM, AM, AFS, AFB, ALB, NOB) and OA risk. According to the results of MR, we conclude that there is no reliable causal relationship between an increase in AAM and AM and OA, that an increase in AFS is associated with a decreased risk of knee OA and/or hip OA and hand OA, that an increase in AFB is associated with a decreased risk of knee OA and/or hip OA and knee OA, and that an increase in NOB is associated with an increased risk of knee OA and/or hip OA (Table S2, Supplemental Digital Content, http://links.lww.com/MD/O314). Since the confounder BMI was removed from the data by us via the phenoscanner website before the data was formally MR, we consider the findings to be reliable.

It is an academically recognized fact that the incidence of OA is significantly higher in the female population than in males.^[31–34]^ In a meta-analysis of the incidence of OA, researchers found a higher incidence of OA in women during menopause.^[35]^ When a woman enters menopause, her body undergoes significant changes, typically characterized by the cessation of menstruation and a rapid decline in the levels of the associated sex hormones,^[36]^ In addition, some women experience anxiety, depression, and menopausal obesity.^[37–39]^ Numerous studies have shown that decreased levels of estrogen and progesterone in women contribute to the high incidence of OA in women during menopause.^[40–43]^ In addition, receptors for estrogen and progesterone have been found on many bone and cartilage tissues,^[44]^ Estrogen reduces the body’s inflammatory response by binding to these receptors,^[45]^ Progesterone, on the other hand, may maintain cartilage volume by inhibiting matrix metalloproteinase production.^[46]^ In this way, female sex hormones play a fairly important role in the development of OA. In this study, we selected the exposure factors are AAM and AM, women into these 2 special period of time when the body sex hormones will occur significant changes, women into puberty will have the first menstruation, the body estrogen levels greatly increased.^[47]^ And when a woman enters menopause, the level of estrogen in her body decreases rapidly.^[48]^ According to the theory that estrogen affects OA, women with earlier AAM have a lower risk of OA, and women with earlier AM have a higher risk of OA. However, there are many contradictory observational studies available on the relationship between reproductive factors and OA in women; for example, A study by Leung et al concluded that higher number of births, earlier age at menopause and use of oral contraceptives were associated with increased severe knee OA in women.^[49]^ Cooley HM et al argue that number of births, increasing age at menopause and years of menstruation were associated with both symptomatic hand OA and a more severe DIP score.^[50]^ Jørgensen et al suggest that number of births is a factor that increases the risk of knee OA.^[51]^ Several of these studies support the association of female reproductive factors with OA. However, the study by Meng et al concluded that age at menarche, number of births and menopausal status were not factors influencing OA.^[52]^ The study by Wang et al concluded that reproductive factors were not clinically significantly associated with OA after controlling for confounding factors.^[53]^ Dennison et al also concluded that there was no association between the risk of hip OA and oral contraceptive use, number of births, or hysterectomy.^[54]^ Combined with the results of this study, we believe that the reasons for the multiple contradictions in observational studies may be different female individuals have different estrogen levels. Bone and cartilage tolerance thresholds to estrogen levels vary in different individuals. The effect of obesity on OA during adolescence and menopause in women. This is supported by the study of Hussain et al, who argue that it appears difficult to determine the direct effect of hormones on OA with the available data, and that further research should consider the mediating role of body weight and inflammation, changes in body weight over the life course, circulating levels of all endogenous hormones, and circulating levels of hormones following hormone supplementation in this complex relationship.^[55]^ Currently, most scholars believe that the significantly higher incidence of OA in women than in men is influenced solely by female sex hormones, ignoring the differences in certain reproductive behaviors between women and men.

This study has several strengths. The sample size of exposure and outcome data selected for this study is large, and the estimated results are close to the reality. Regarding female reproductive factors we selected 5 phenotypes, AAM, AM, AFS, AFB, NOB, which basically covered female reproductive traits. The effect of the confounding factor BMI was eliminated before the formal MR analysis, making the results more credible. The data source of OA phenotypes and their subtypes selected for this study was with the latest Osteoarthritis Genetics Consortium. Compared with other databases, the data we selected had been stratified by gender, which made it significantly more credible in analyzing the results. However, we still need more sample size data to further validate the results of the study.

In addition to female reproductive factors, biomechanical factors play a significant role in OA development and progression. Altered foot posture and dynamic plantar forces, for instance, may increase joint loading and degeneration, particularly in weight-bearing joints like the knee. Al-Bayati et al^[56]^ found that supinated foot posture was associated with narrowed medial joint space width and increased functional impairment in medial knee OA, while pronated foot posture altered the anatomical axis angle, potentially affecting joint alignment. Paterson et al^[57]^ highlighted those dynamic plantar forces, such as a higher medial-lateral force ratio and arch index, were weakly associated with increased knee pain, suggesting their relevance to OA symptoms. These biomechanical changes may interact with hormonal fluctuations during menopause, which affect ligament laxity and joint alignment. For example, pregnancy-related weight gain and postmenopausal estrogen deficiency can alter gait mechanics, further increasing joint stress. Although this study did not directly explore these interactions, incorporating biomechanical assessments into future research could provide a more comprehensive understanding of OA risk in women.

In conclusion, our study demonstrated the causal association of different reproductive factors on OA and its subtypes in females at the genetic level, which provides ideas for early prevention of OA in females, for example, females should pay attention to weight management during puberty and menopause, and avoidance of excessively high BMI can reduce the risk of OA. Late marriage and childbearing at an appropriate age for women can reduce the risk of OA. Avoiding too many births can also reduce the risk of OA. Women should pay attention to foot health and gait mechanics, especially during menopause or periods of weight gain. The use of foot orthotics or adjustments in gait mechanics to reduce the load on the knee and hip joints may help lower the risk of developing OA. Further research is needed in the future to elaborate on the potential mechanisms underlying the causal relationship between AFS, AFB, NOB, and site-specific OA.

## Author contributions

**Conceptualization:** Liang Pang.

**Data curation:** Liang Pang.

**Formal analysis:** Liang Pang, Zhicheng Liao.

**Investigation:** Kai Wu.

**Supervision:** Pingping Su, Zhicheng Liao.

**Validation:** Pingping Su, Zhicheng Liao.

**Visualization:** Kai Wu, Cunxian Lv.

**Writing – original draft:** Liang Pang.

**Writing – review & editing:** Cunxian Lv.

## Supplementary Material


