# Supplementary figures and images for: Mendelian randomization analysis of female reproductive factors on osteoarthritis
Source: Medicine (Baltimore). 2025 Jan 31;104(5):e41362. doi: 10.1097/MD.0000000000041362 (PMC11789898; doi:10.1097/MD.0000000000041362)

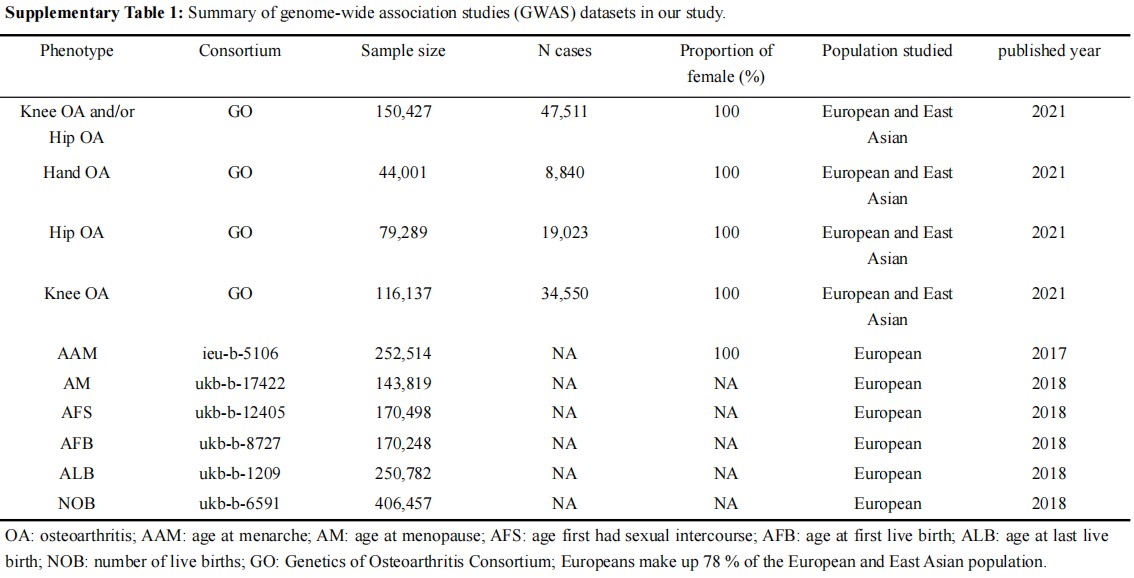


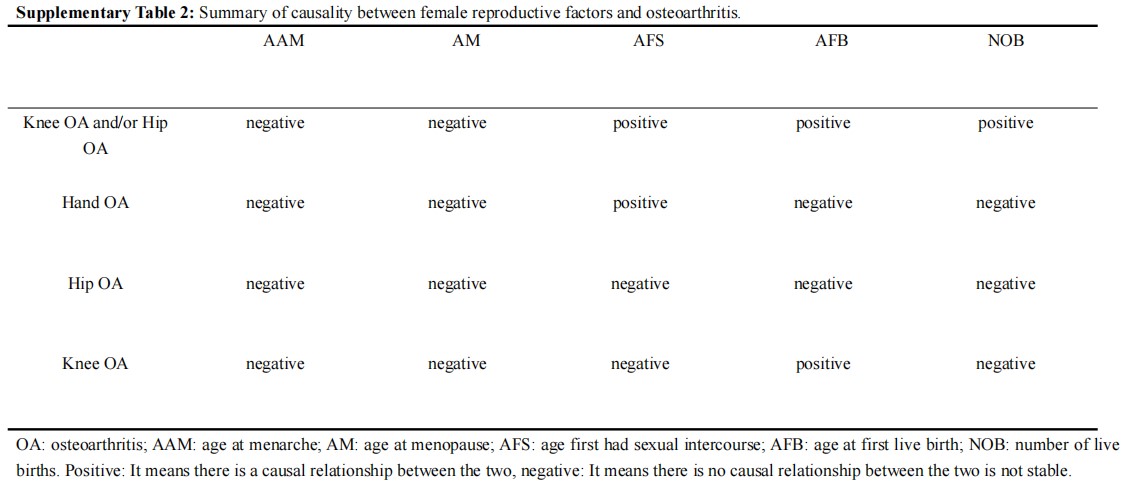

Supplement: Supplementary file 1 [file medi-104-e41362-s001.docx]
