# Supplementary material for: Mendelian randomization analysis of female reproductive factors on osteoarthritis
Source: Medicine (Baltimore). 2025 Jan 31;104(5):e41362. doi: 10.1097/MD.0000000000041362 (PMC11789898; doi:10.1097/MD.0000000000041362)

Supplementary figure 1 a

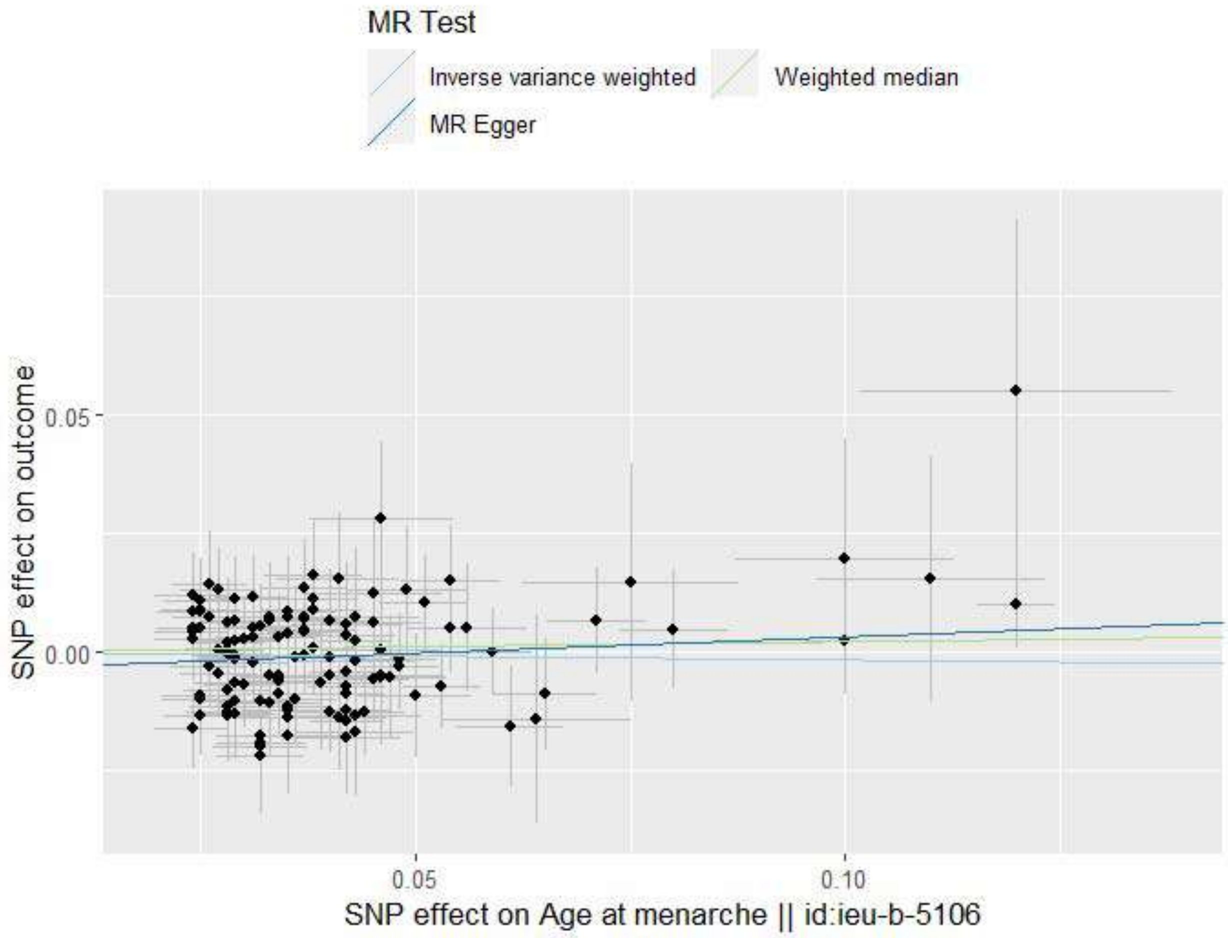

Supplementary figure 1 b

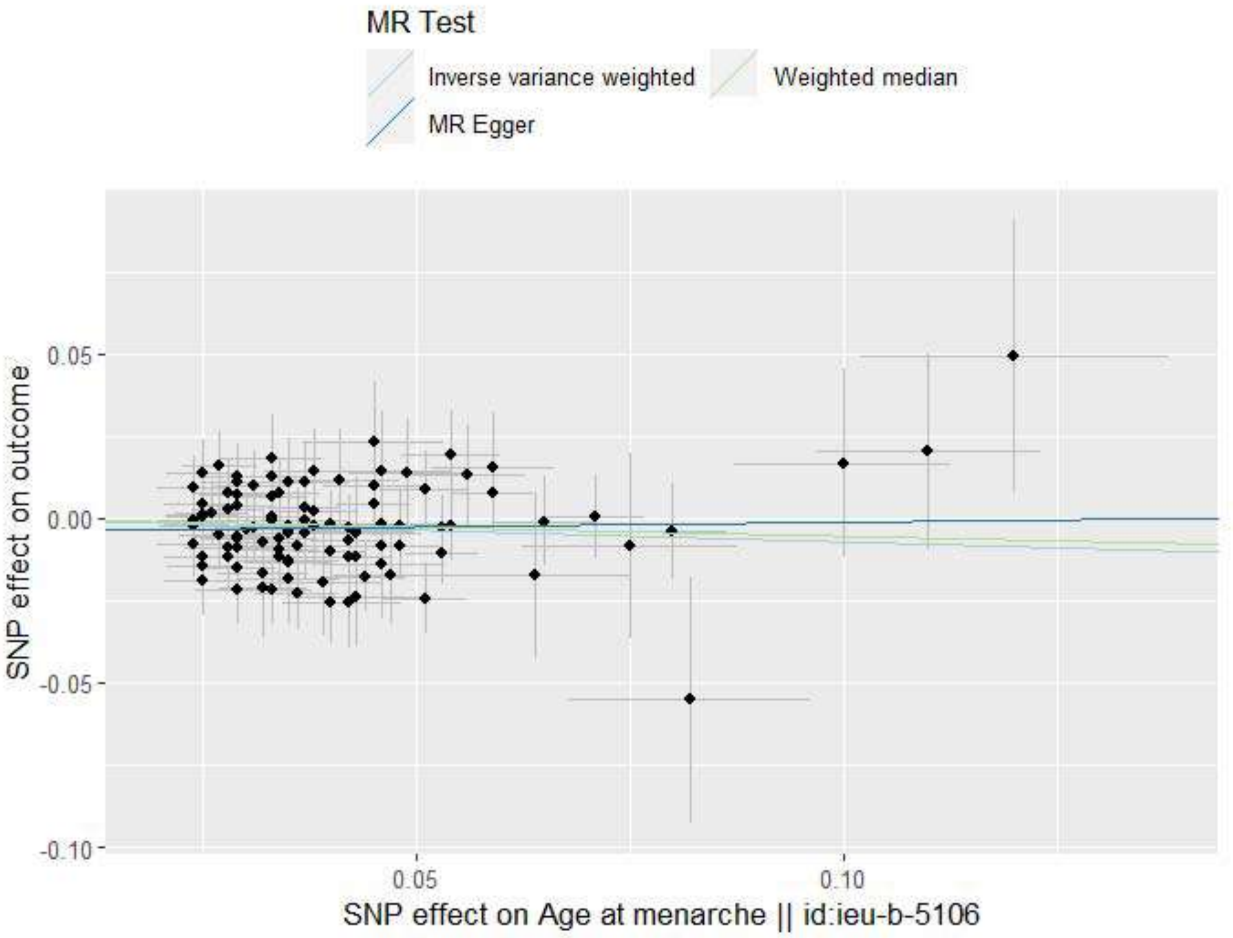

Supplementary figure 1 c

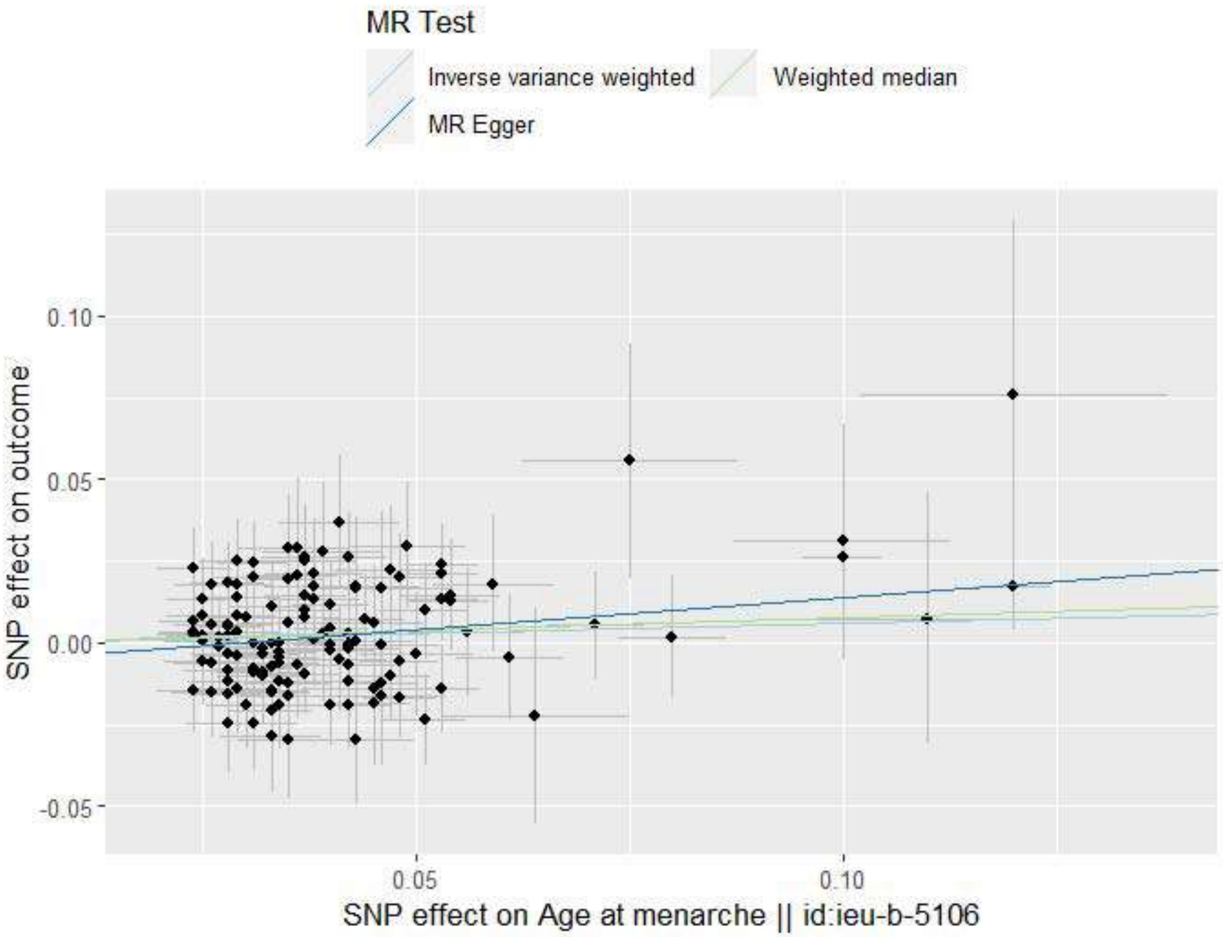

Supplementary figure 1 d

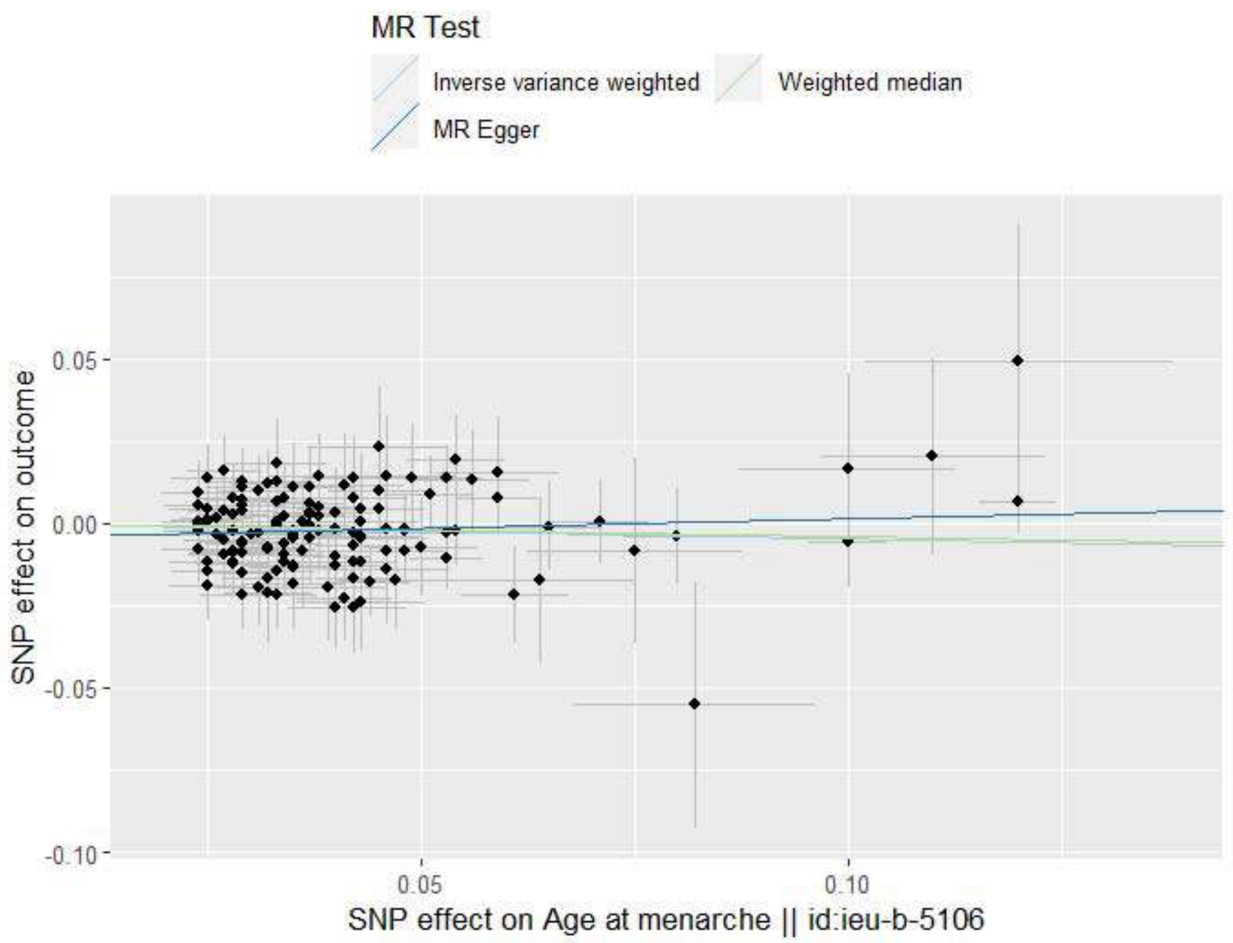

Supplementary figure 1 e

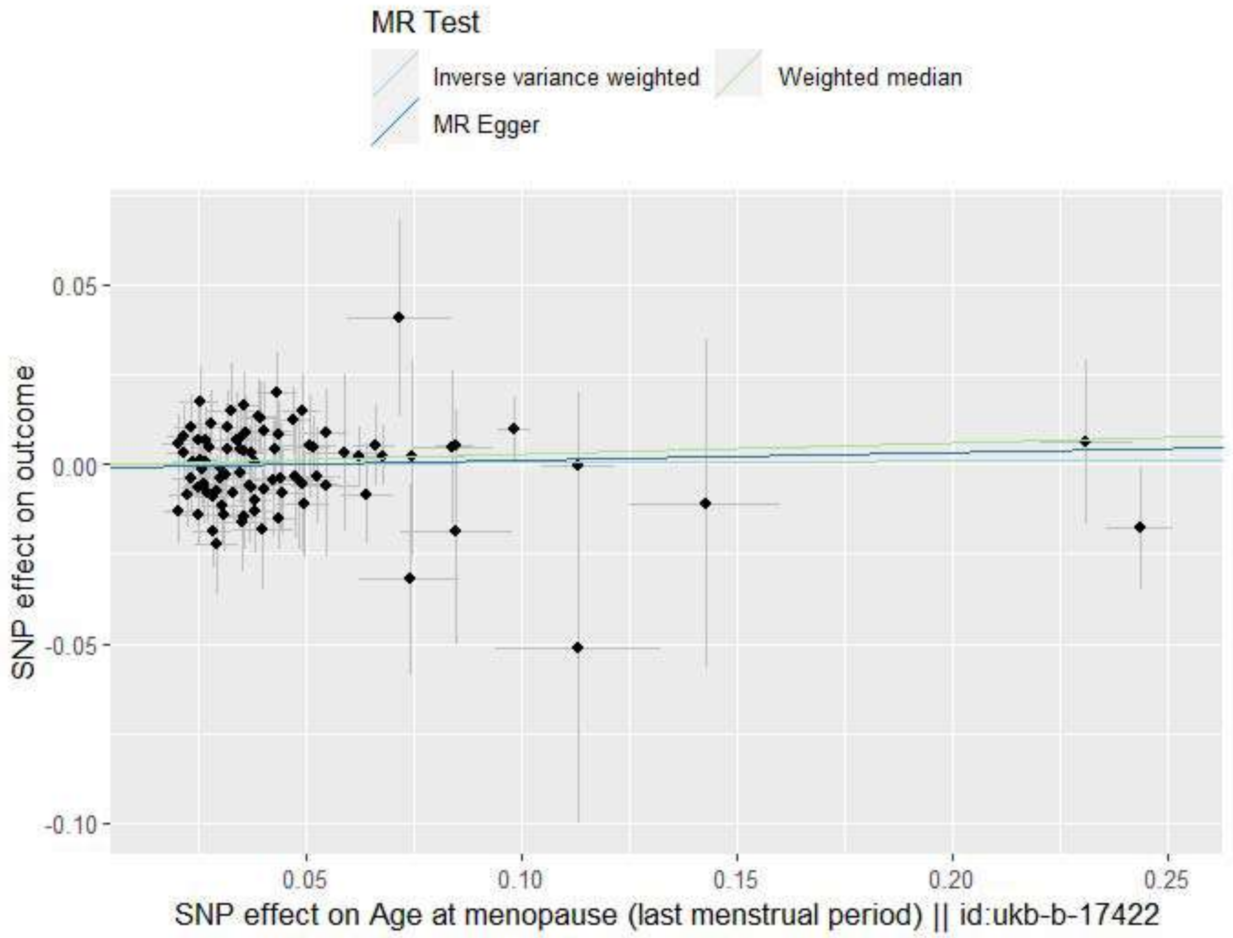

Supplementary figure 1 f

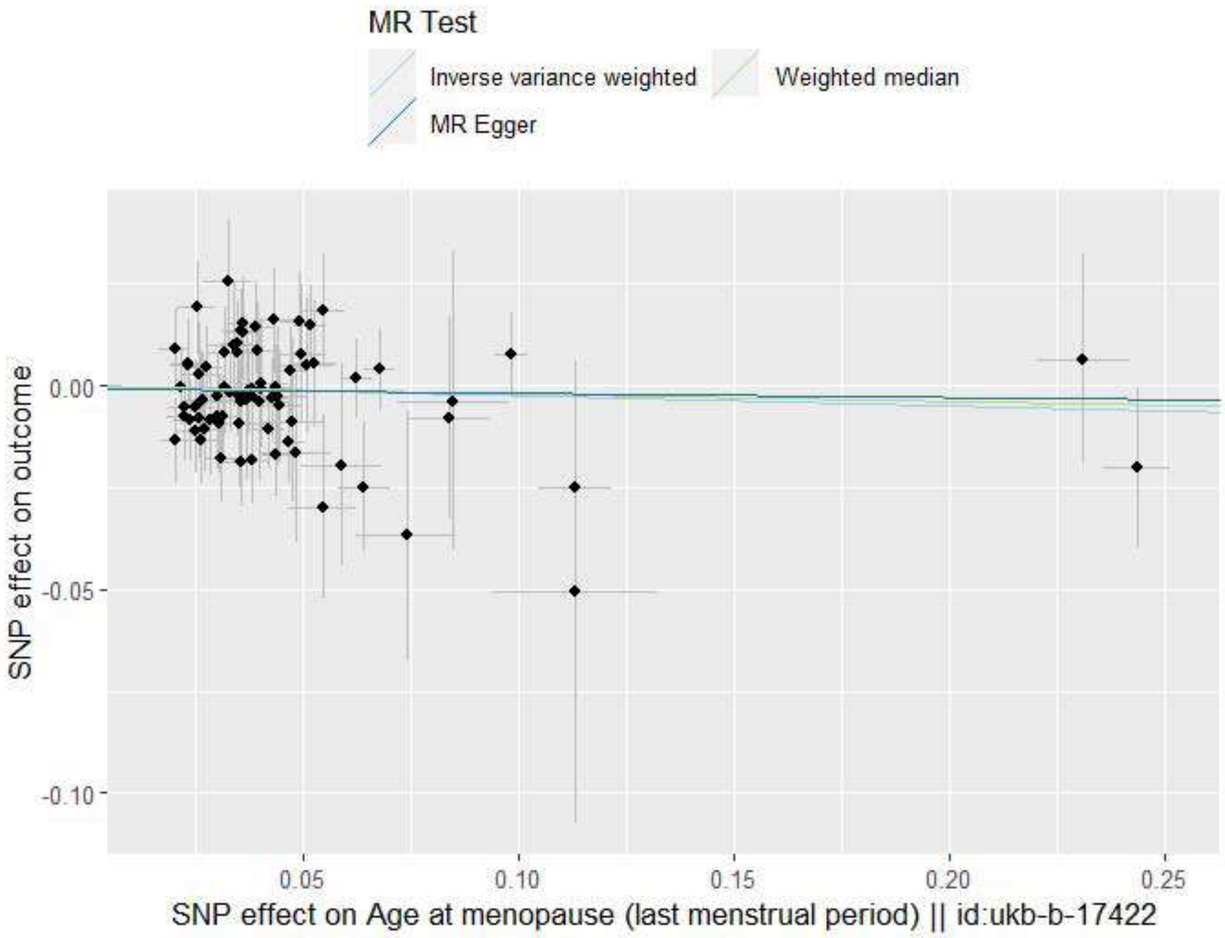

Supplementary figure 1 g

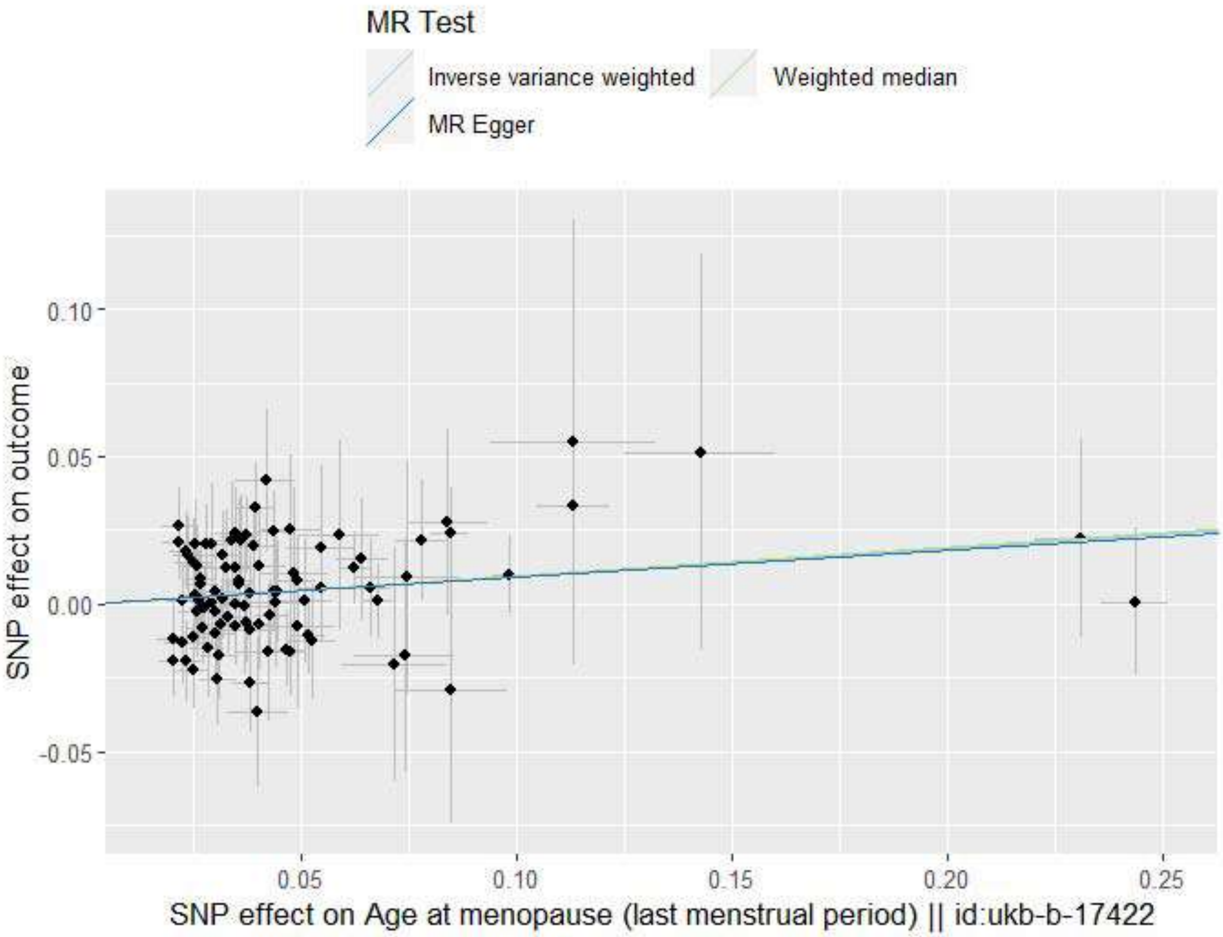

Supplementary figure 1 h

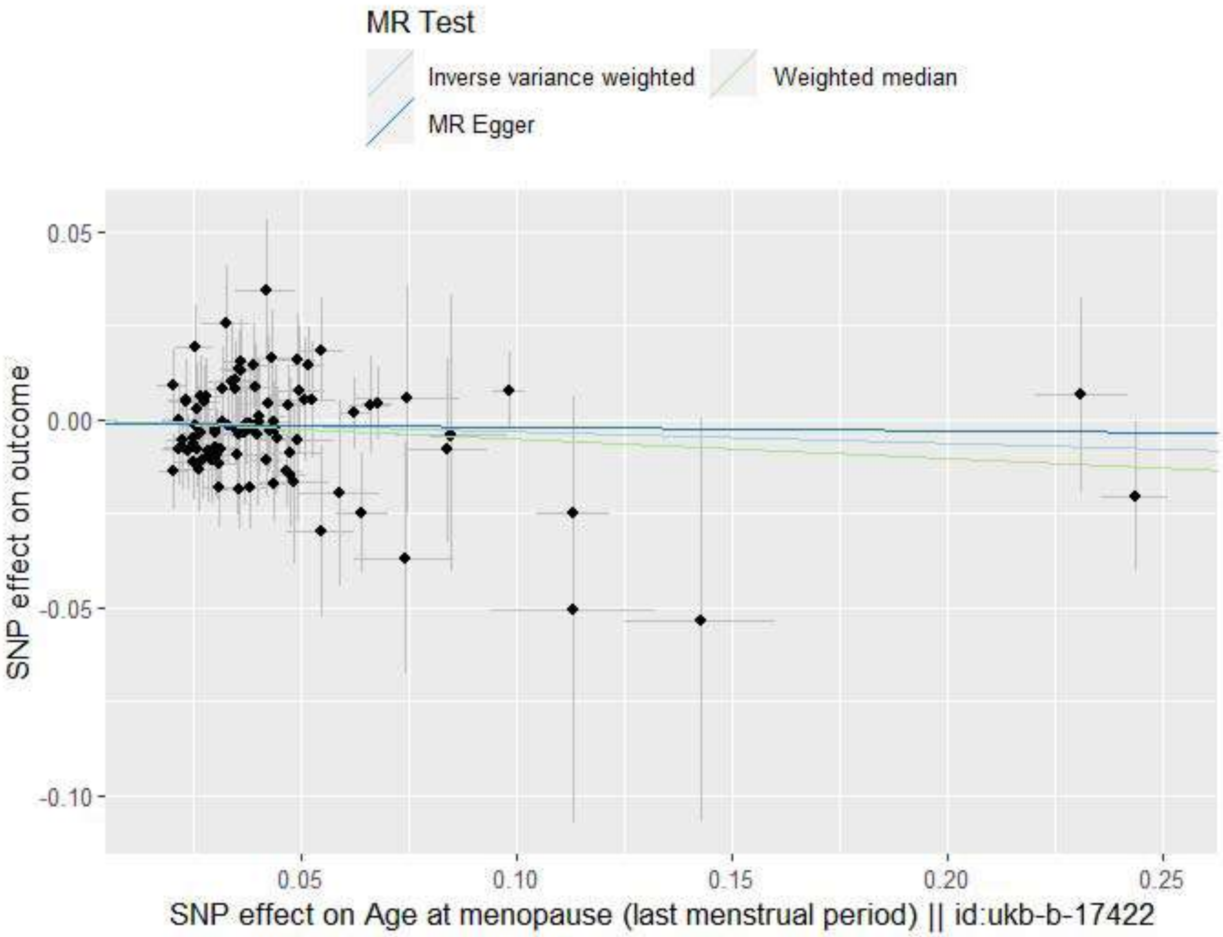

Supplementary figure 1 i

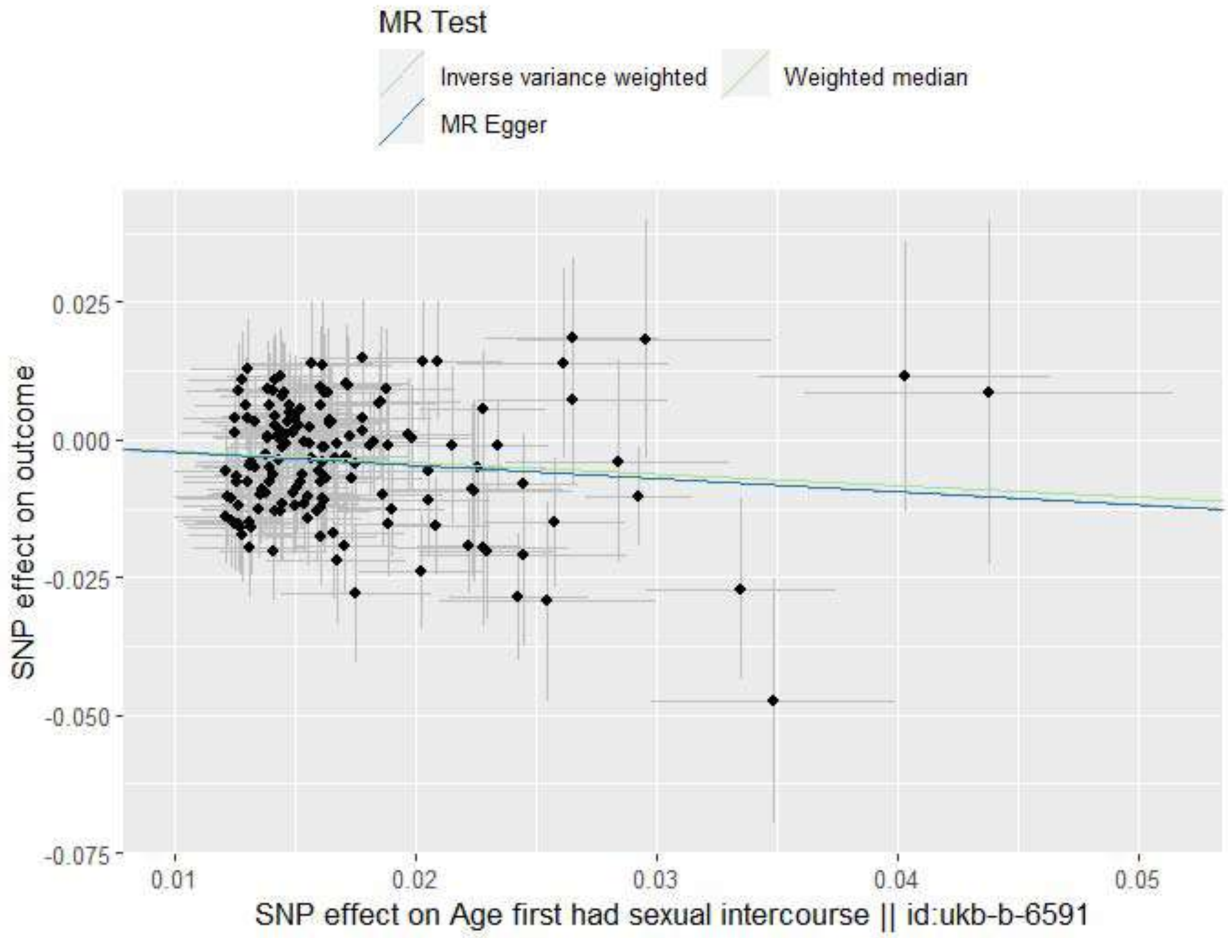

Supplementary figure 1 j

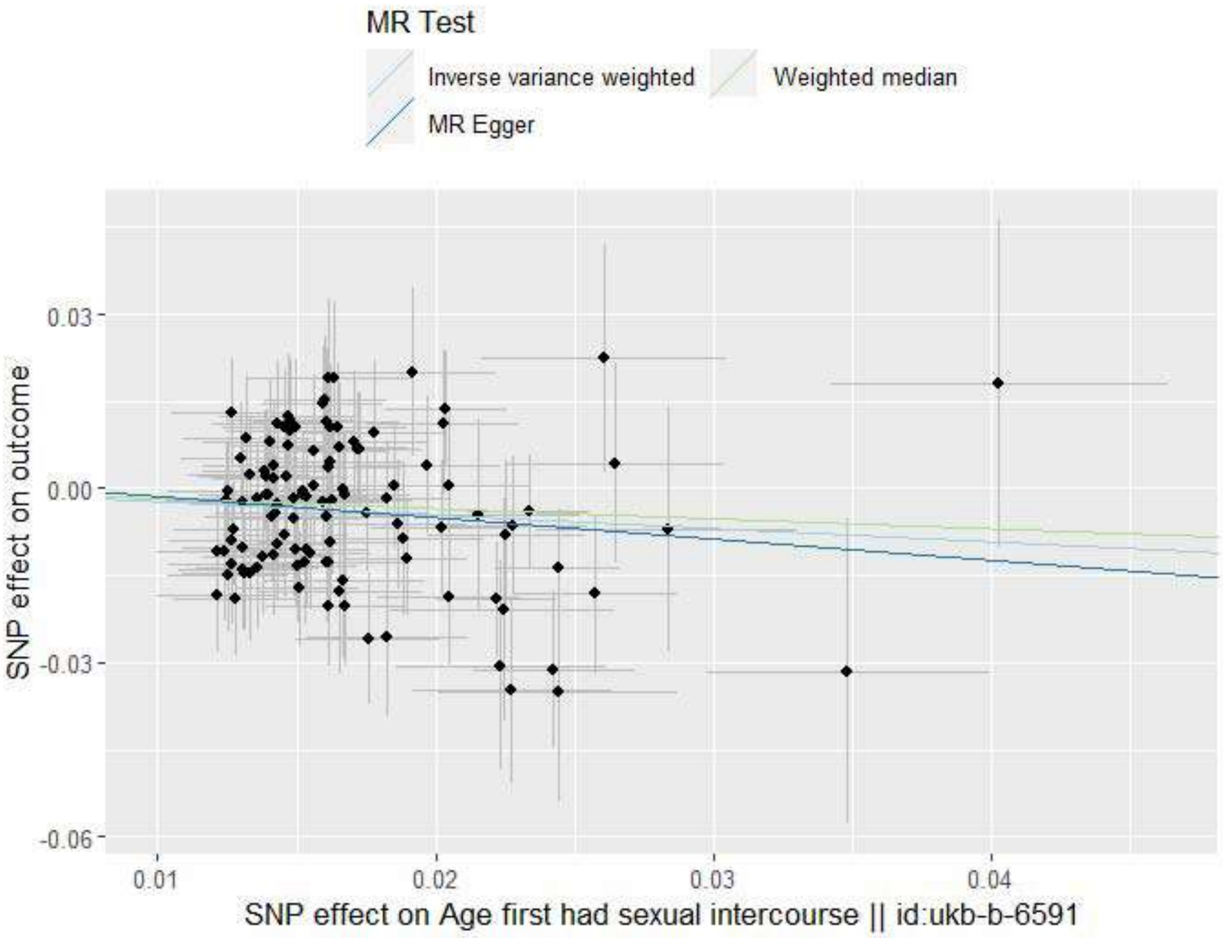

Supplementary figure 1 k

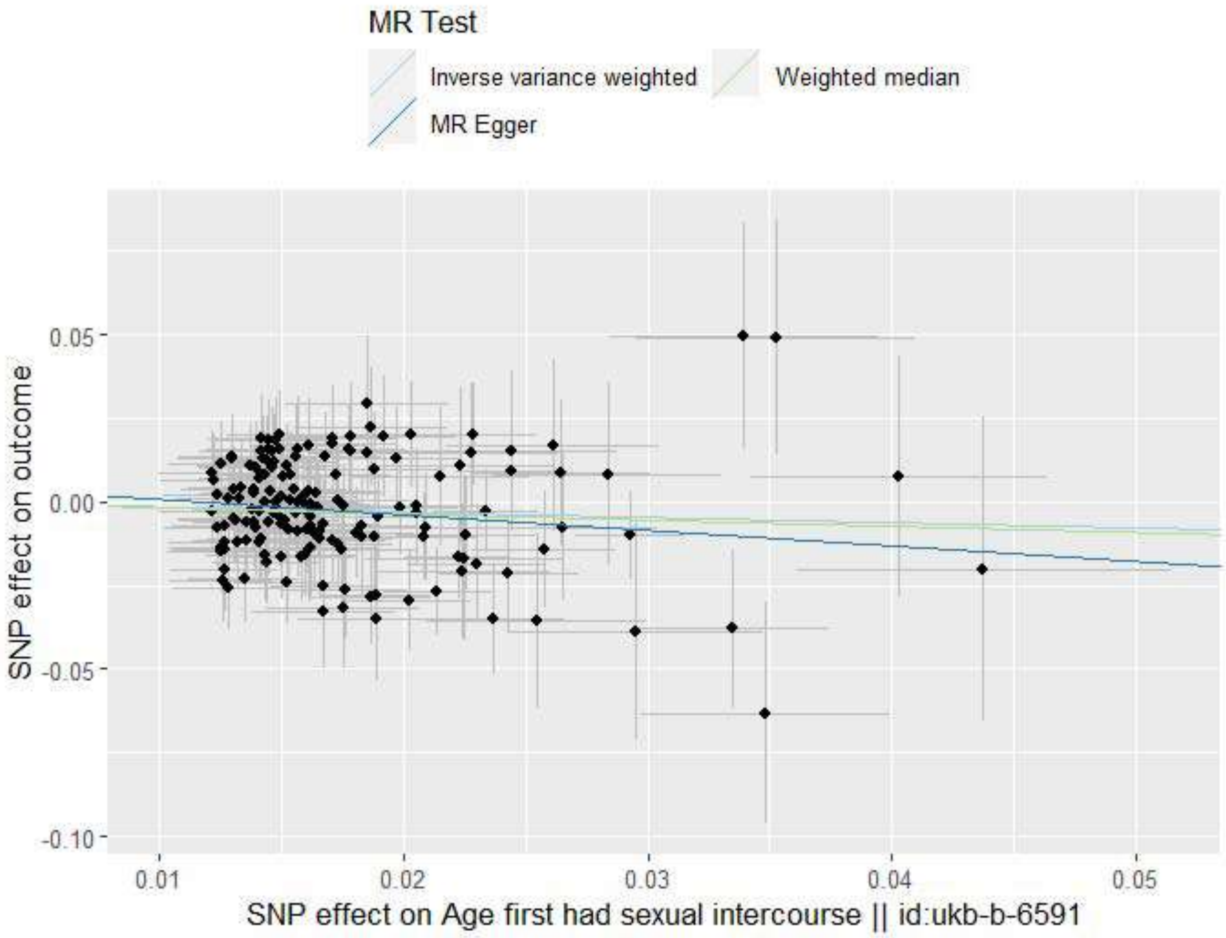

Supplementary figure 1 l

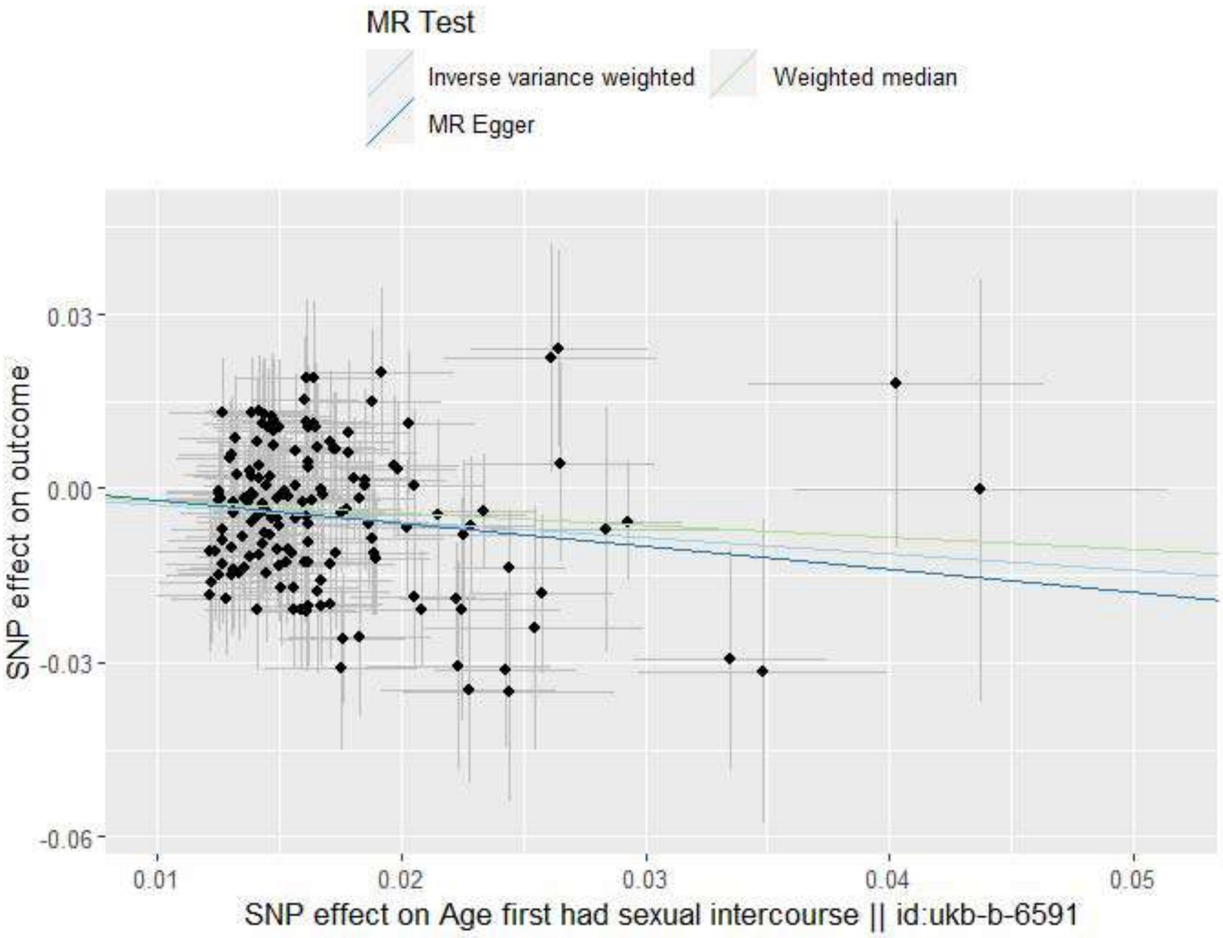

Supplementary figure 1 m

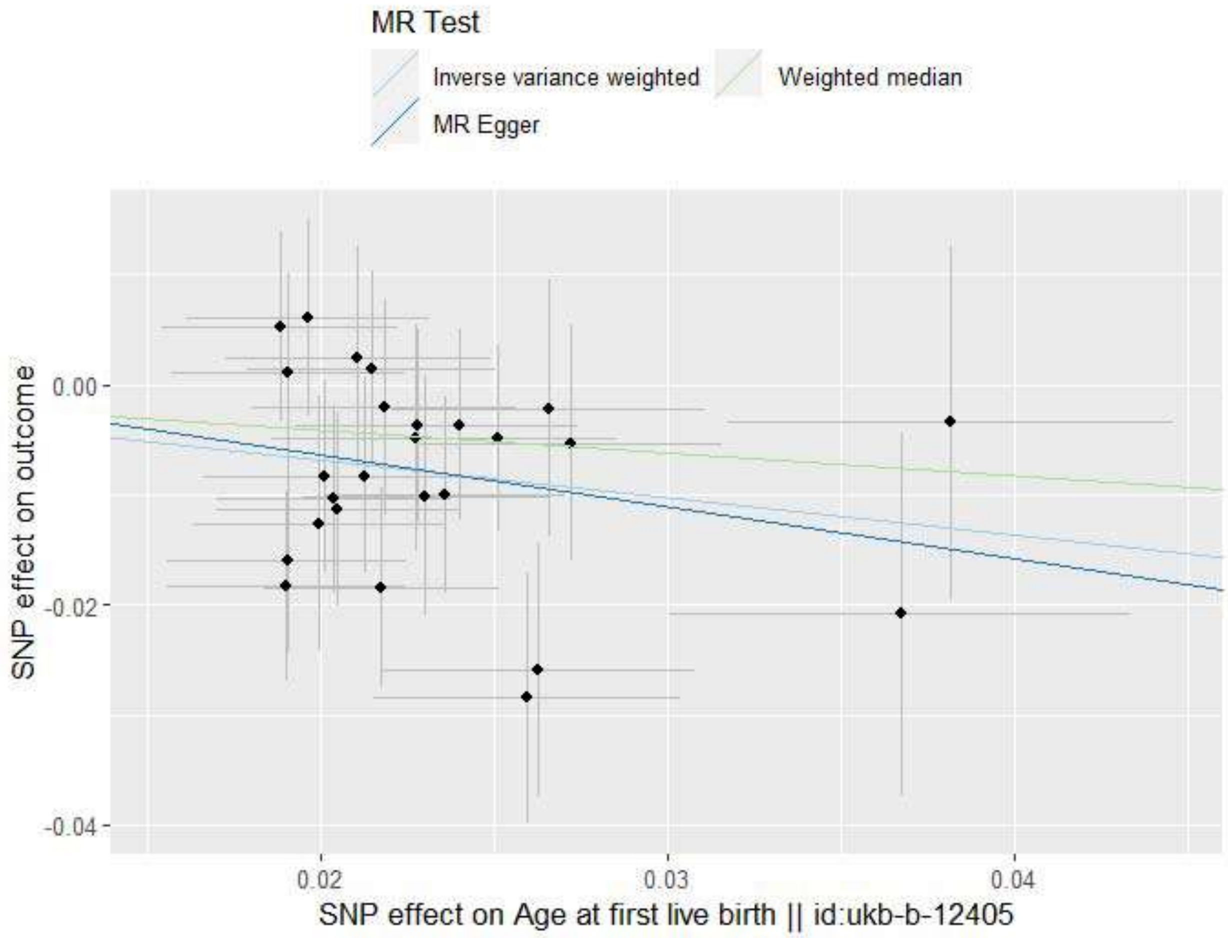

Supplementary figure 1 n

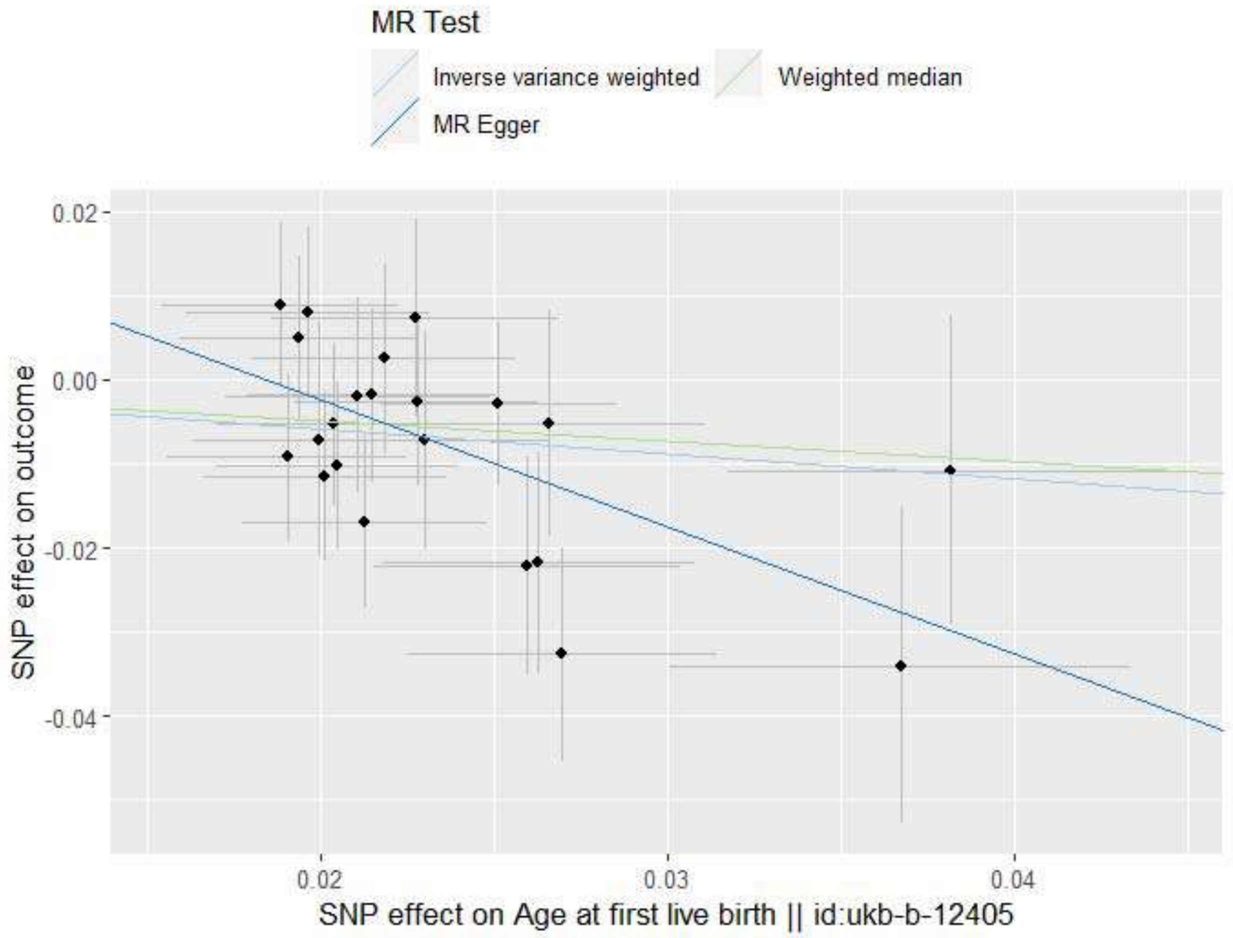

Supplementary figure 1 o

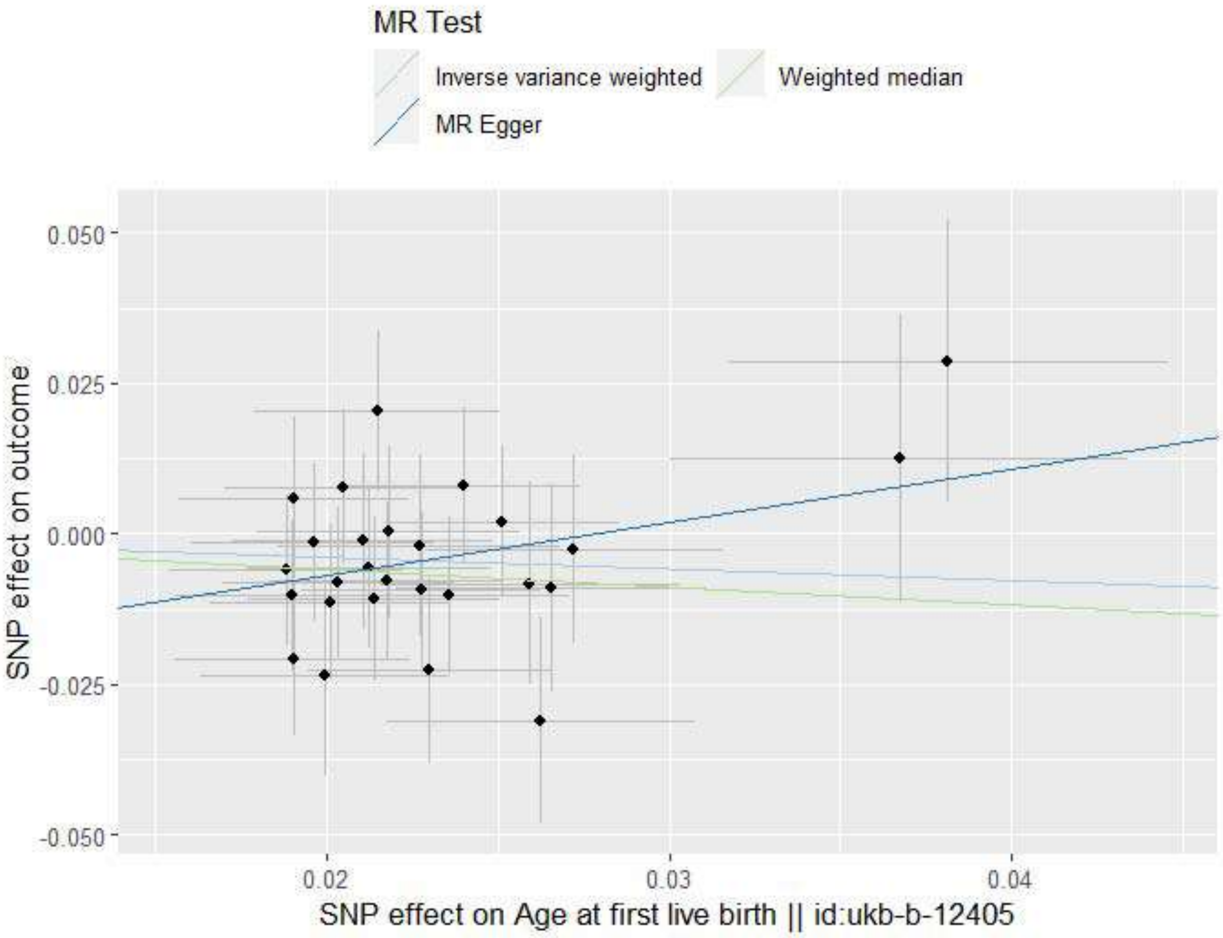

Supplementary figure 1 p

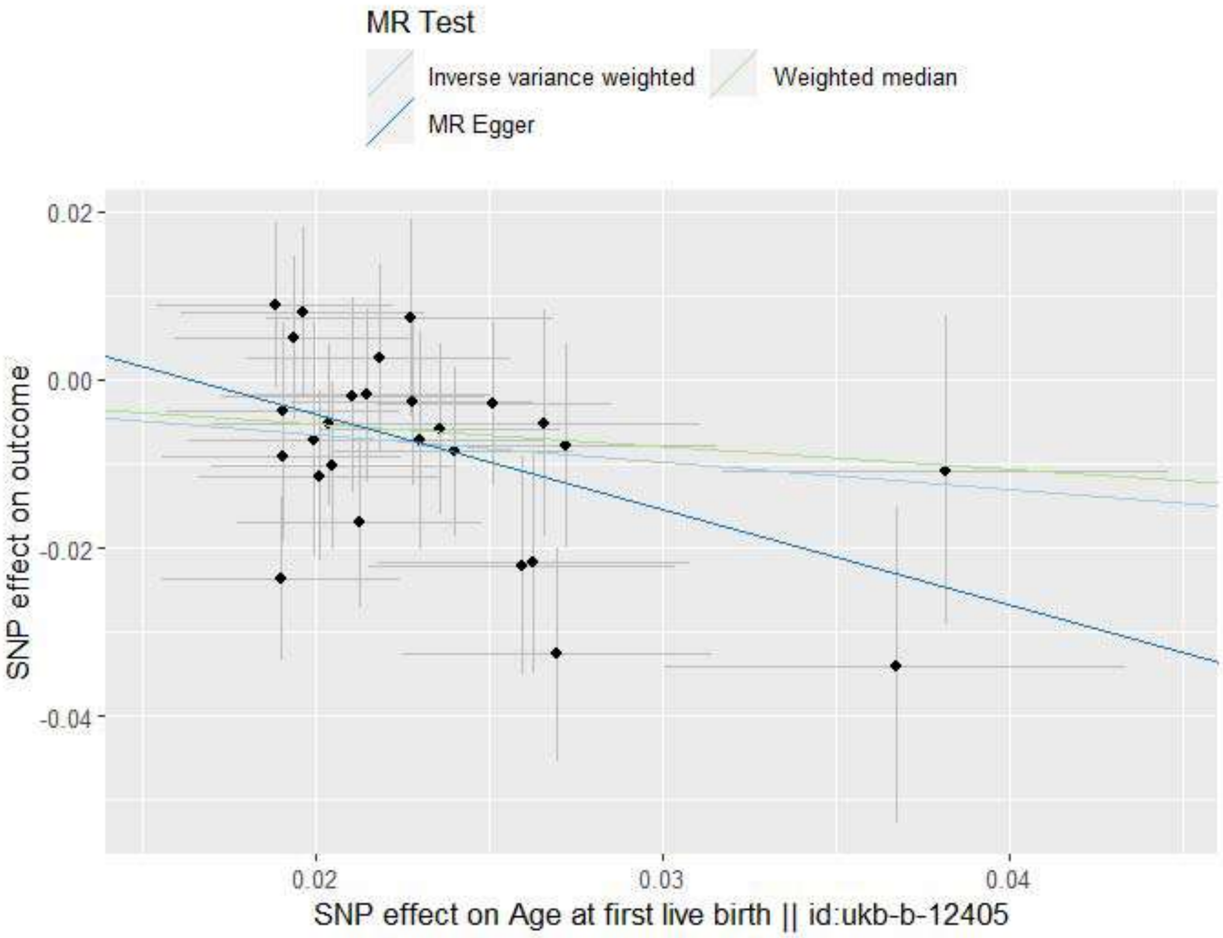

Supplementary figure 17 q

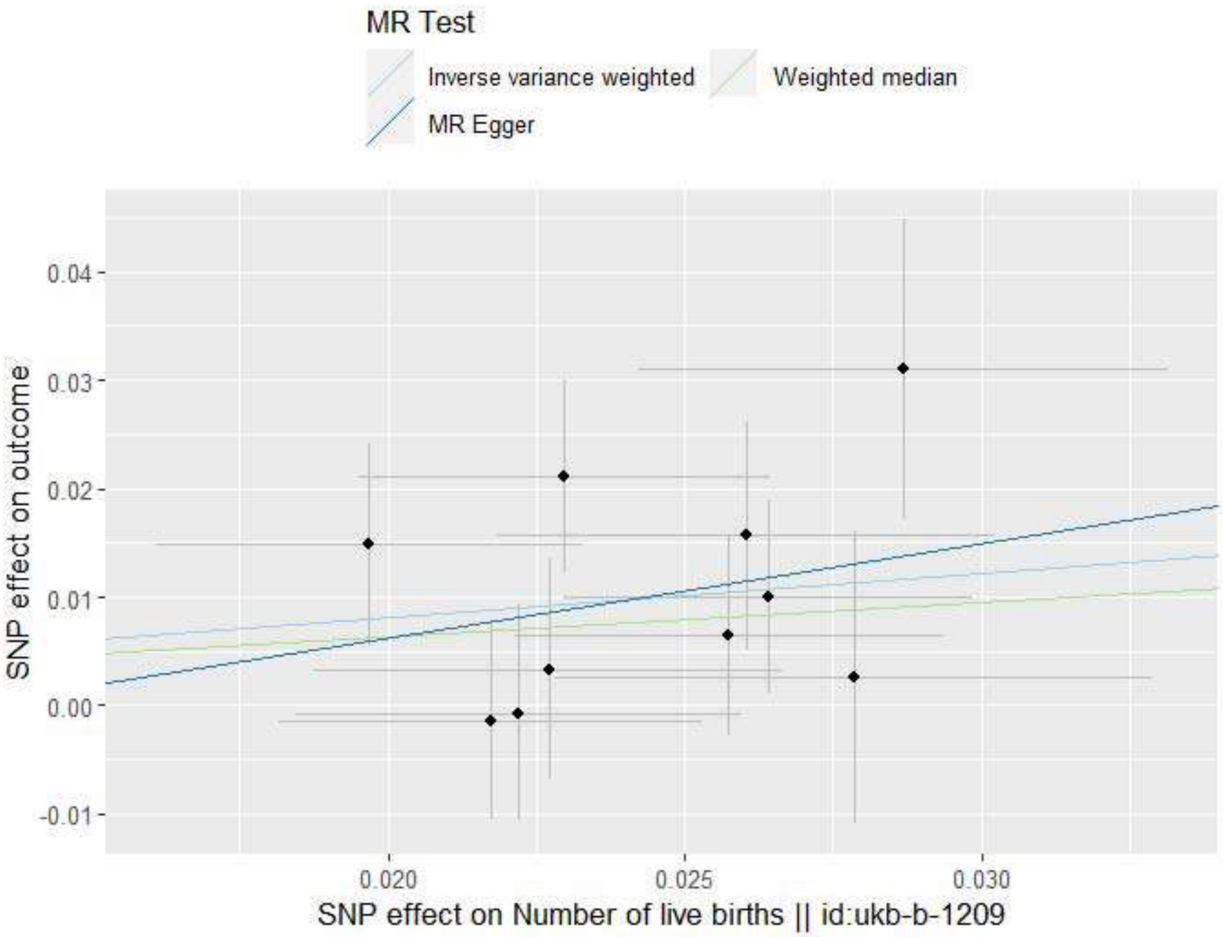

Supplementary figure 17 r

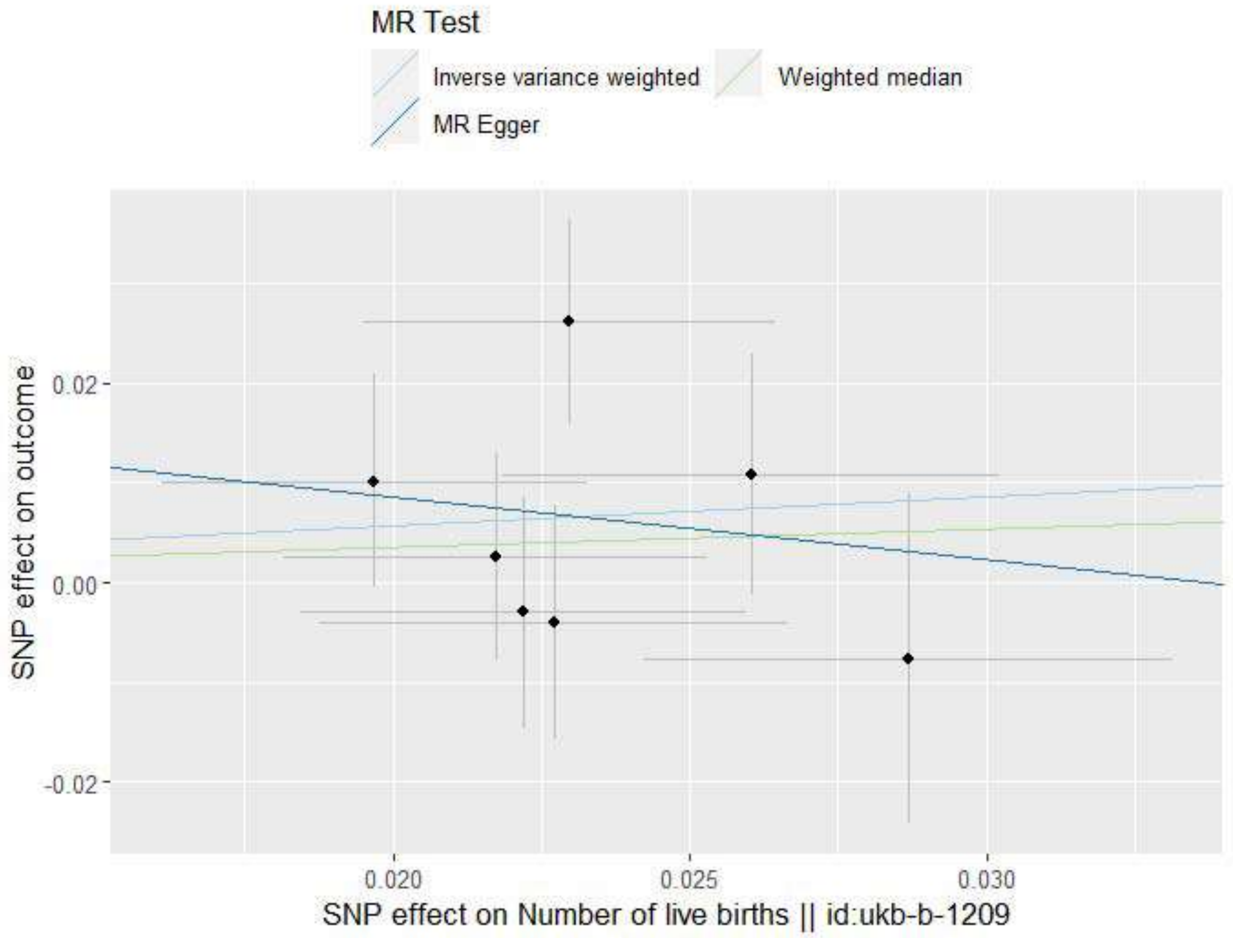

Supplementary figure 17 s

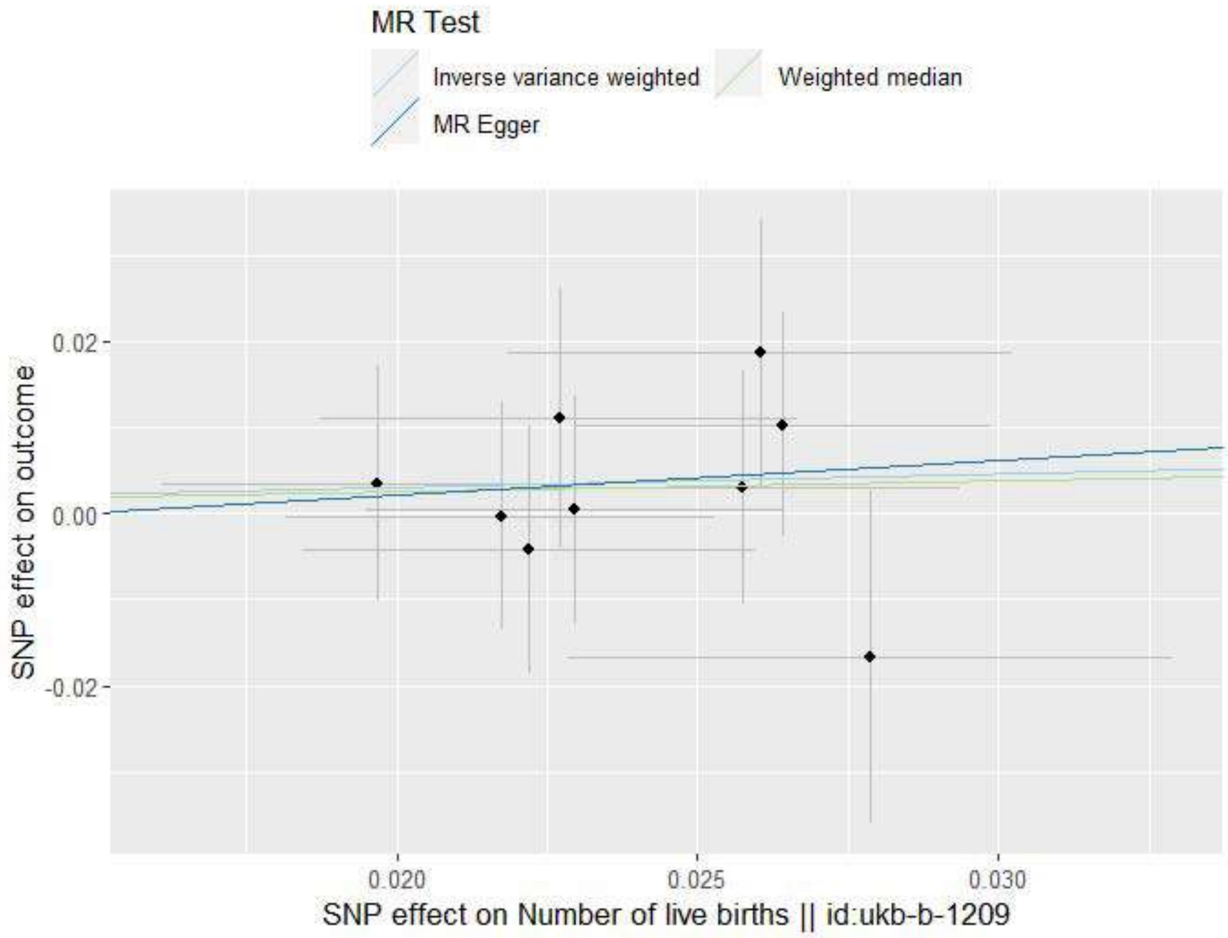

Supplementary figure 17 t

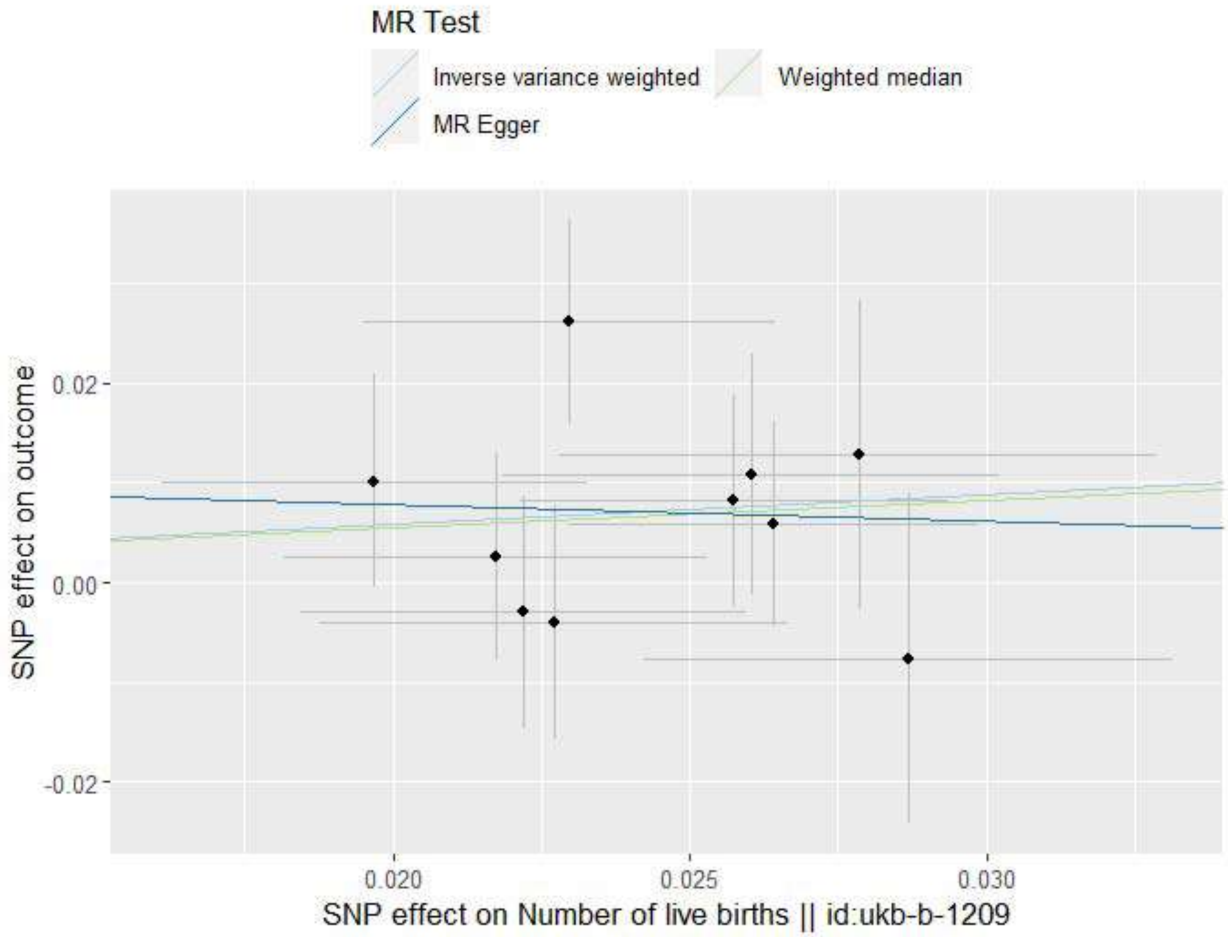

Supplementary figure 2 a

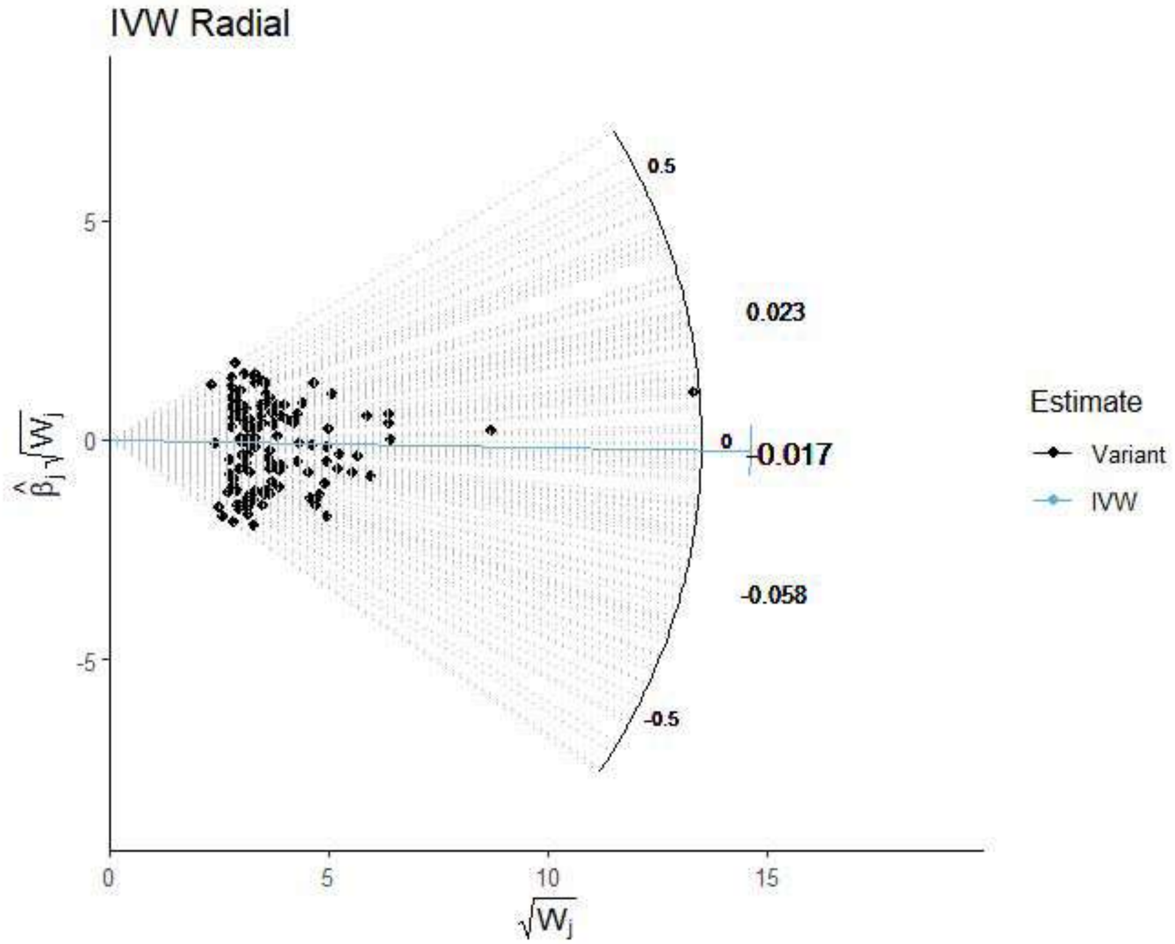

Supplementary figure 2 b

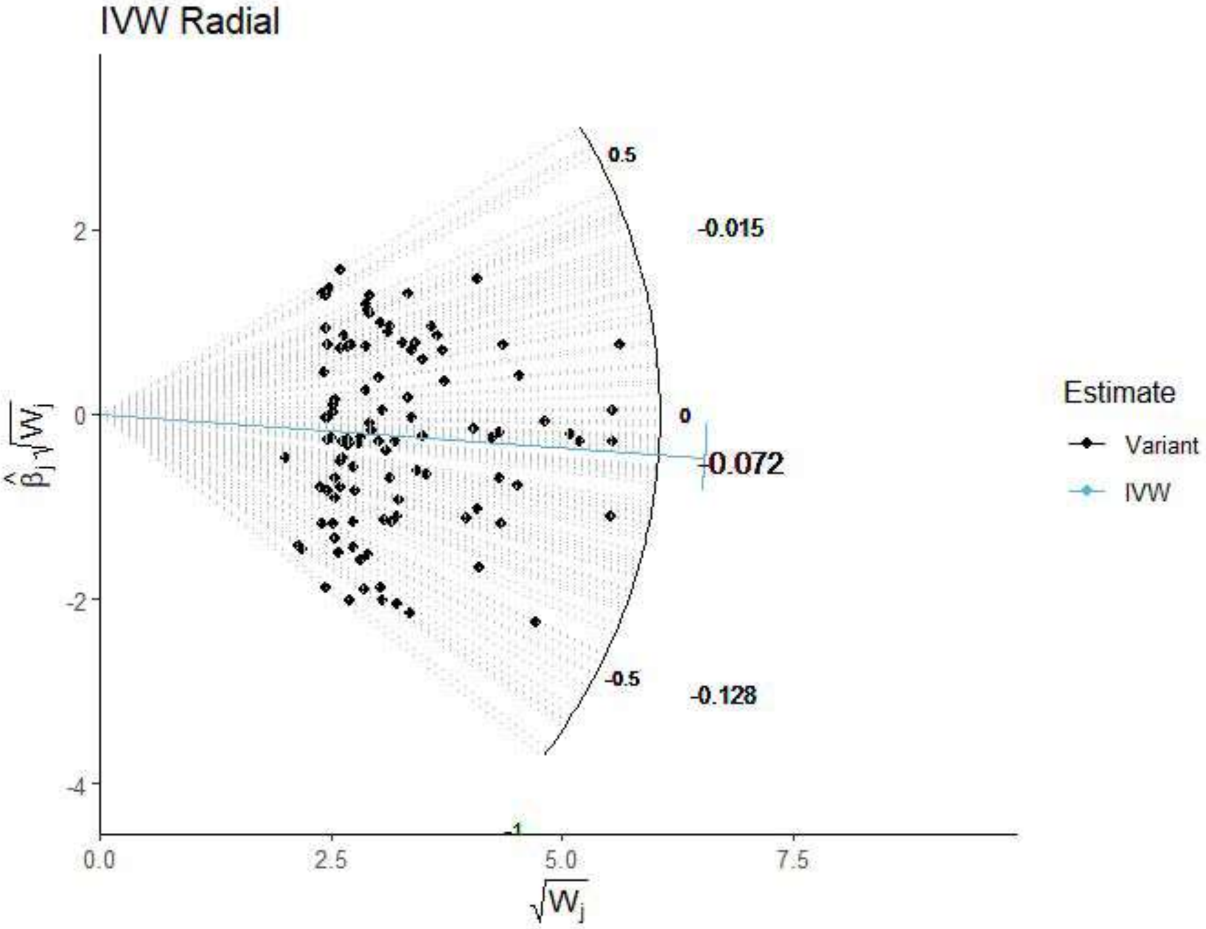

Supplementary figure 2 c

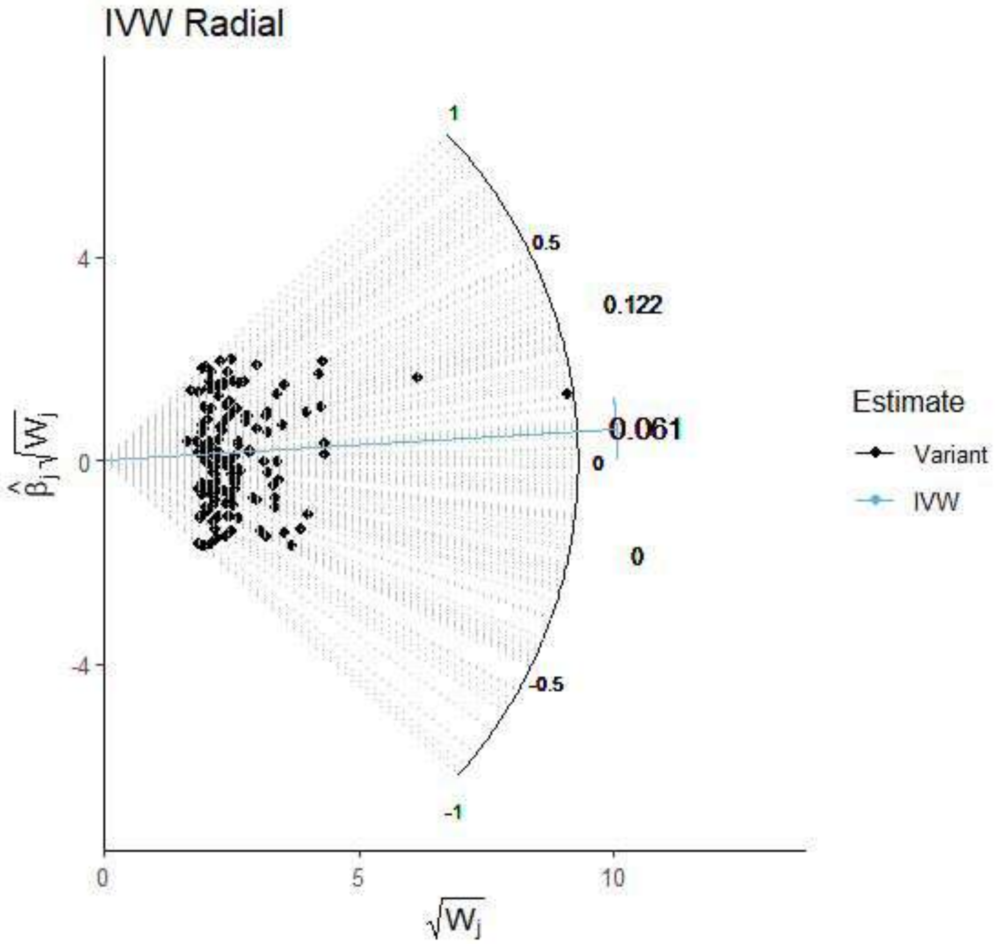

Supplementary figure 2 d

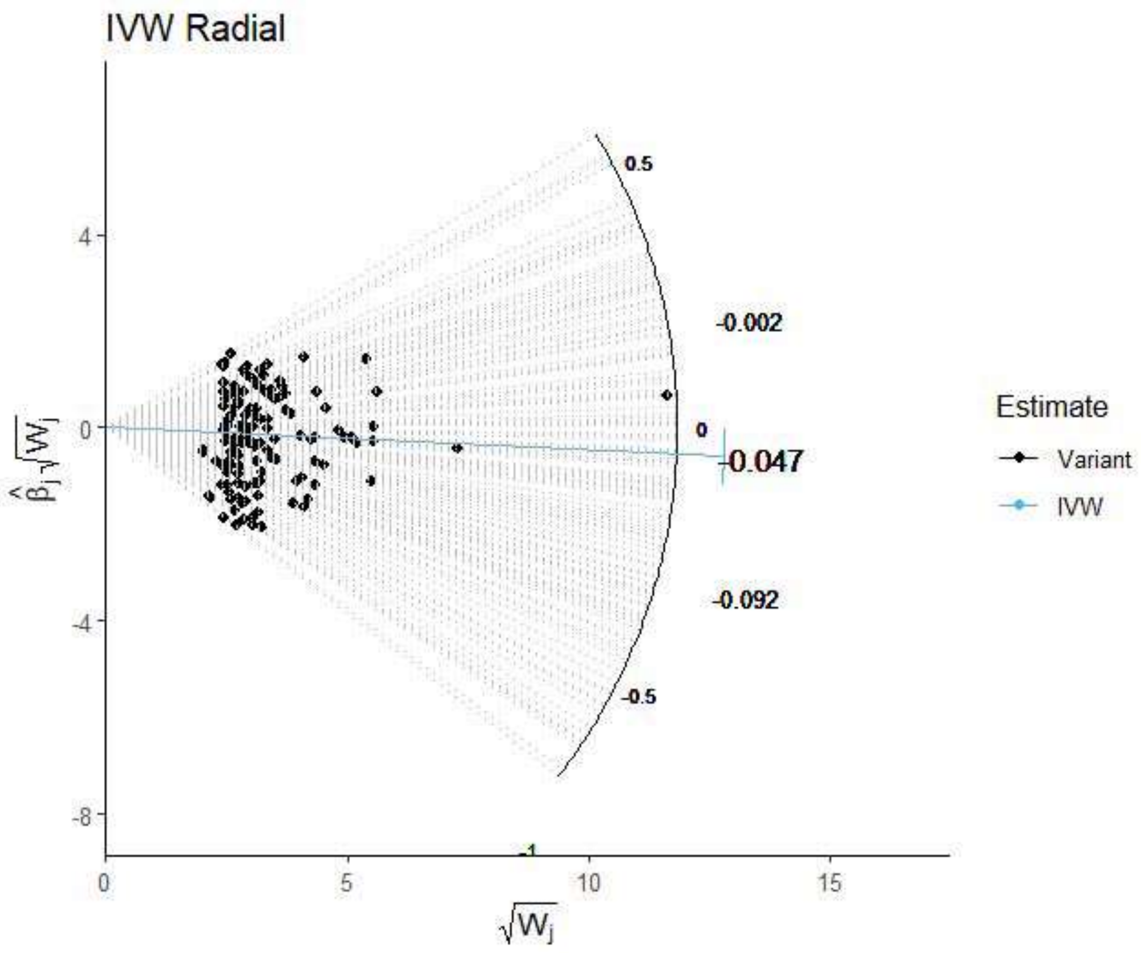

Supplementary figure 2 e

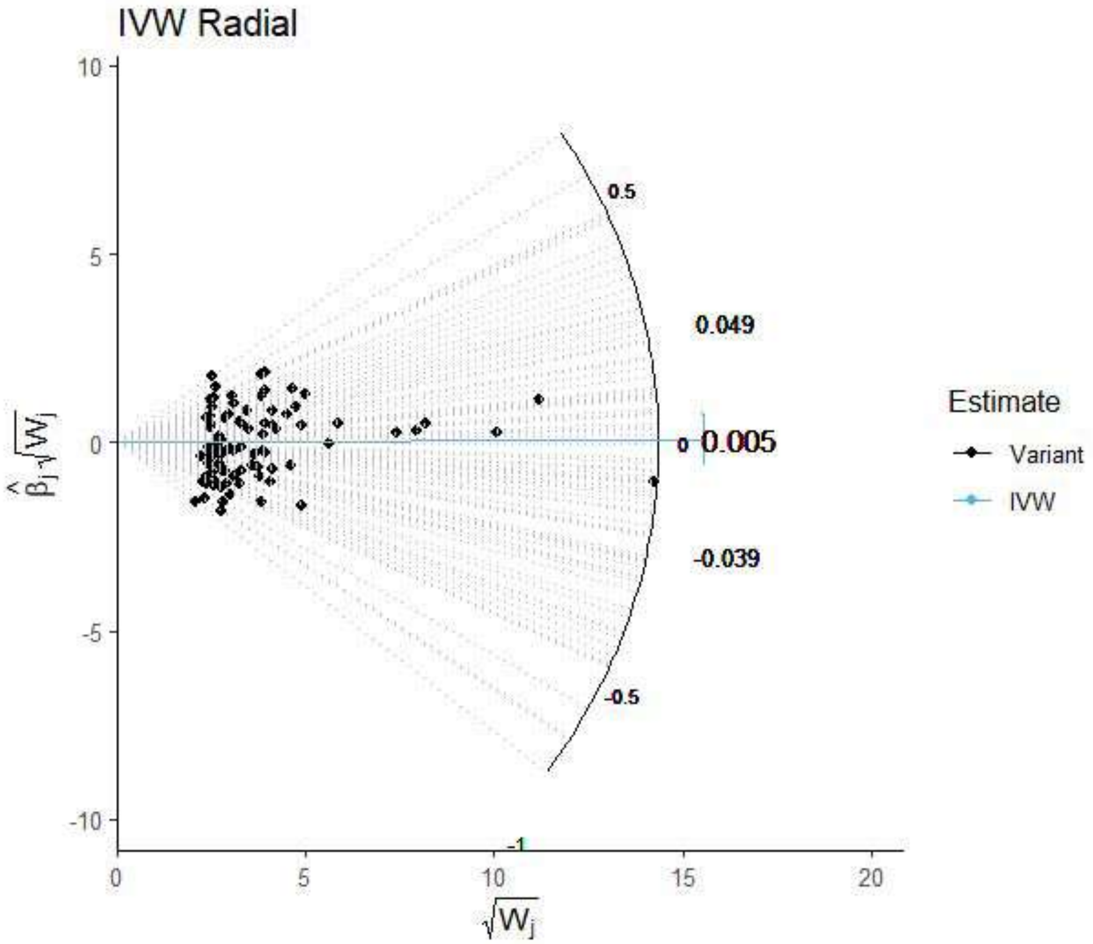

Supplementary figure 2 f

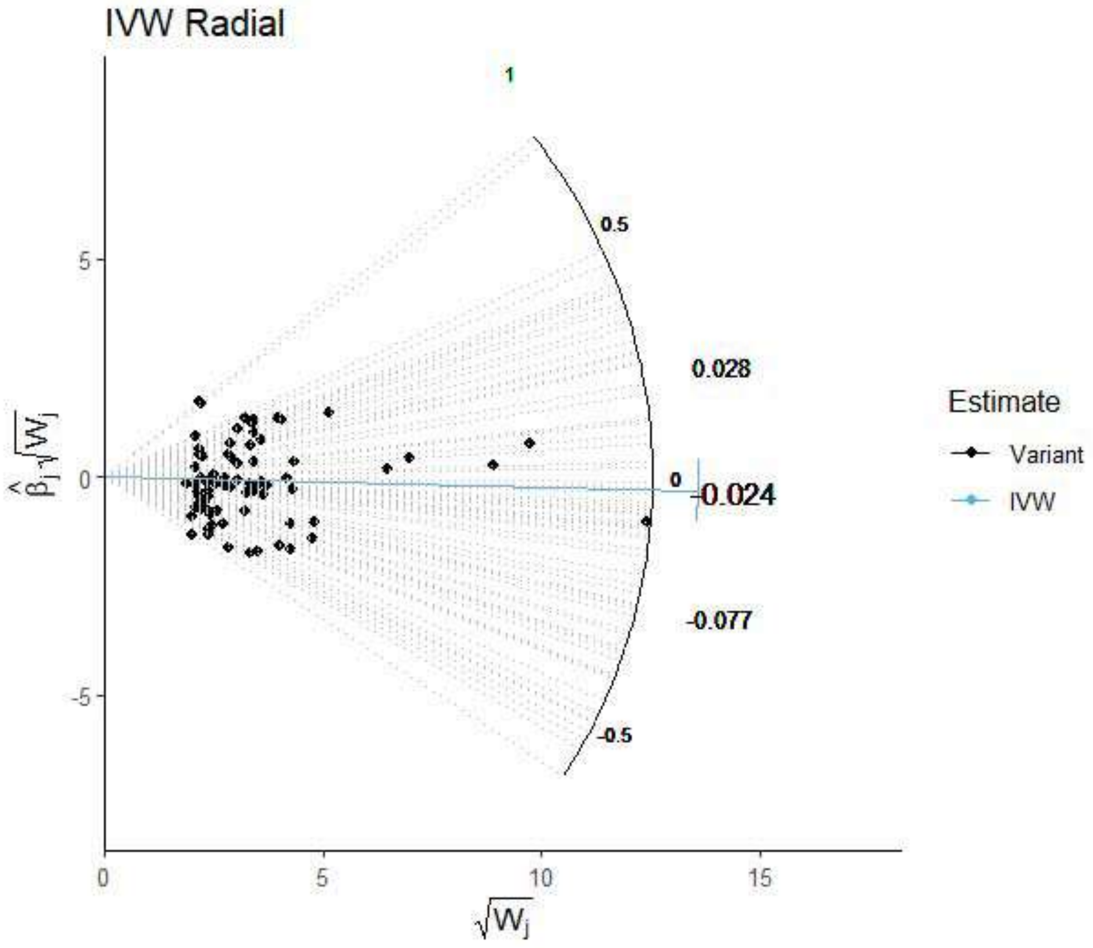

Supplementary figure 2 g

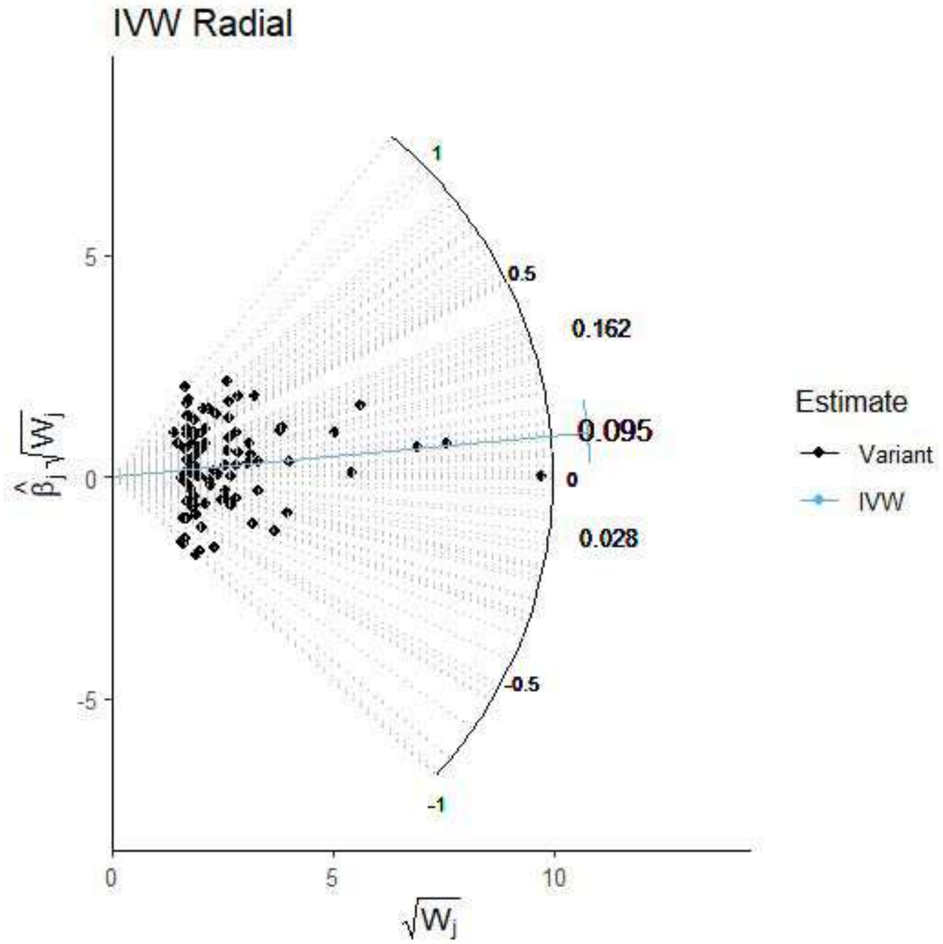

Supplementary figure 2 h

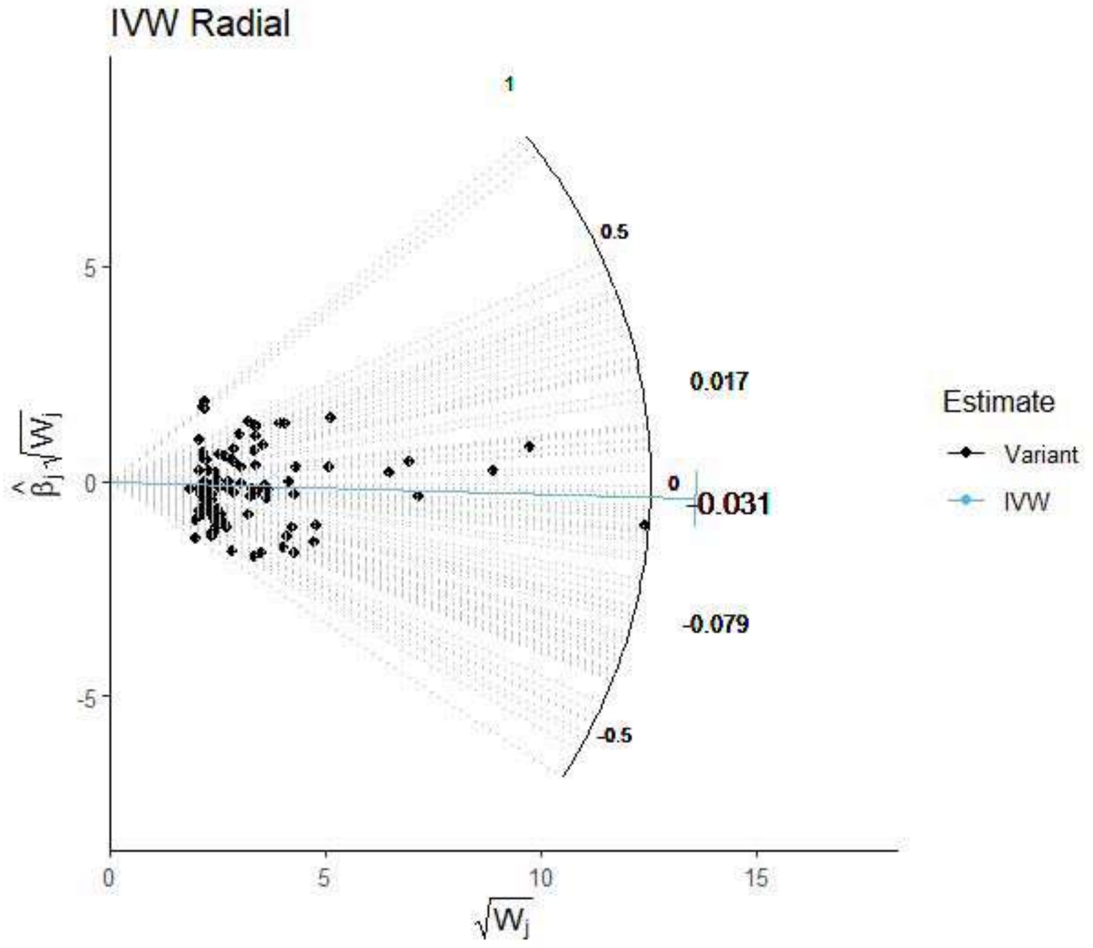

Supplementary figure 2 i

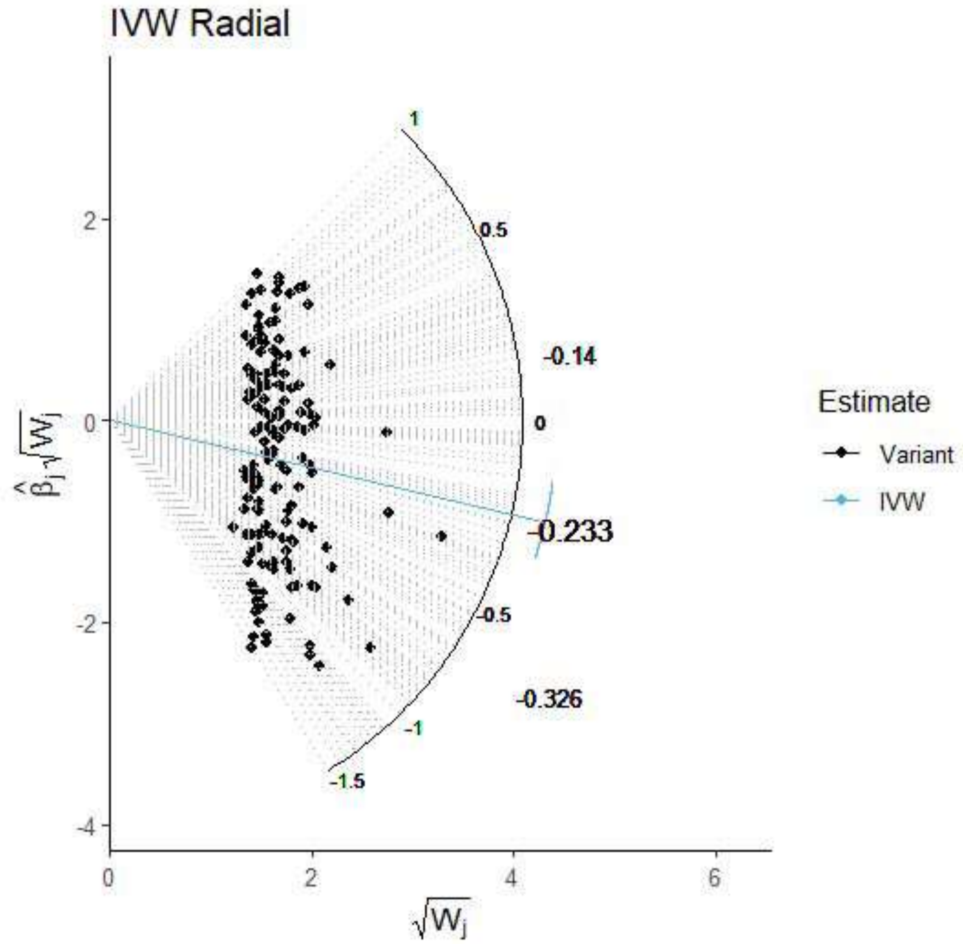

Supplementary figure 2 j

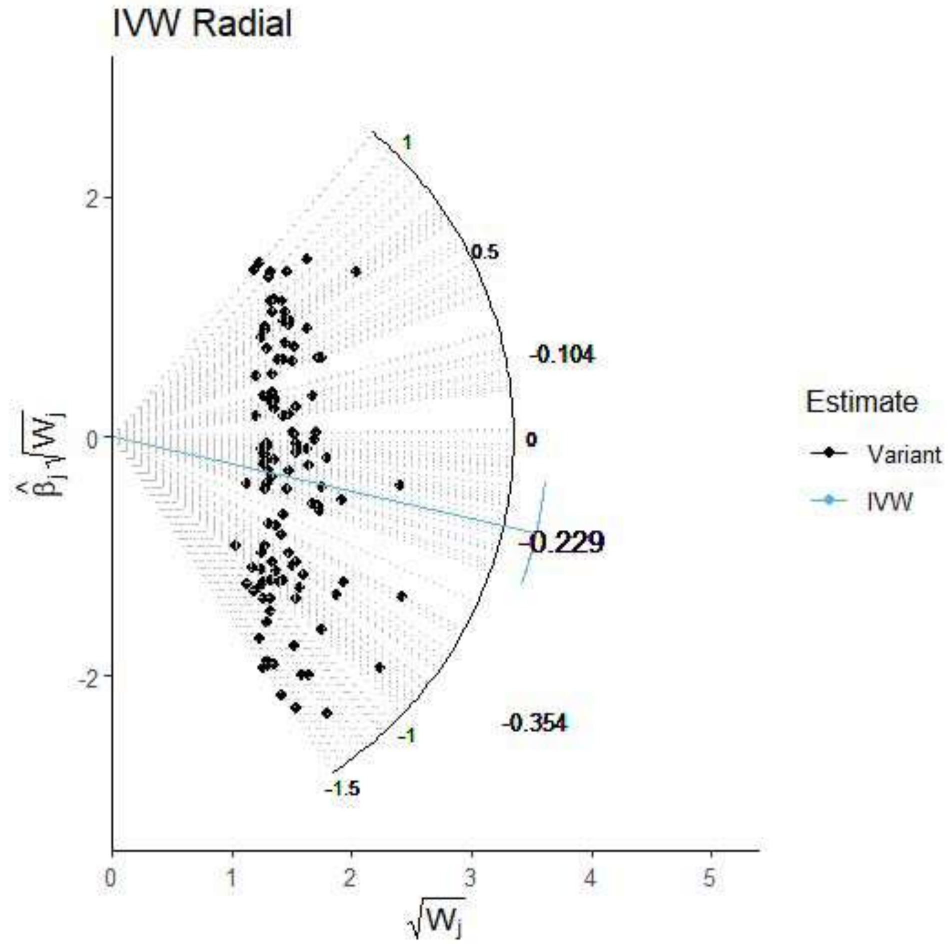

Supplementary figure 2 k

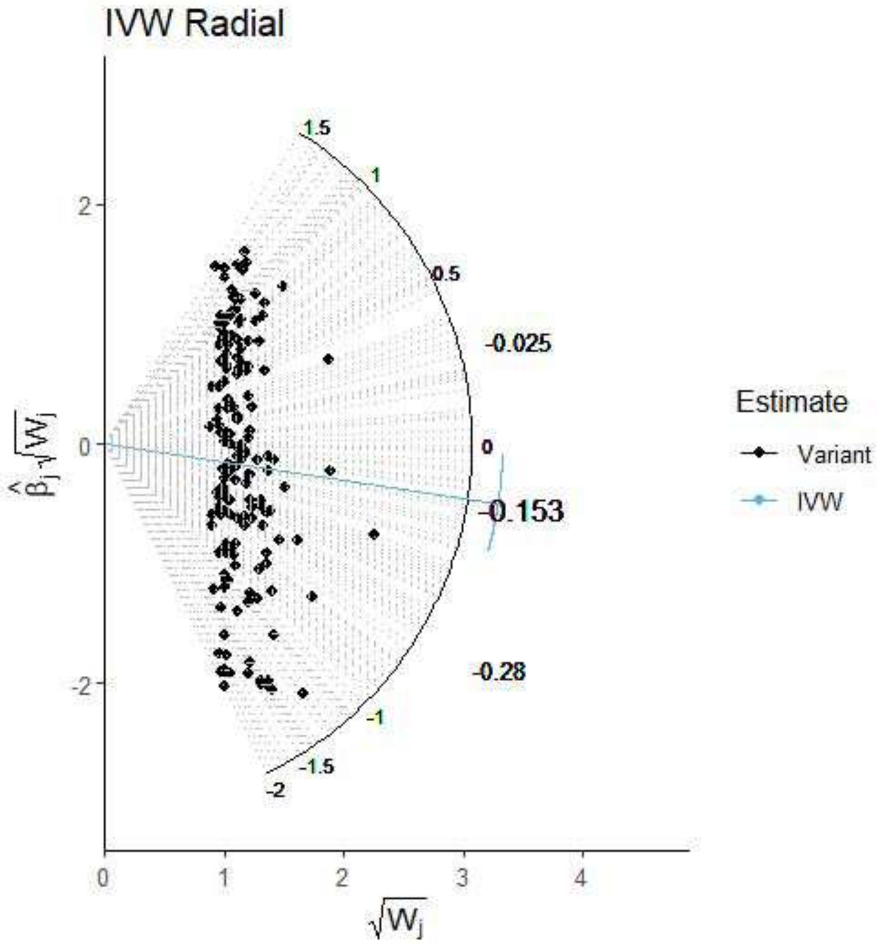

Supplementary figure 2 l

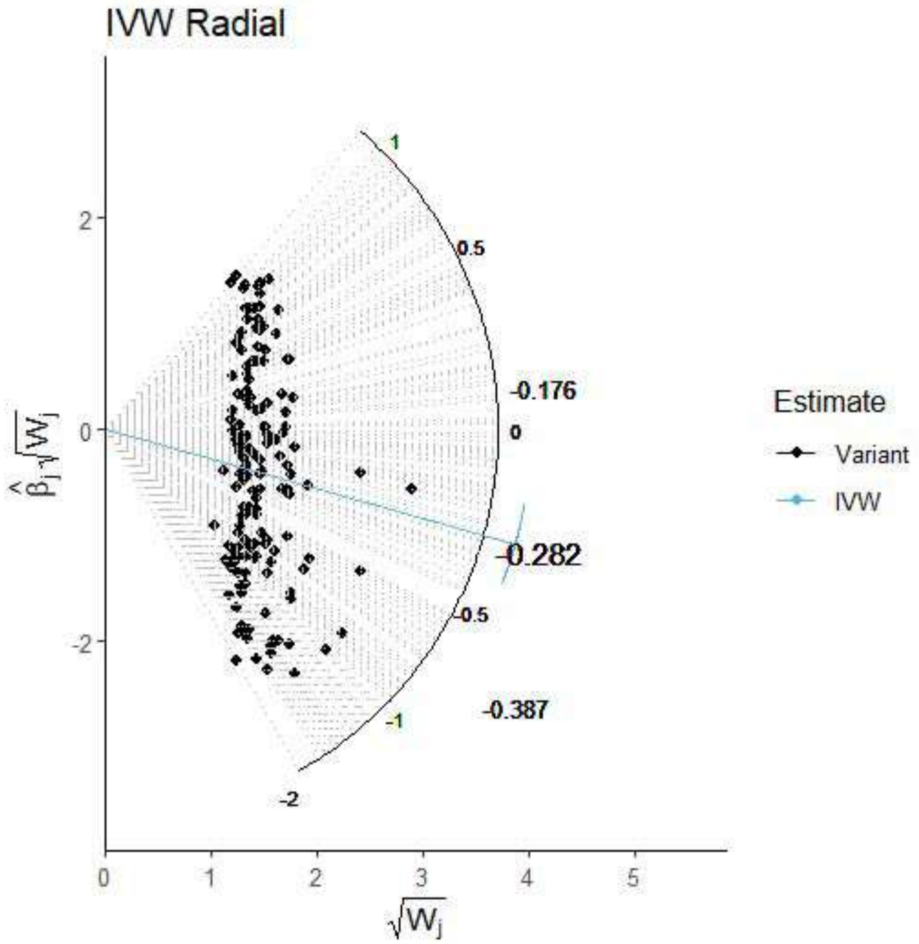

Supplementary figure 2 m

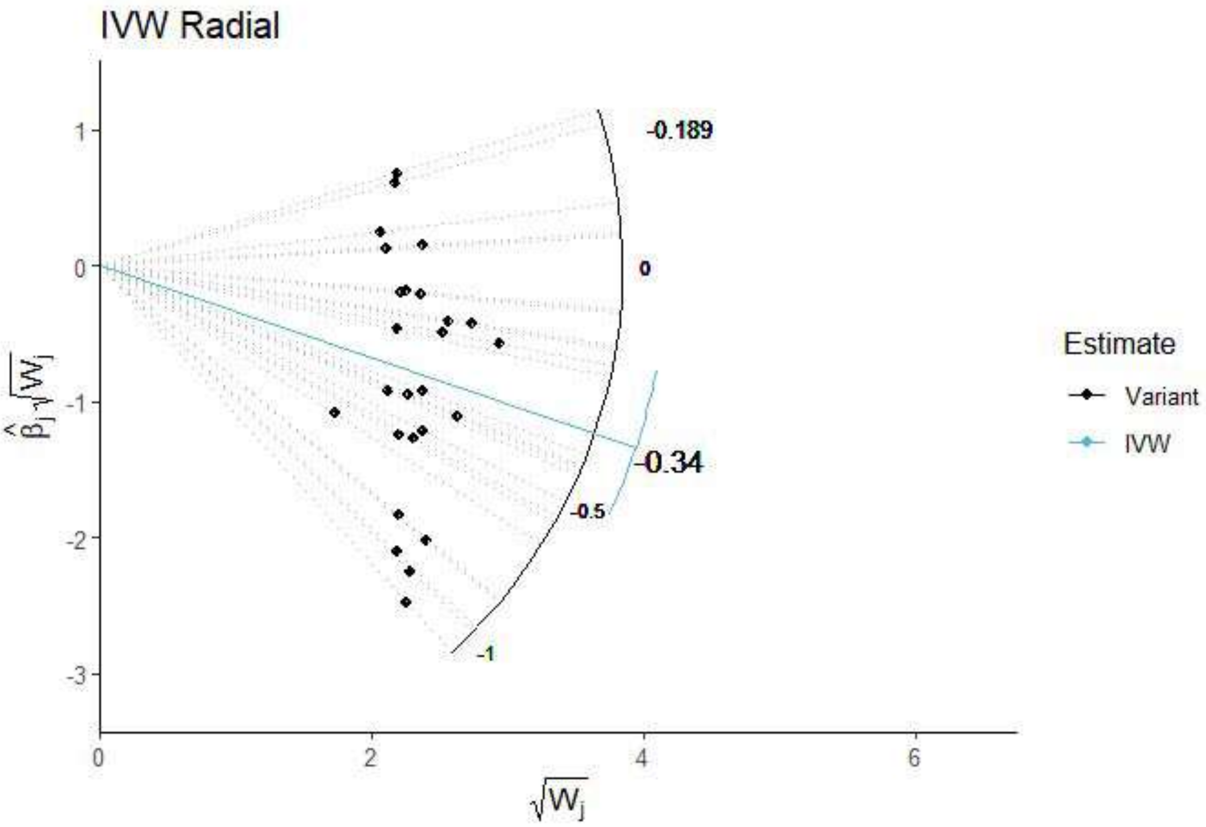

Supplementary figure 2n

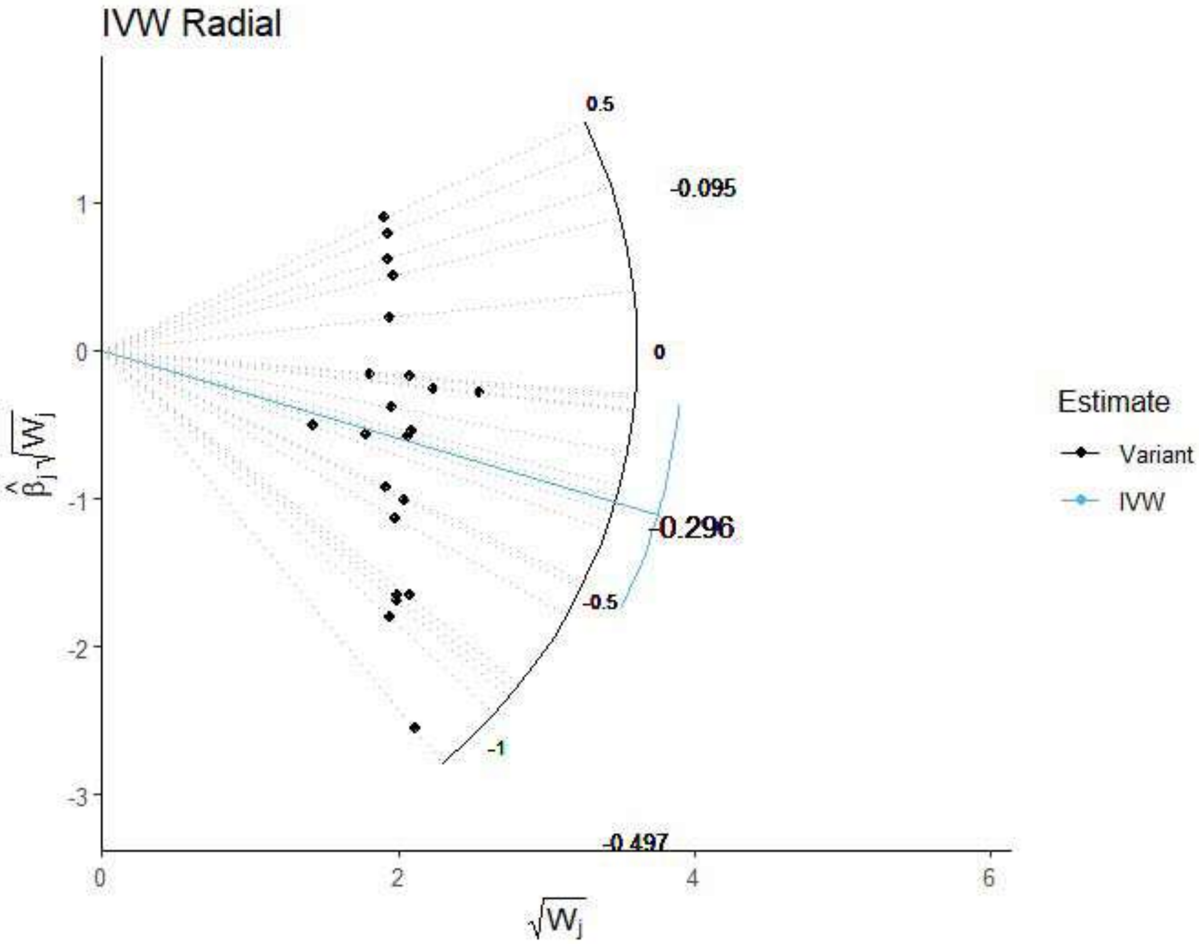

Supplementary figure 2 o

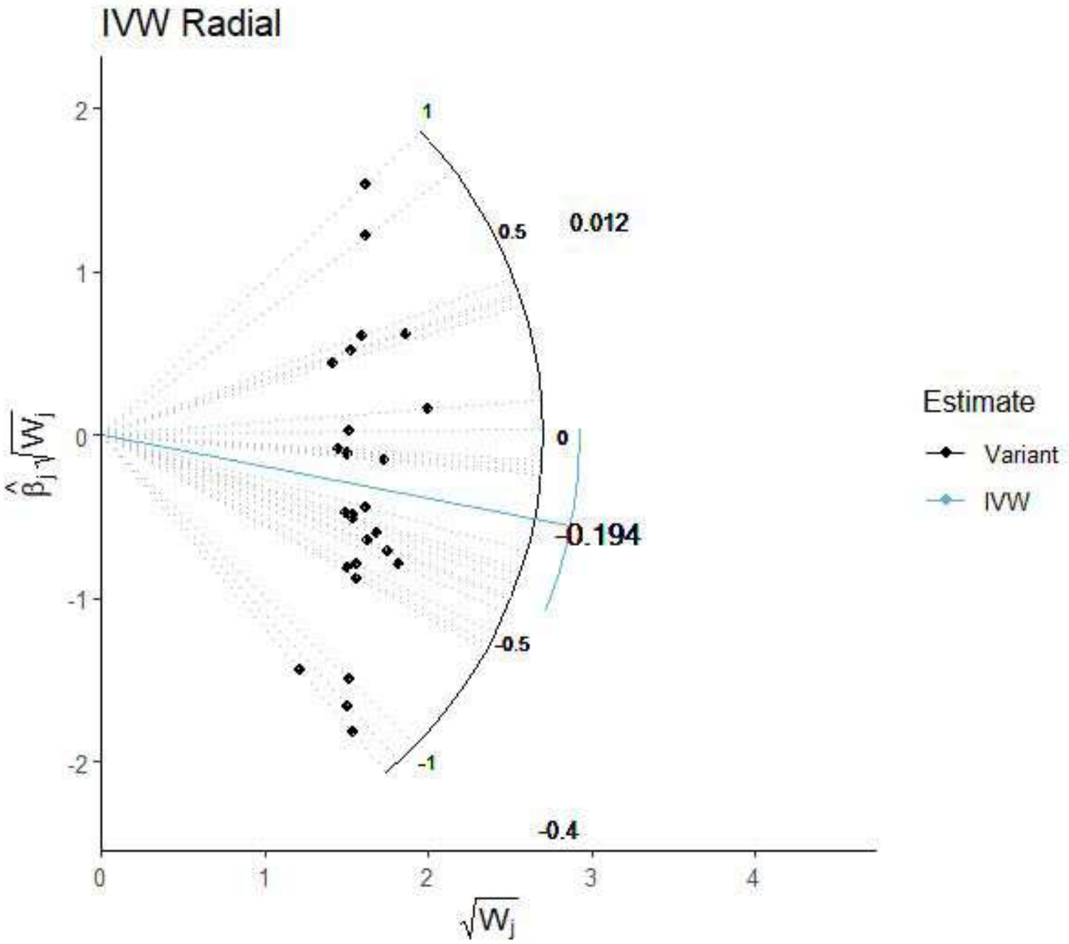

Supplementary figure 3 p

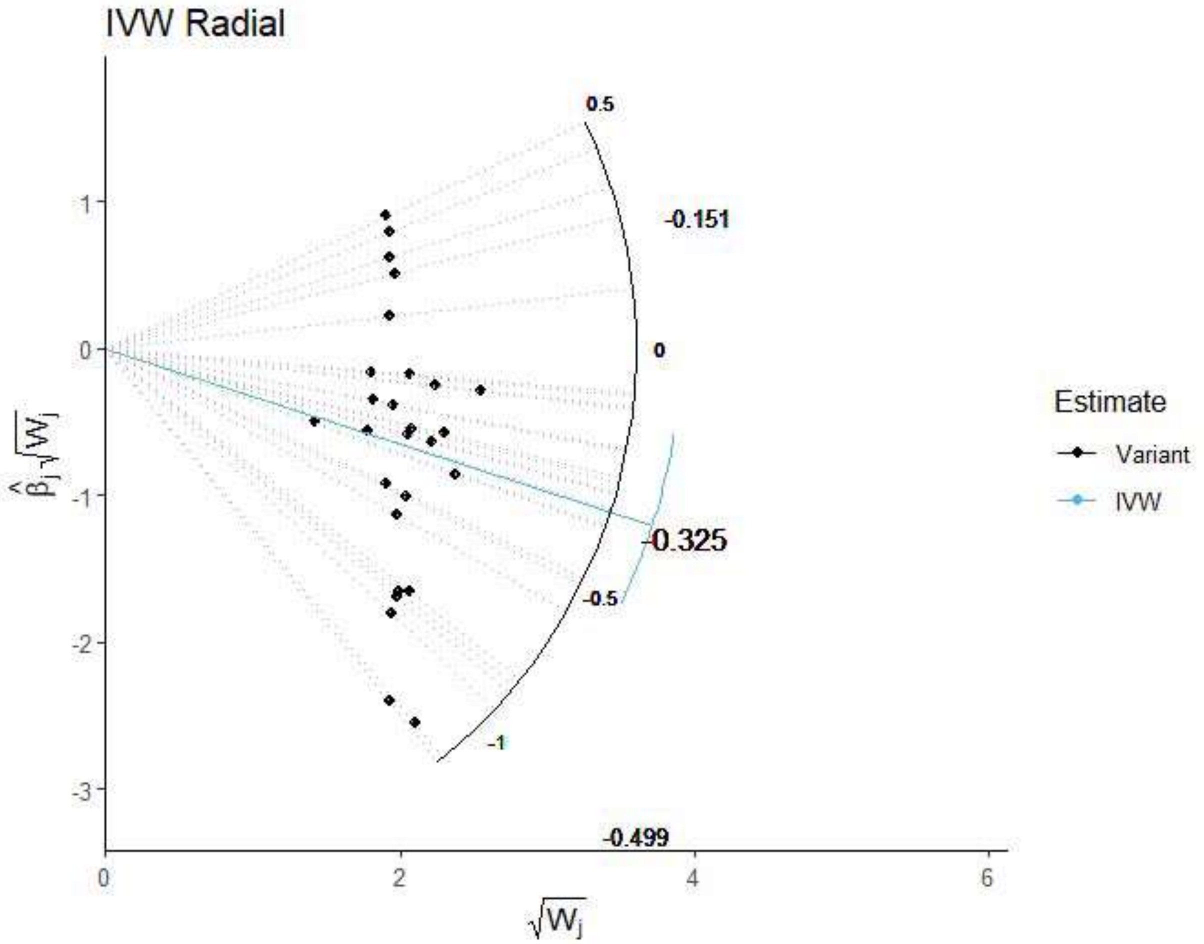

Supplementary figure 2 q

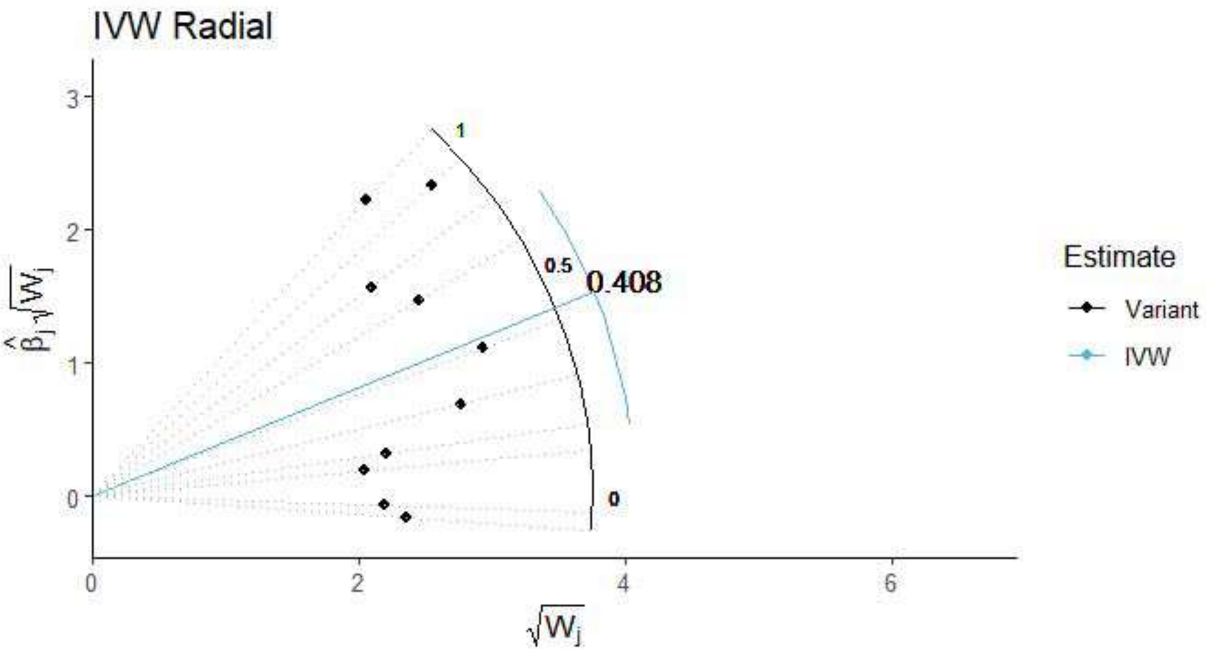

Supplementary figure r

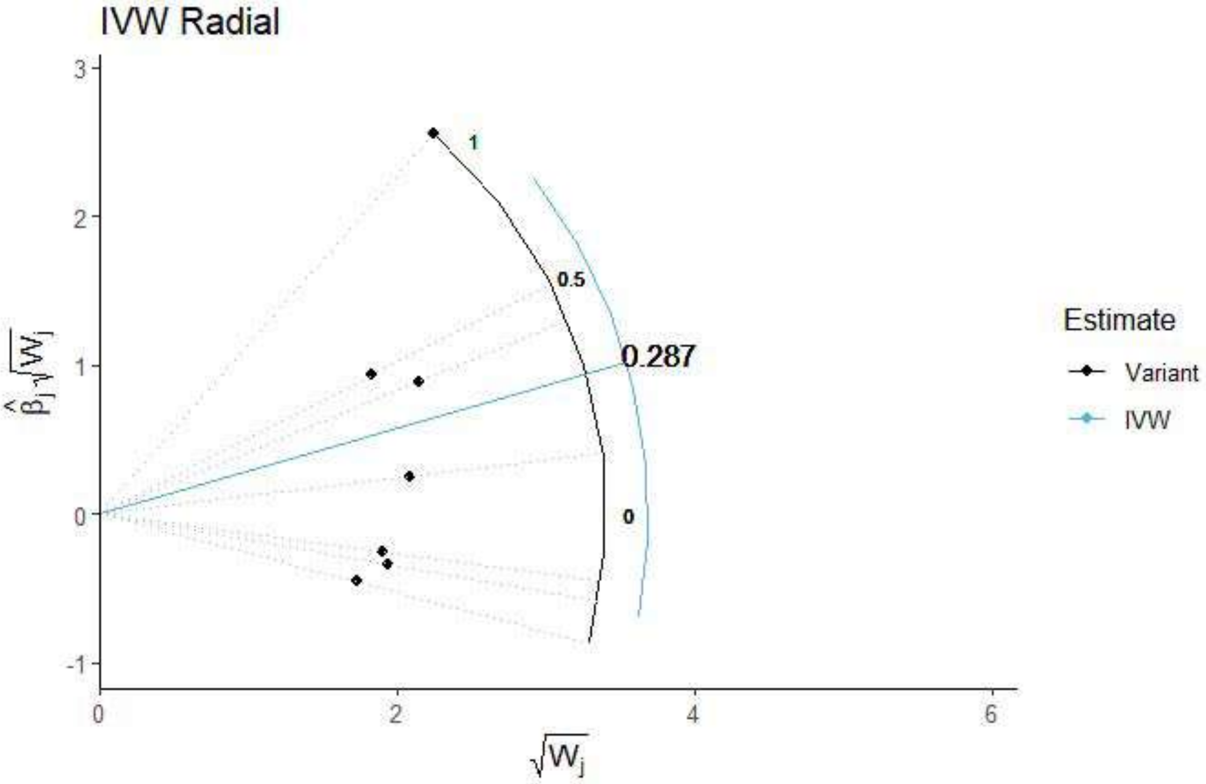

Supplementary figure 2 s

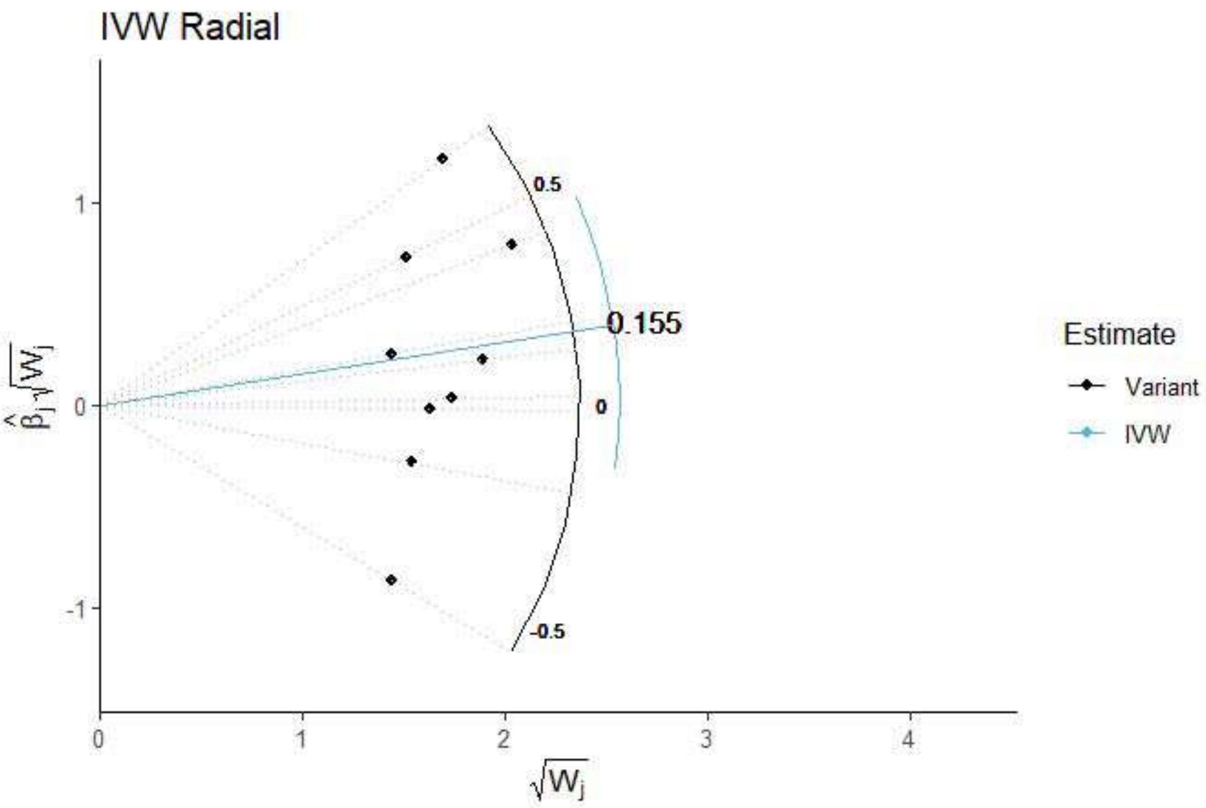

Supplementary figure 2 t

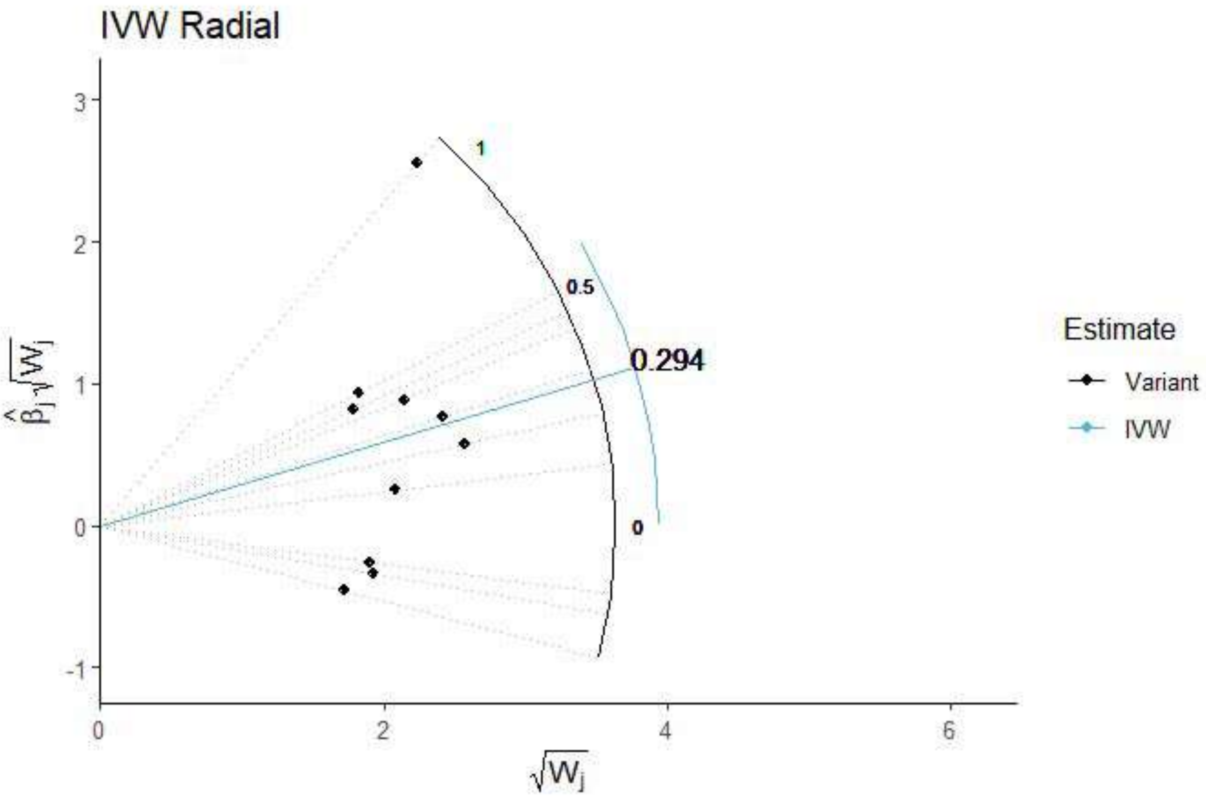

Supplementary figure 3 a

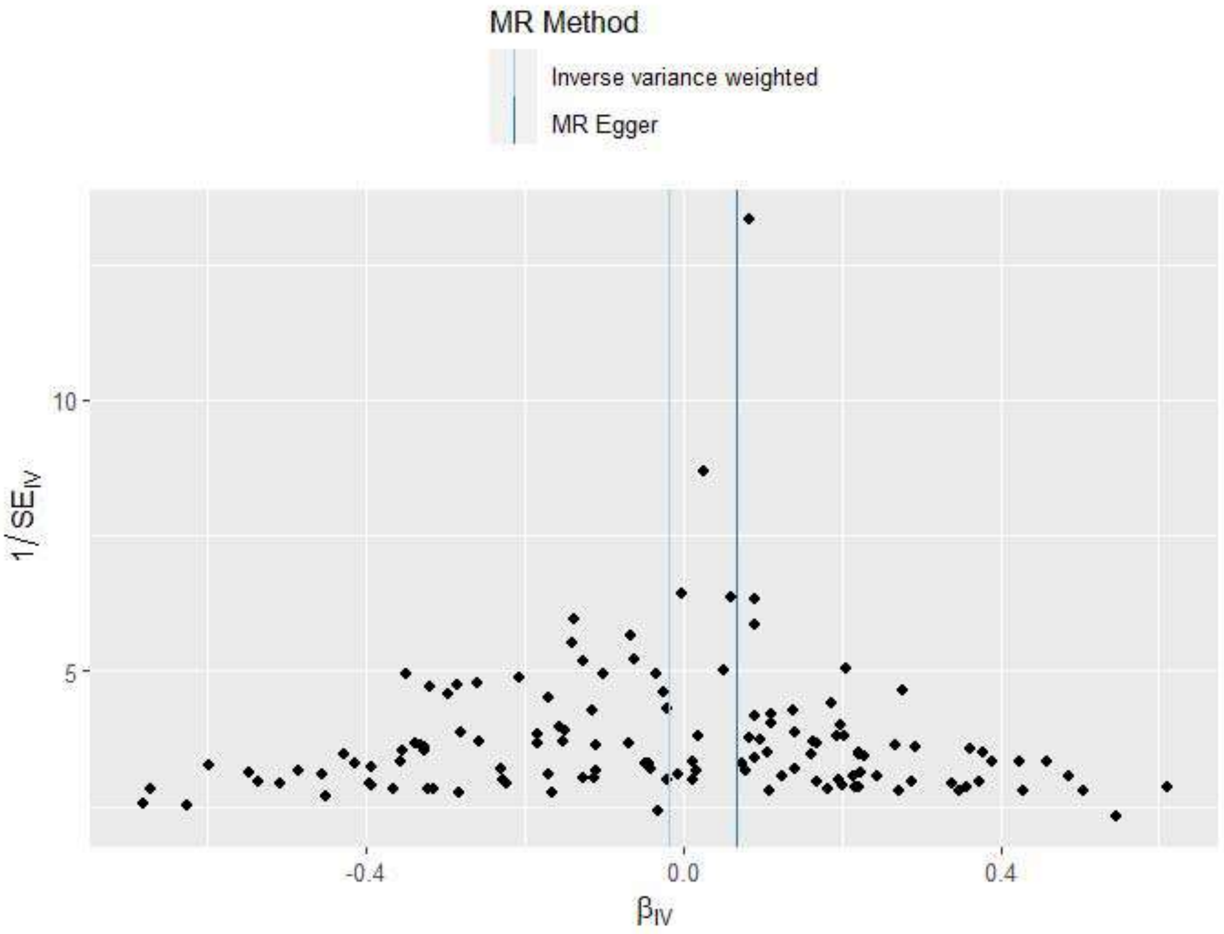

Supplementary figure 3 b

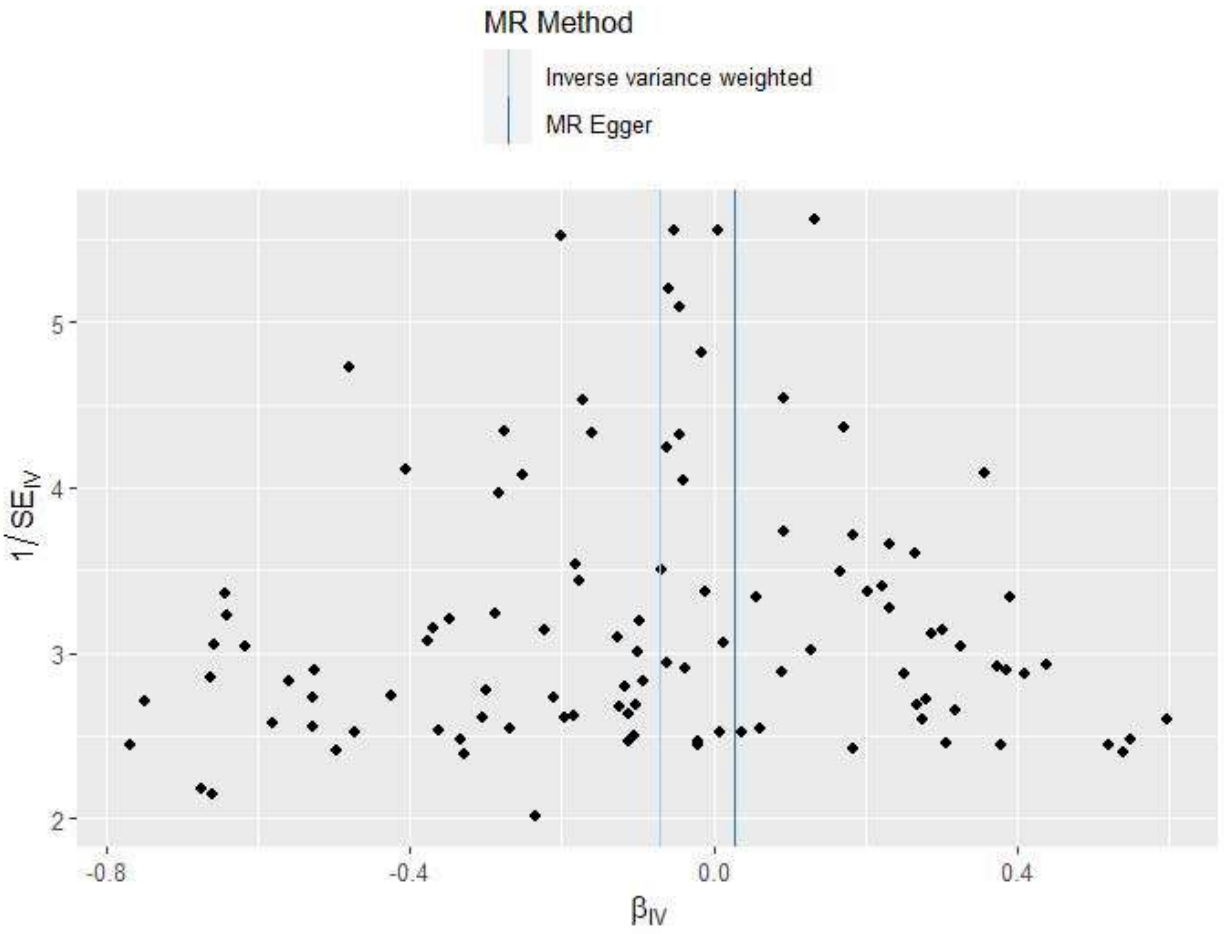

Supplementary figure 3 c

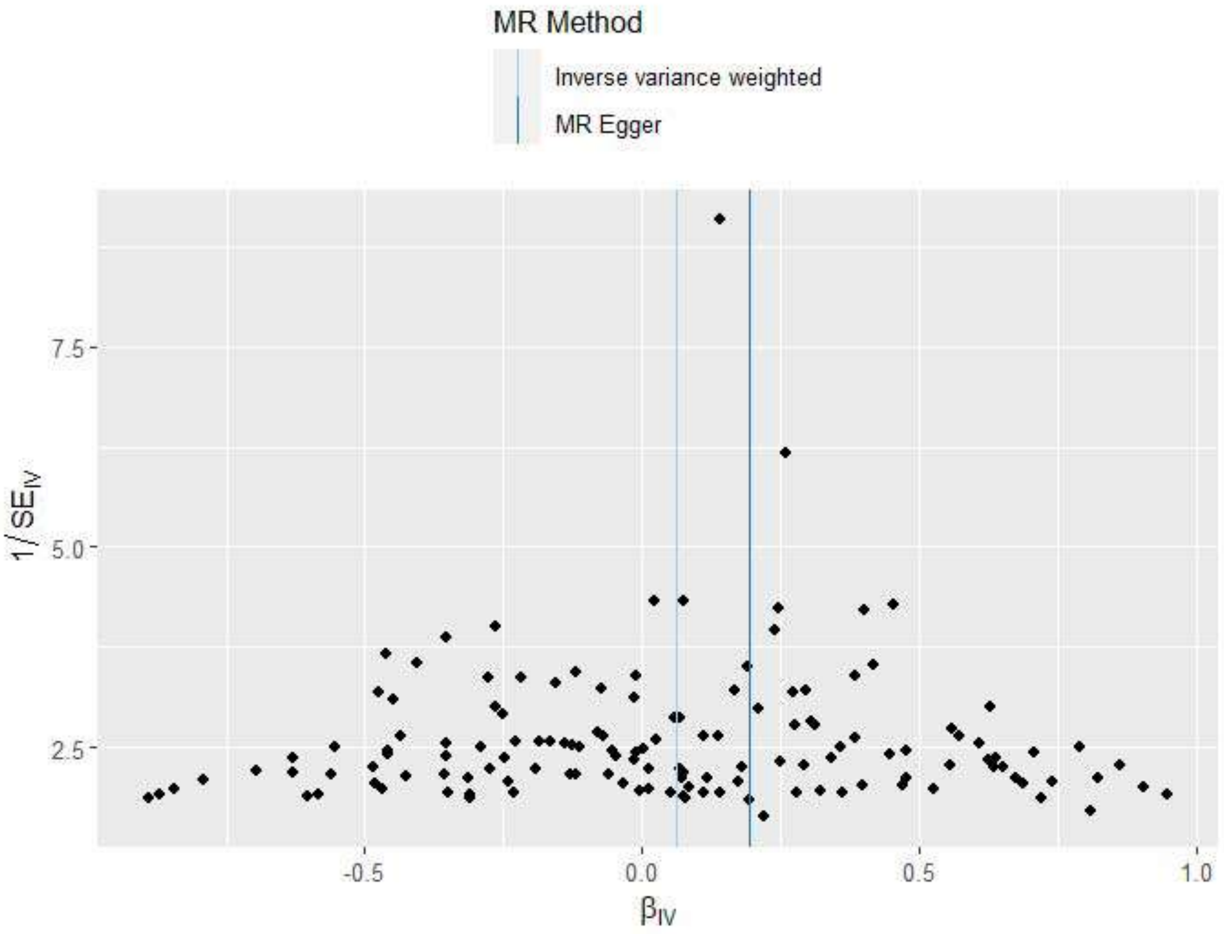

Supplementary figure 3 d

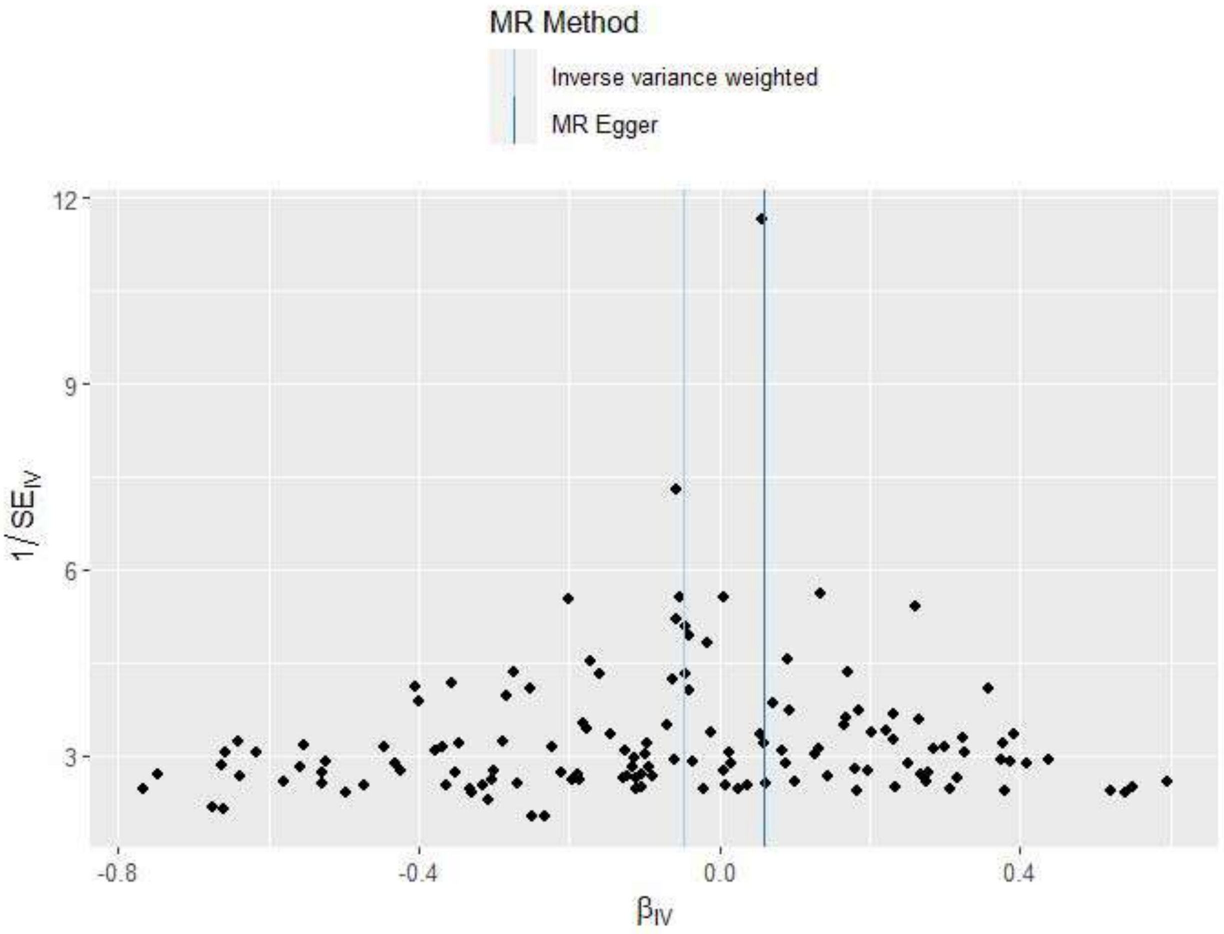

Supplementary figure 3 e

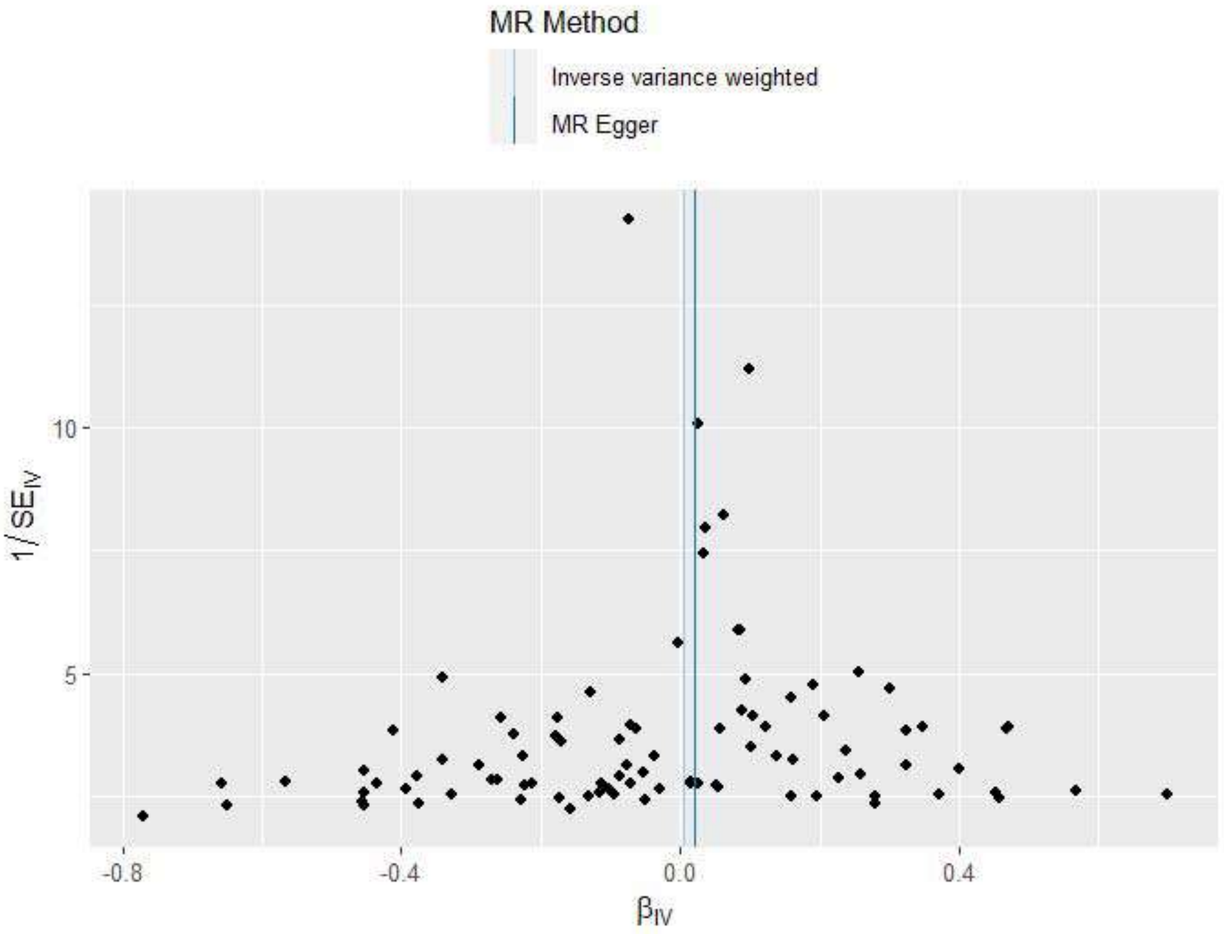

Supplementary figure 3 f

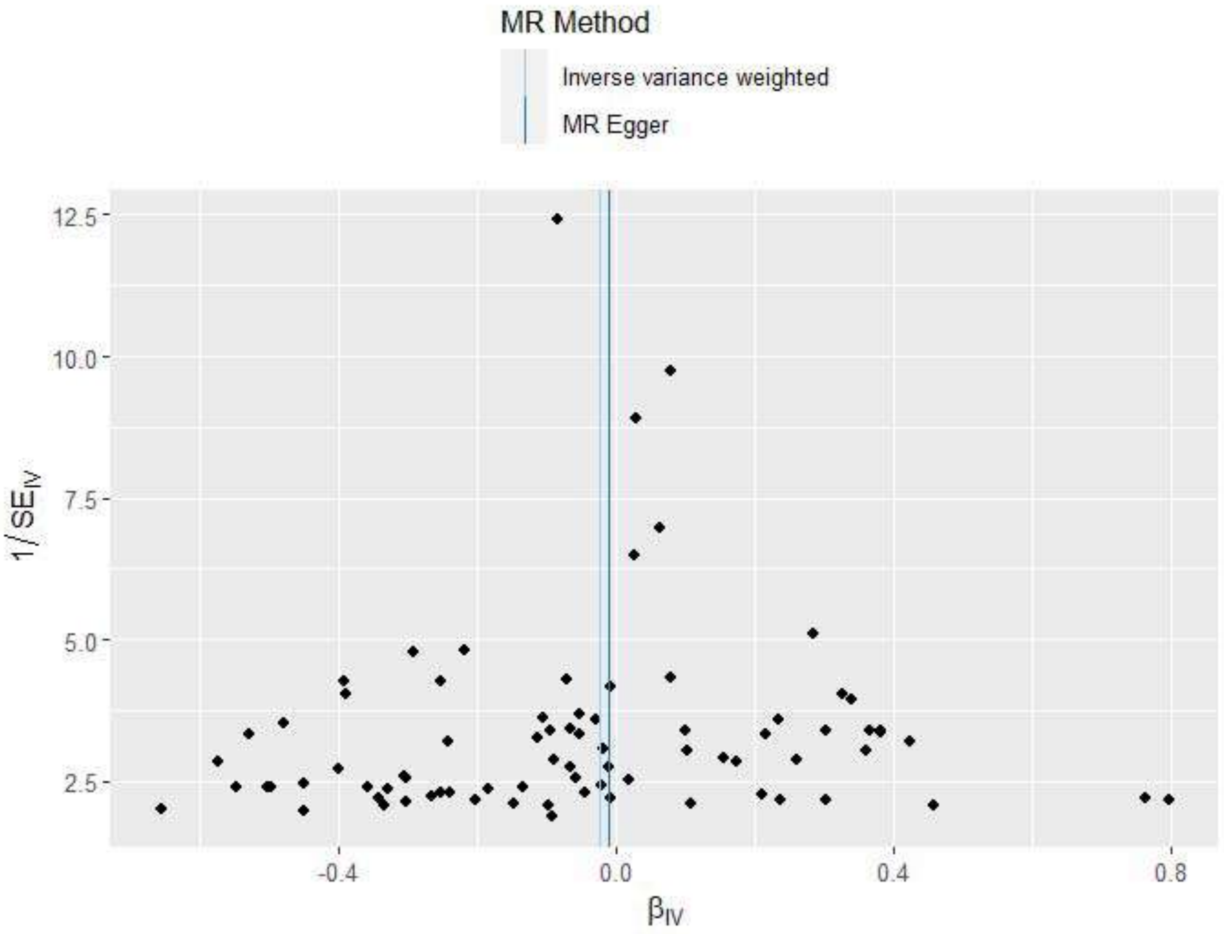

Supplementary figure 3 g

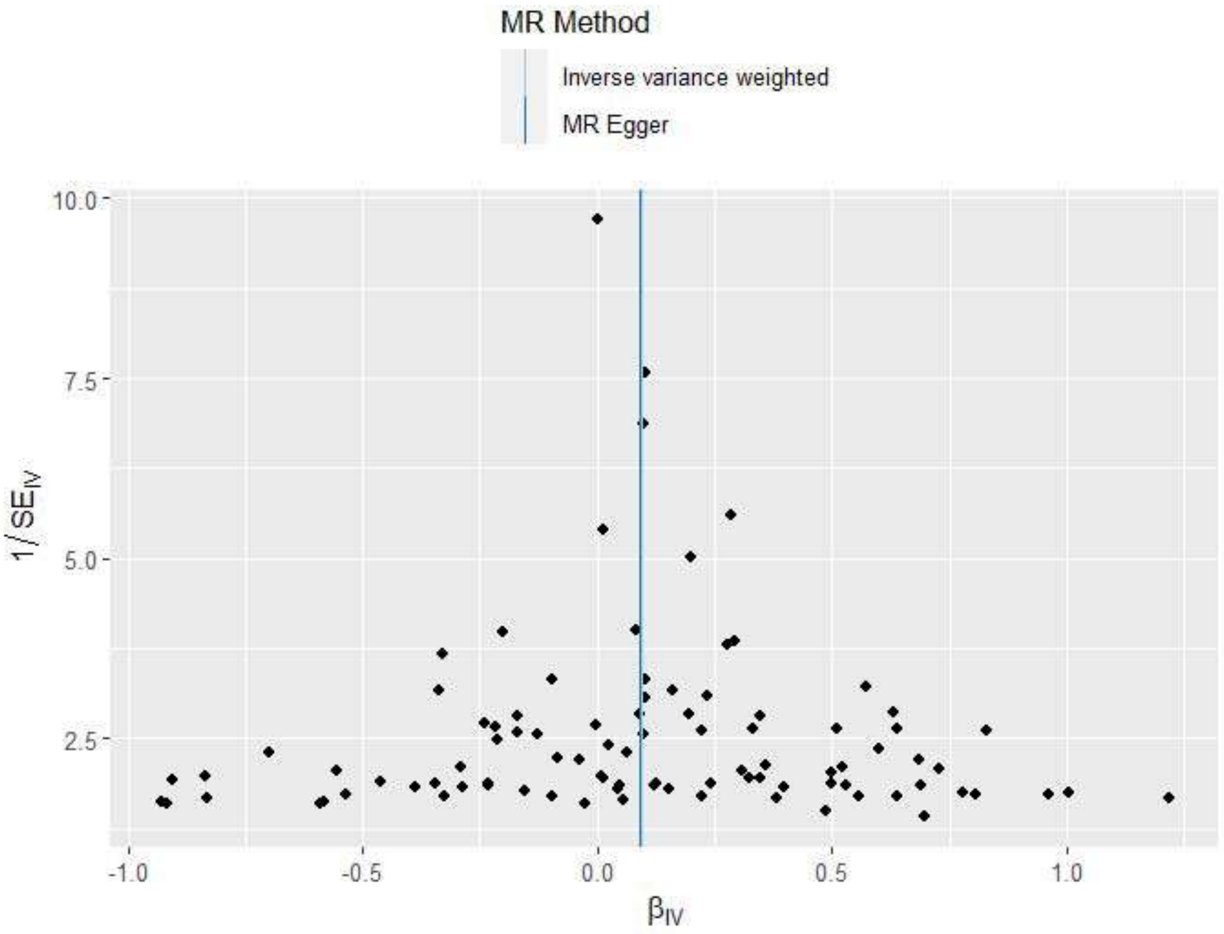

Supplementary figure 3 h

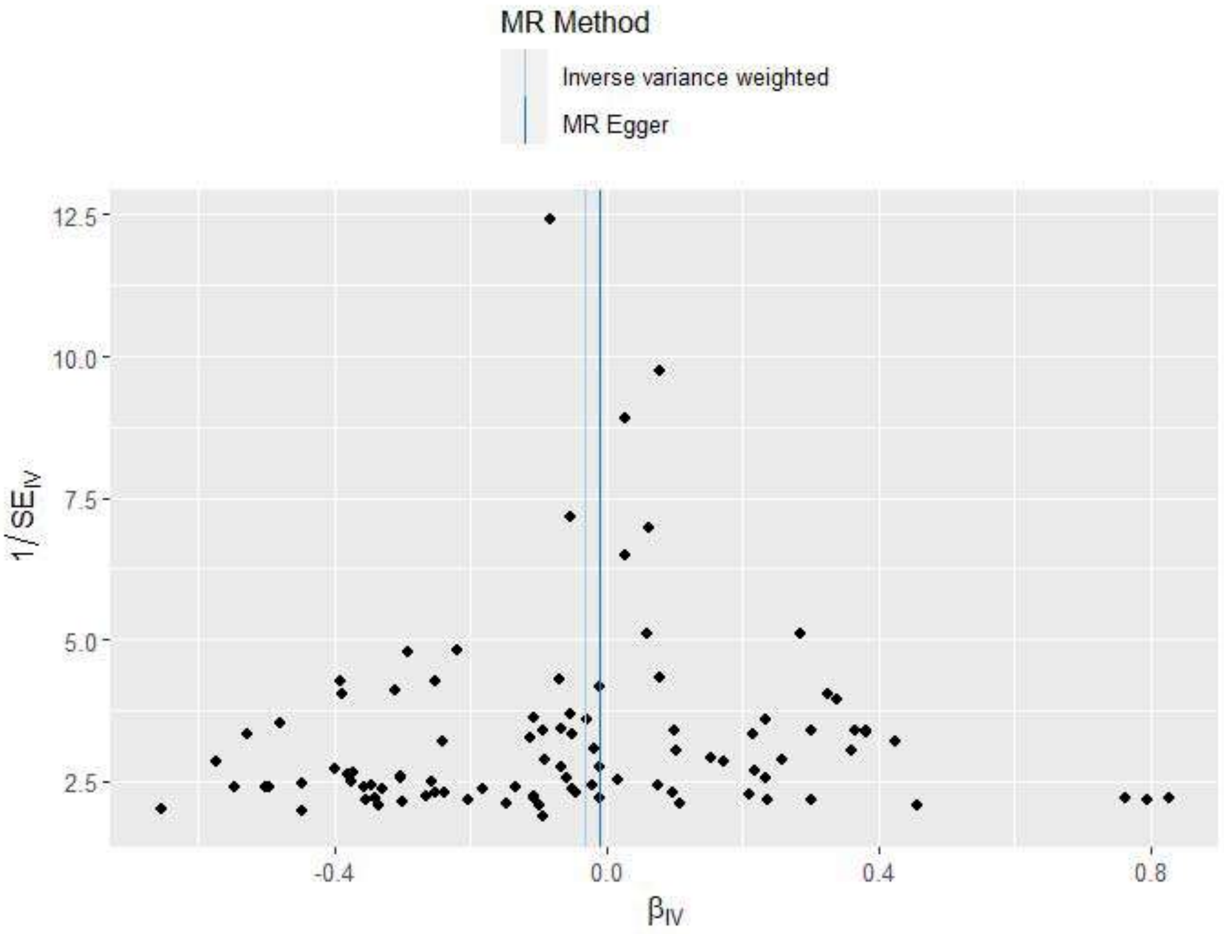

Supplementary figure 3 I

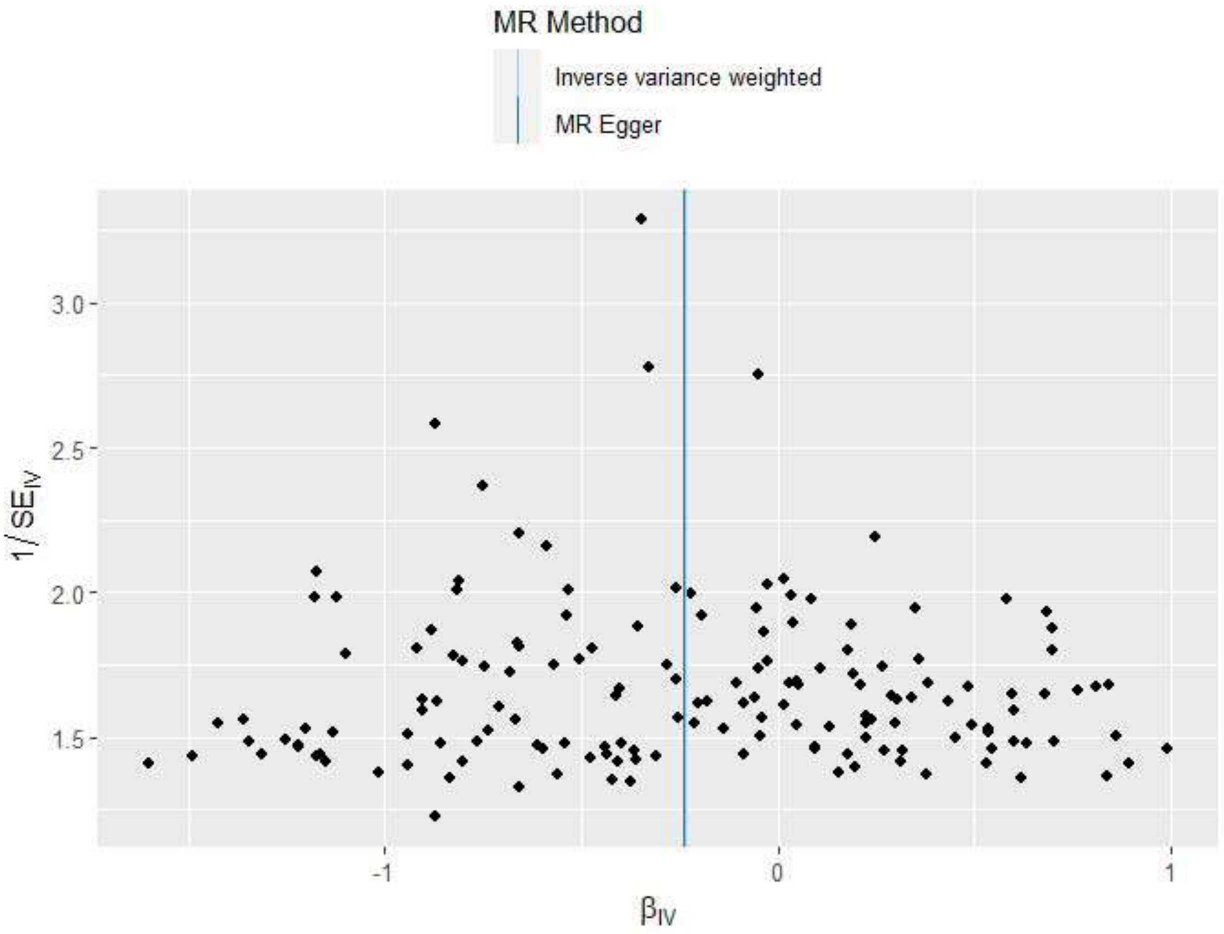

Supplementary figure j

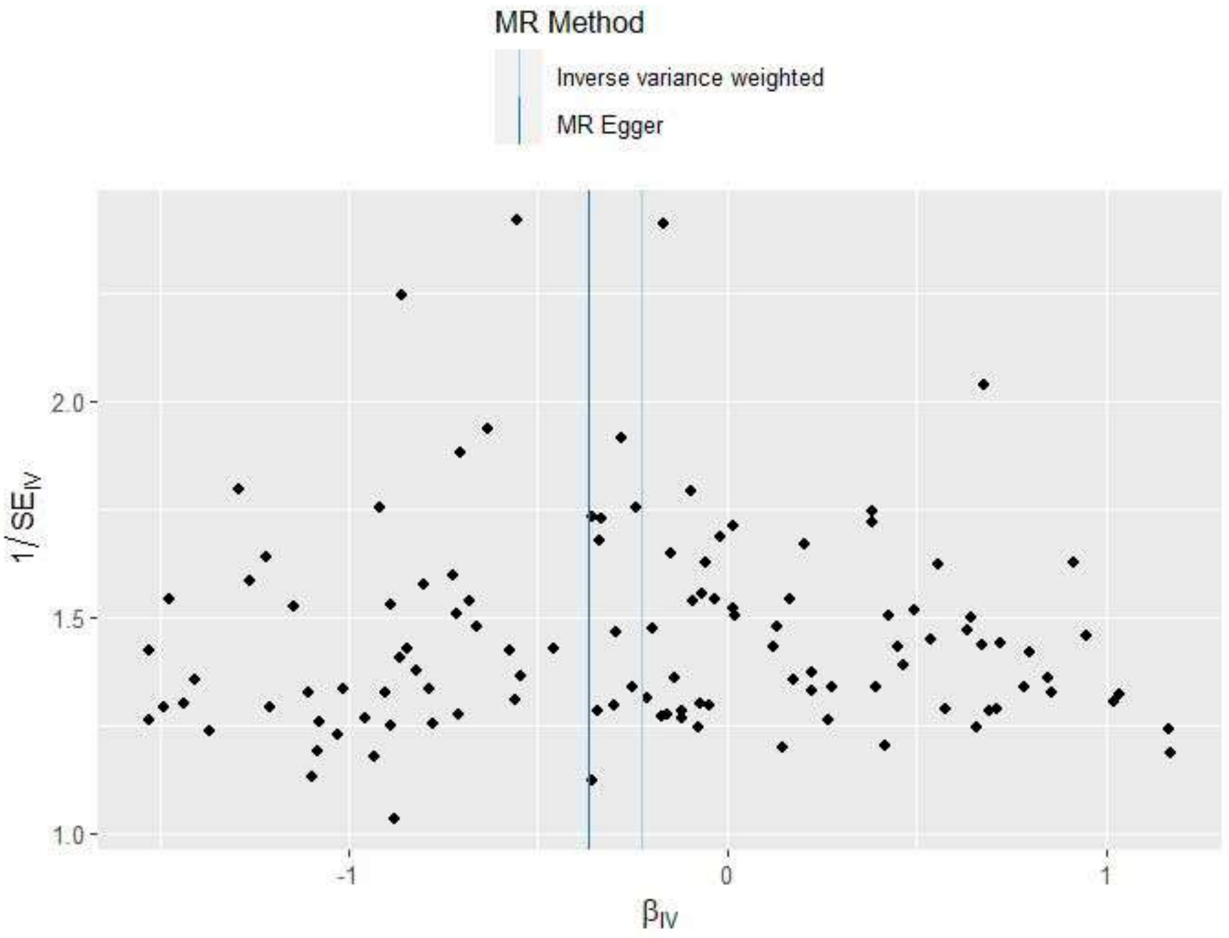

Supplementary figure 3 k

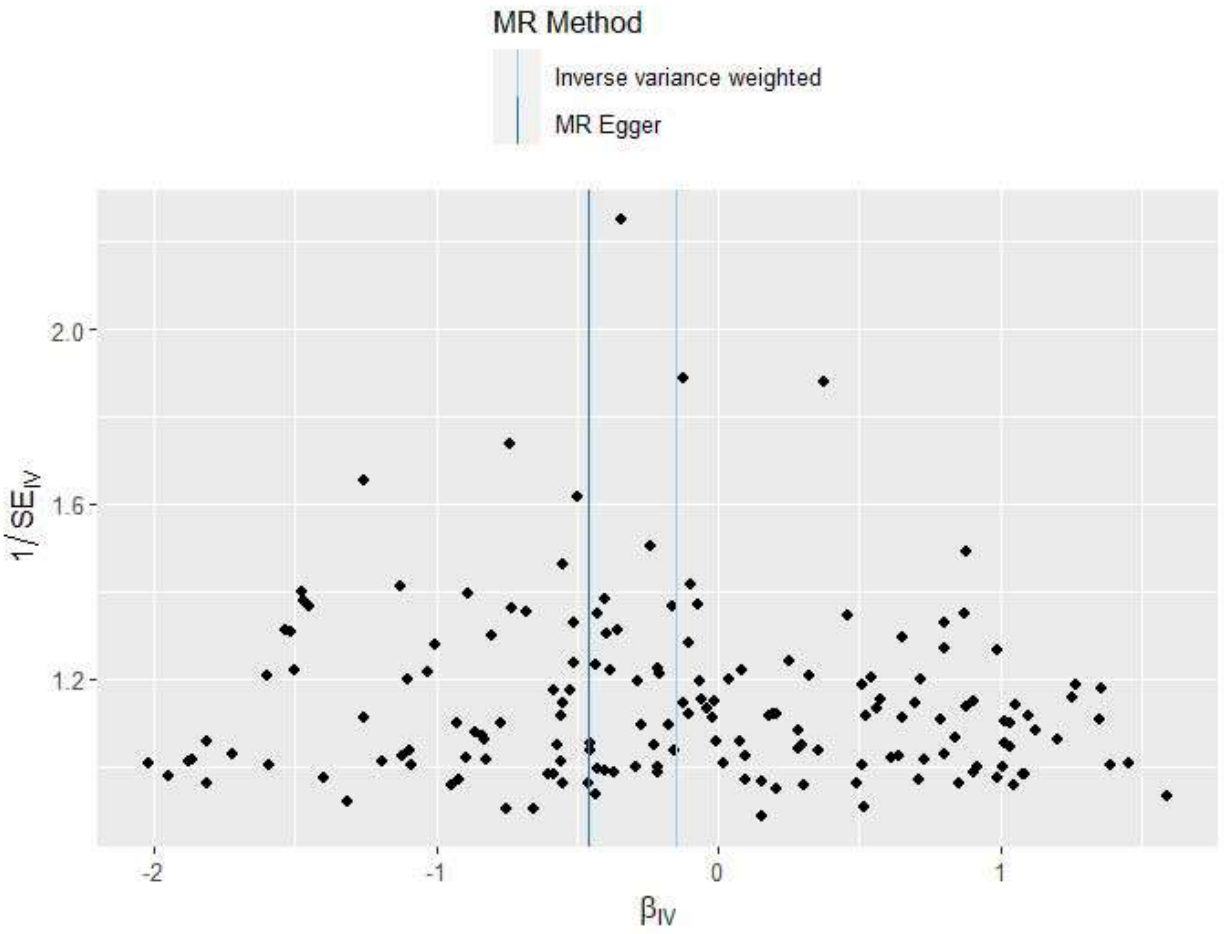

Supplementary figure 3 l

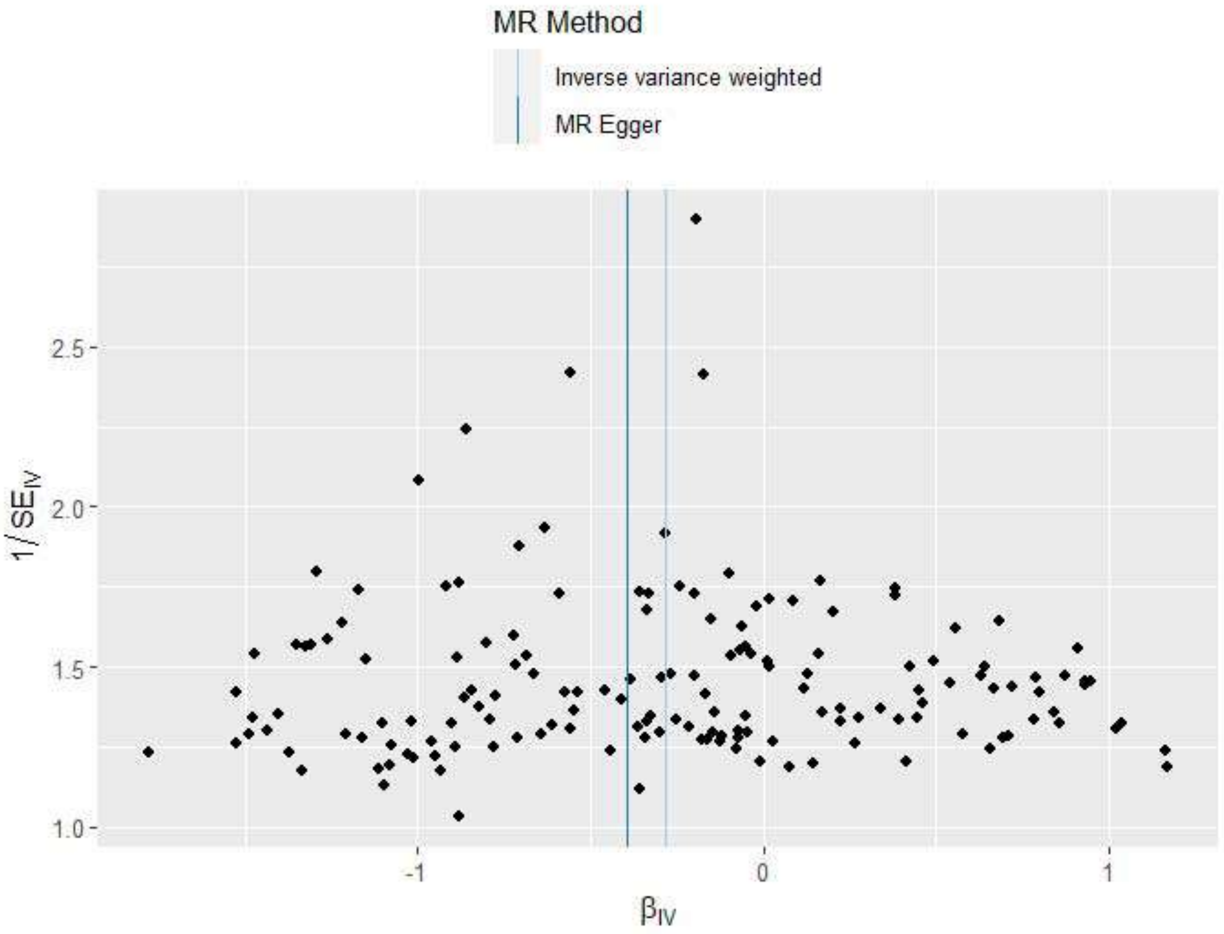

Supplementary figure 3 m

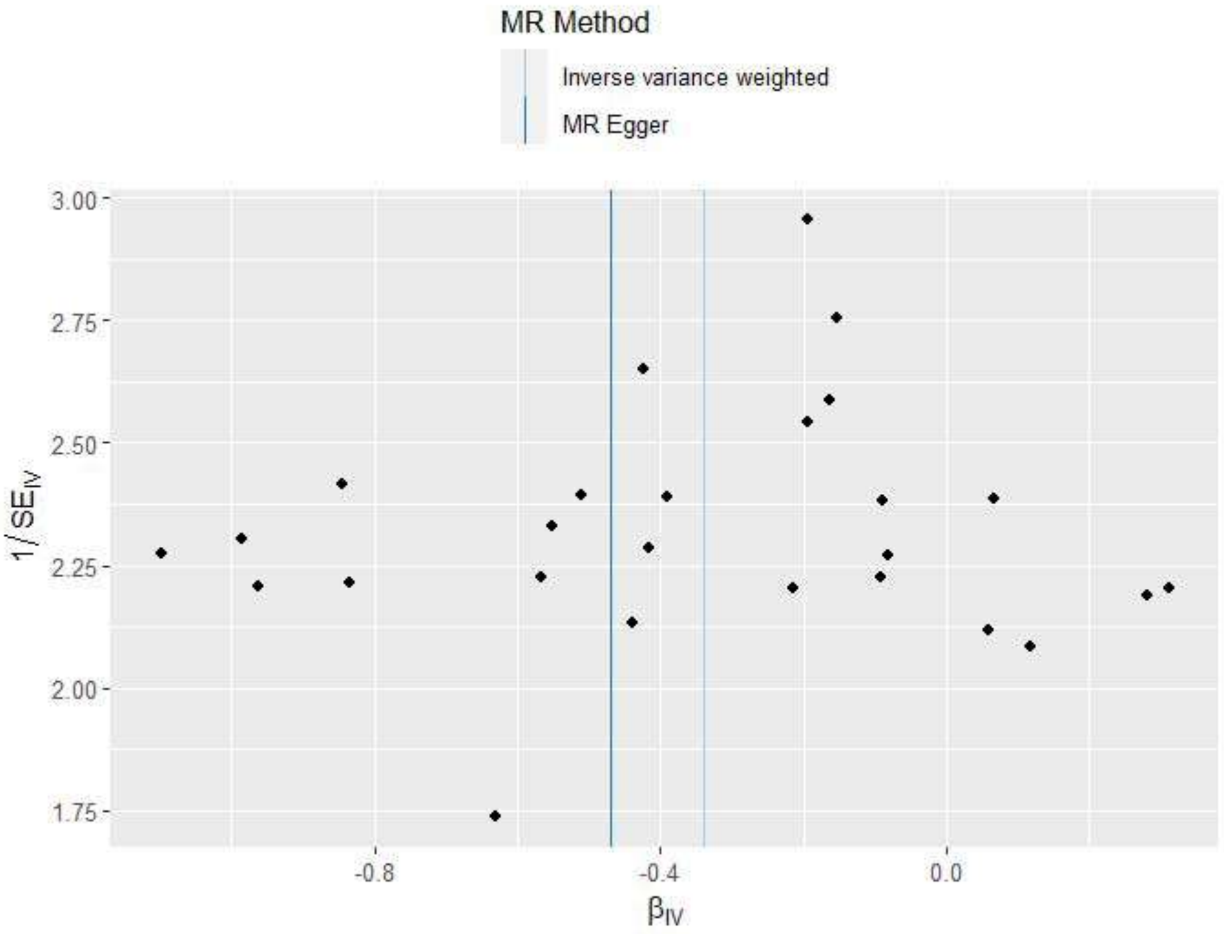

Supplementary figure 3 n

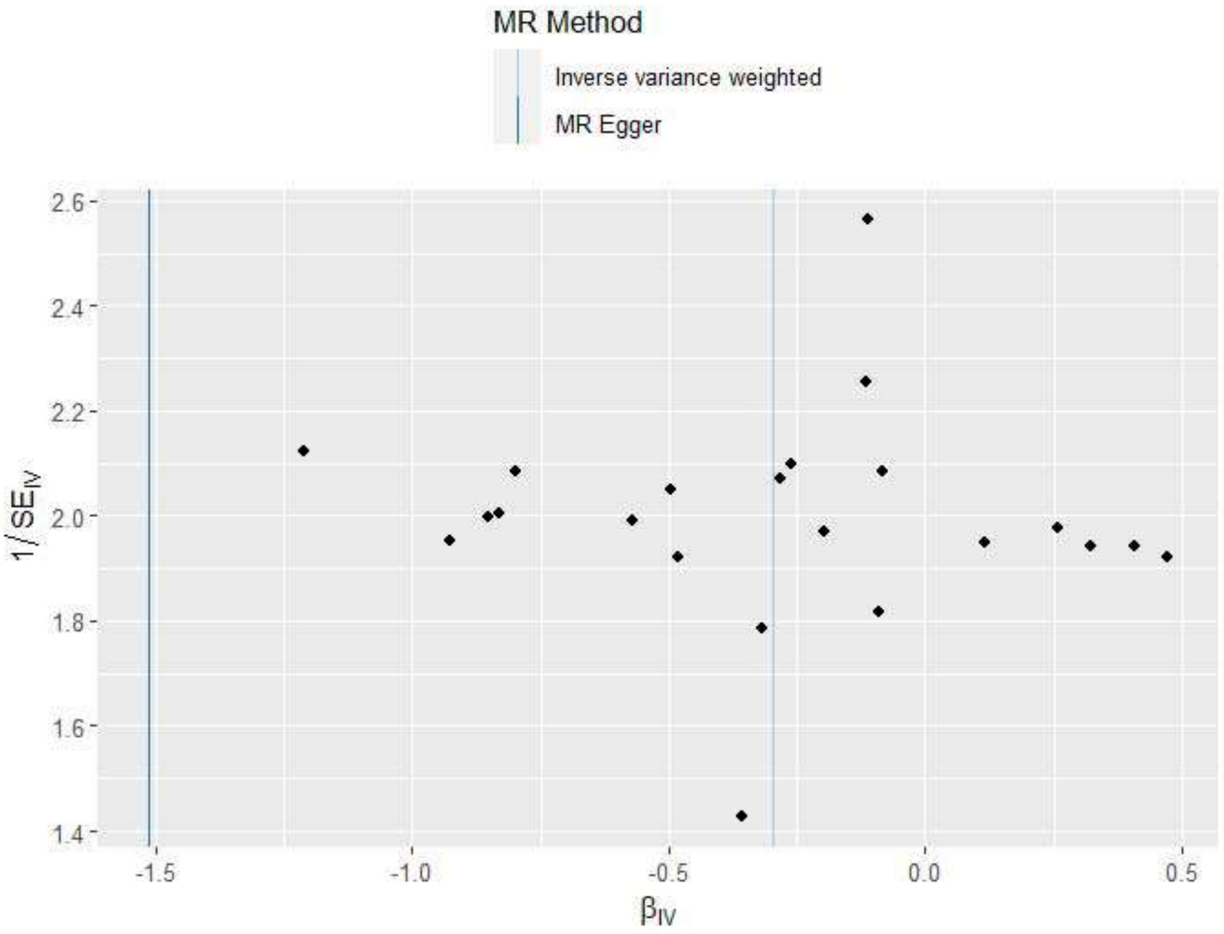

Supplementary figure 3 o

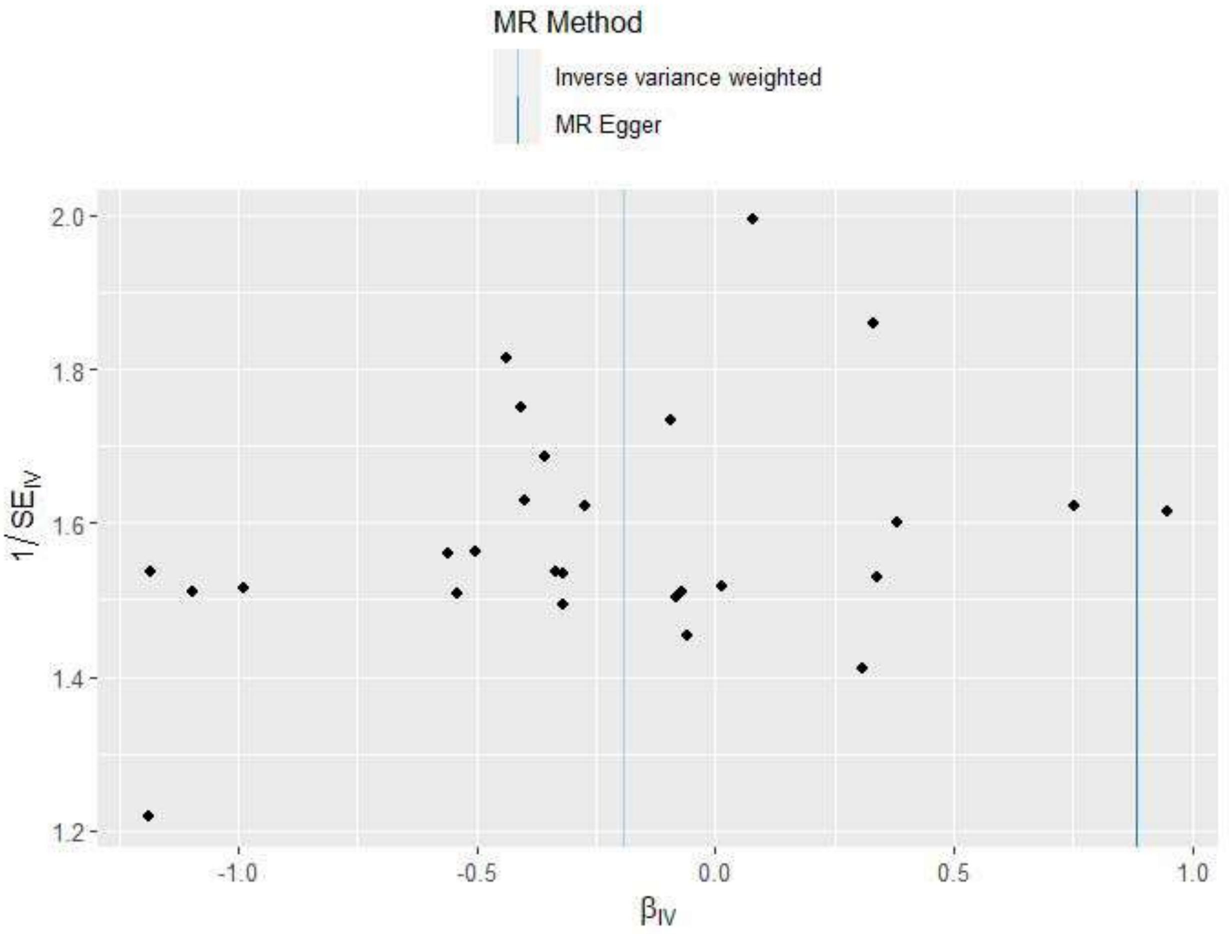

Supplementary figure 3 p

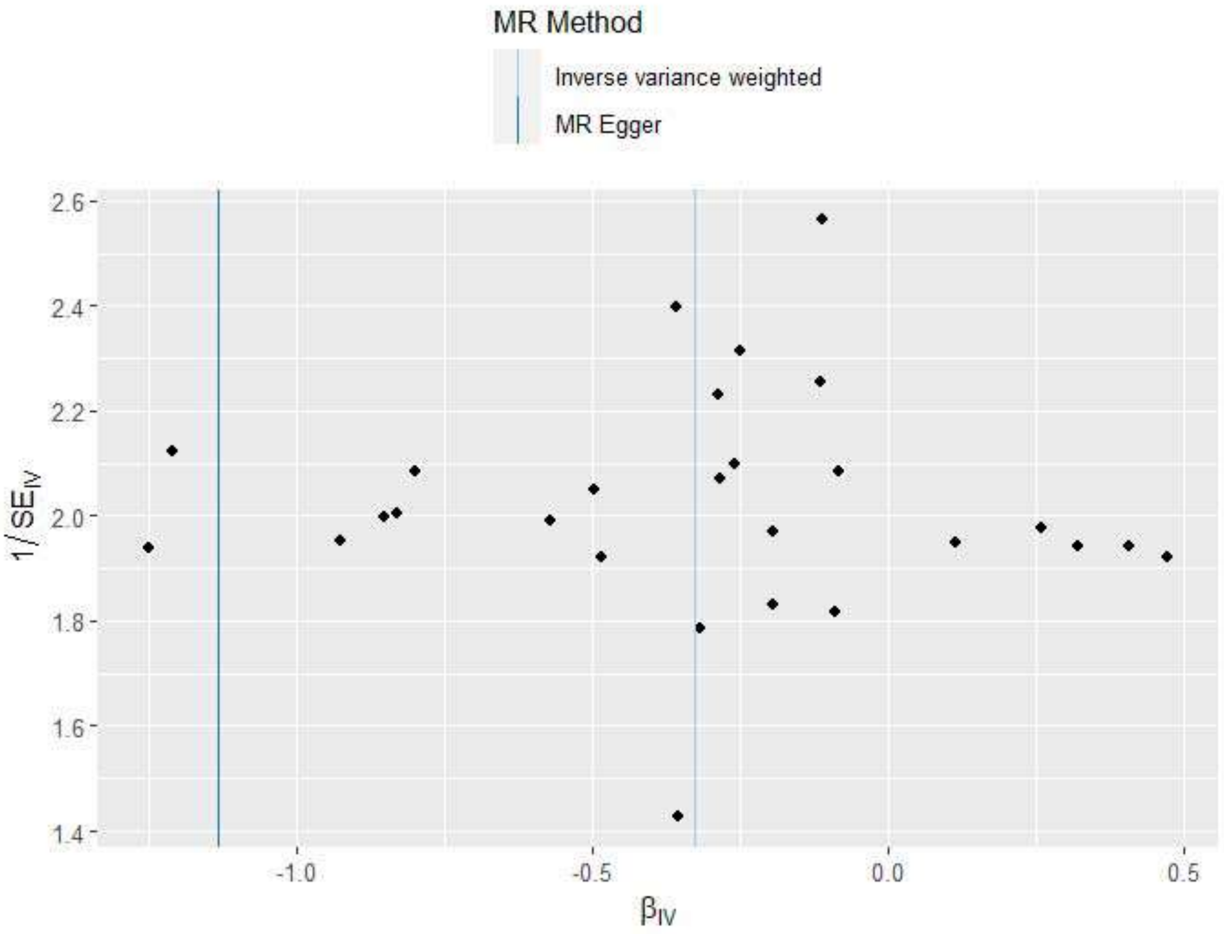

Supplementary figure 3 q

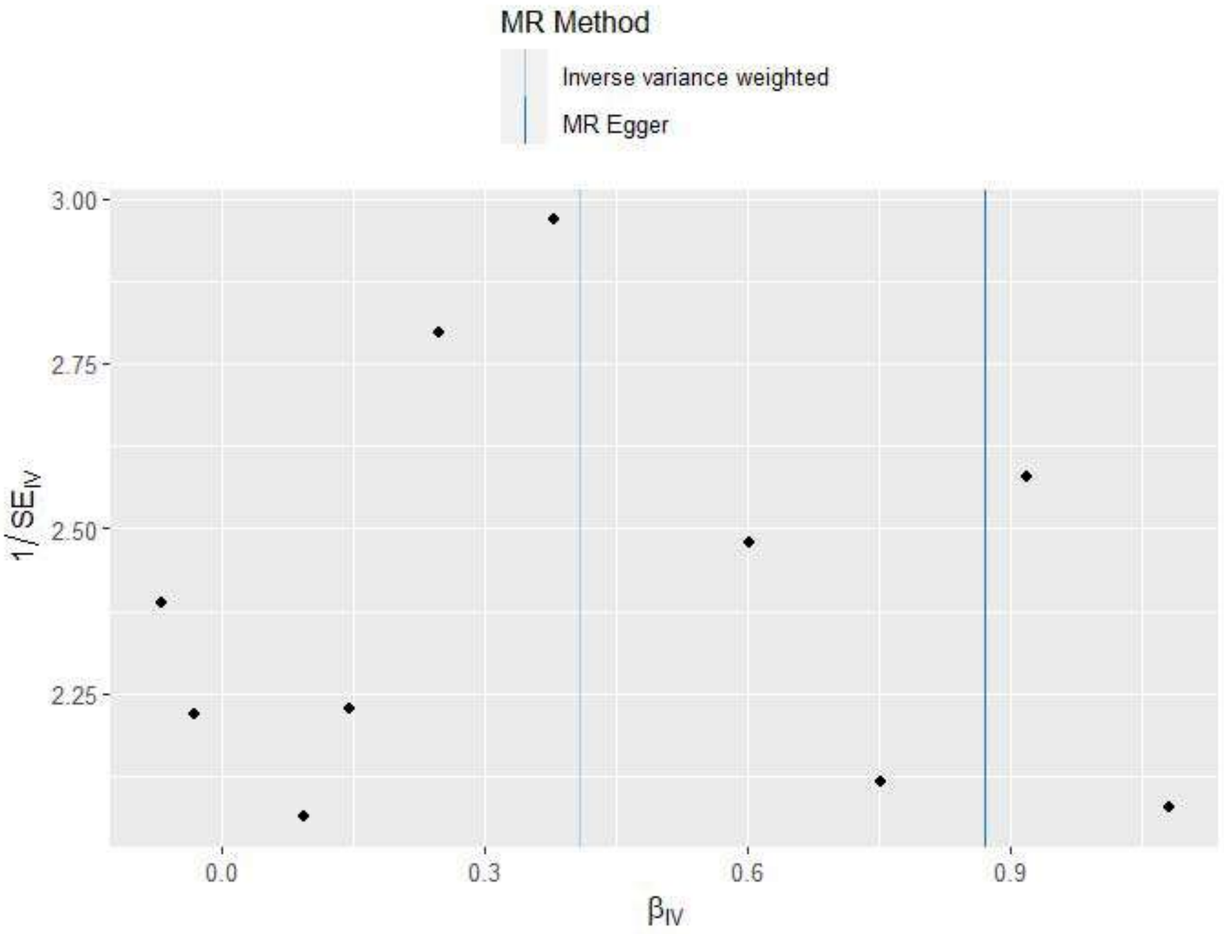

Supplementary figure 3 r

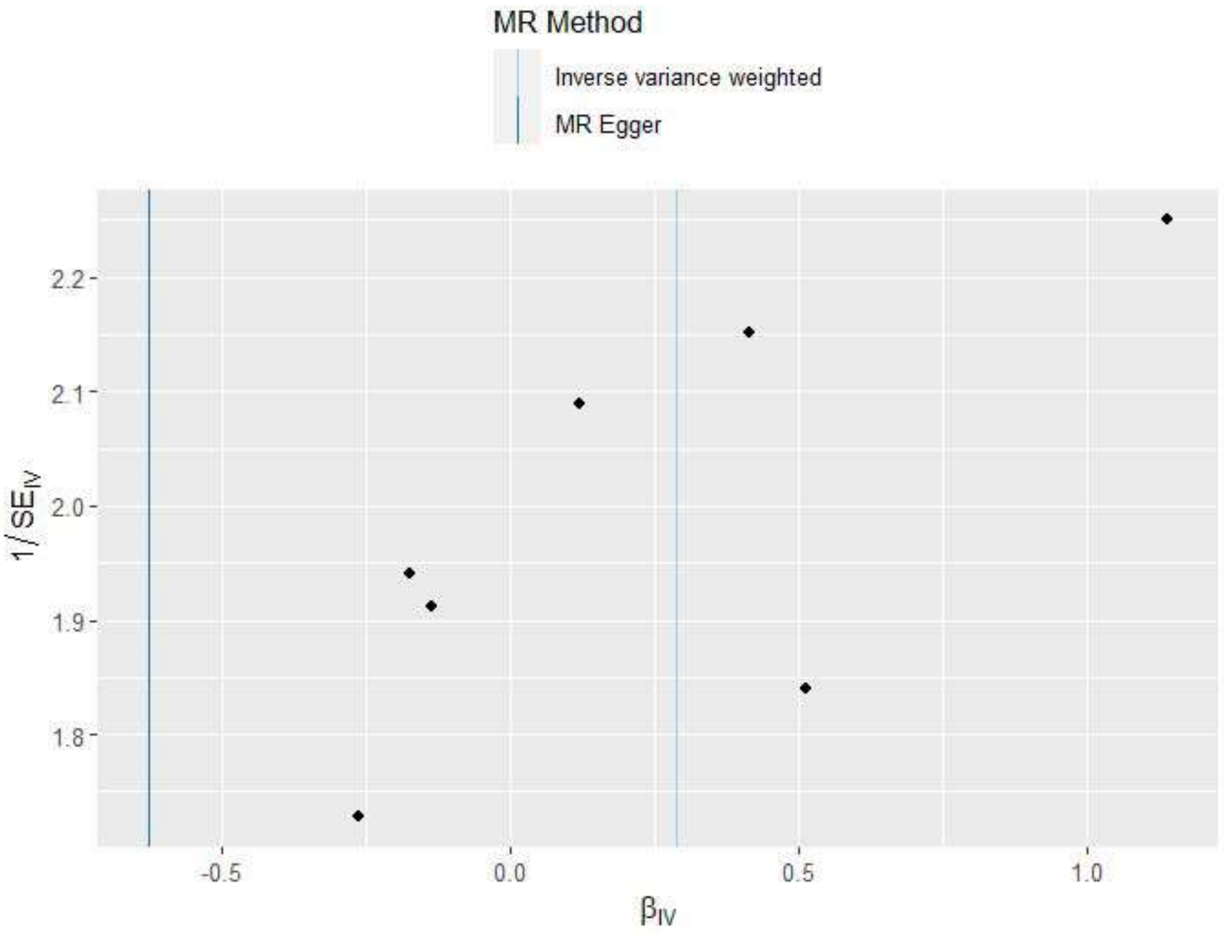

Supplementary figure 3 s

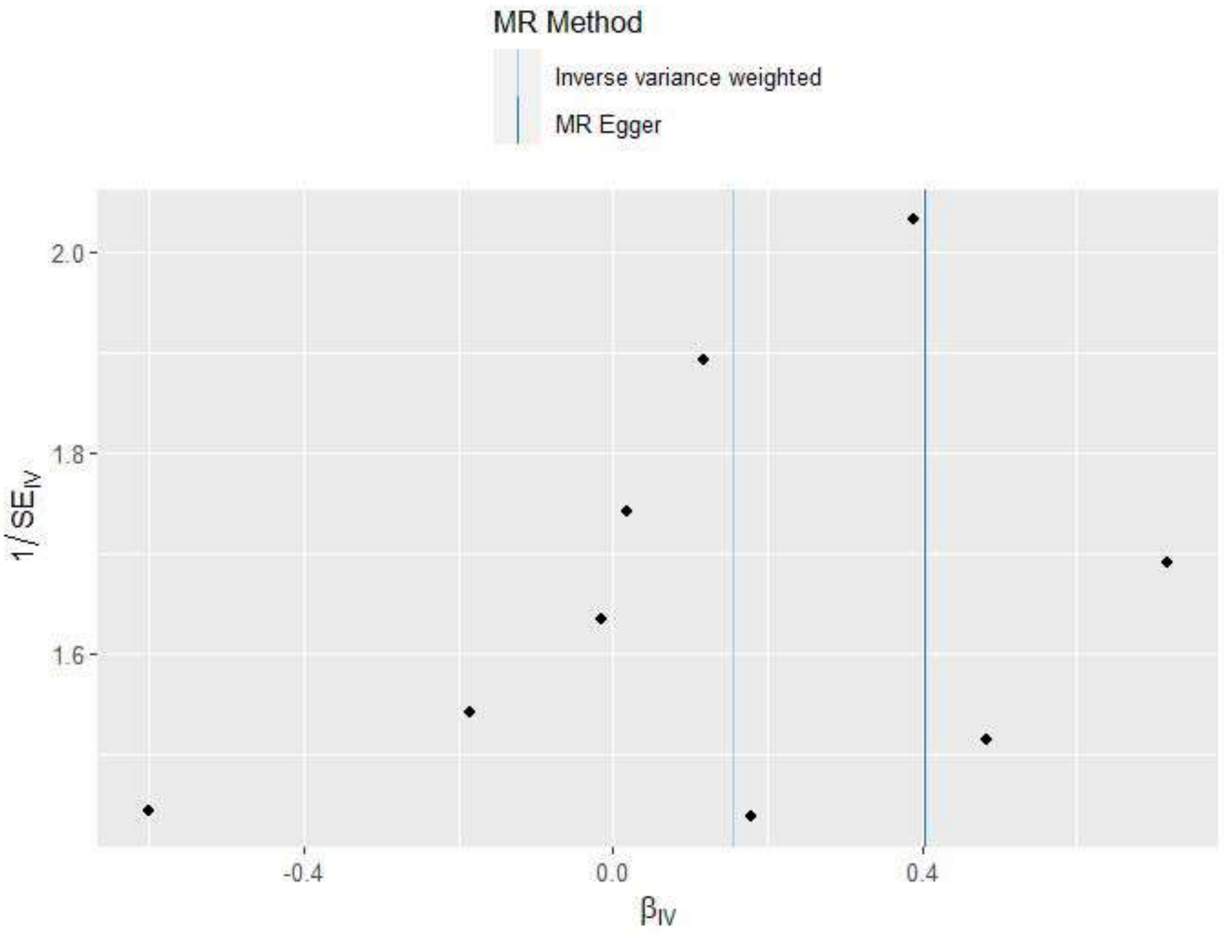

Supplementary figure 3 t

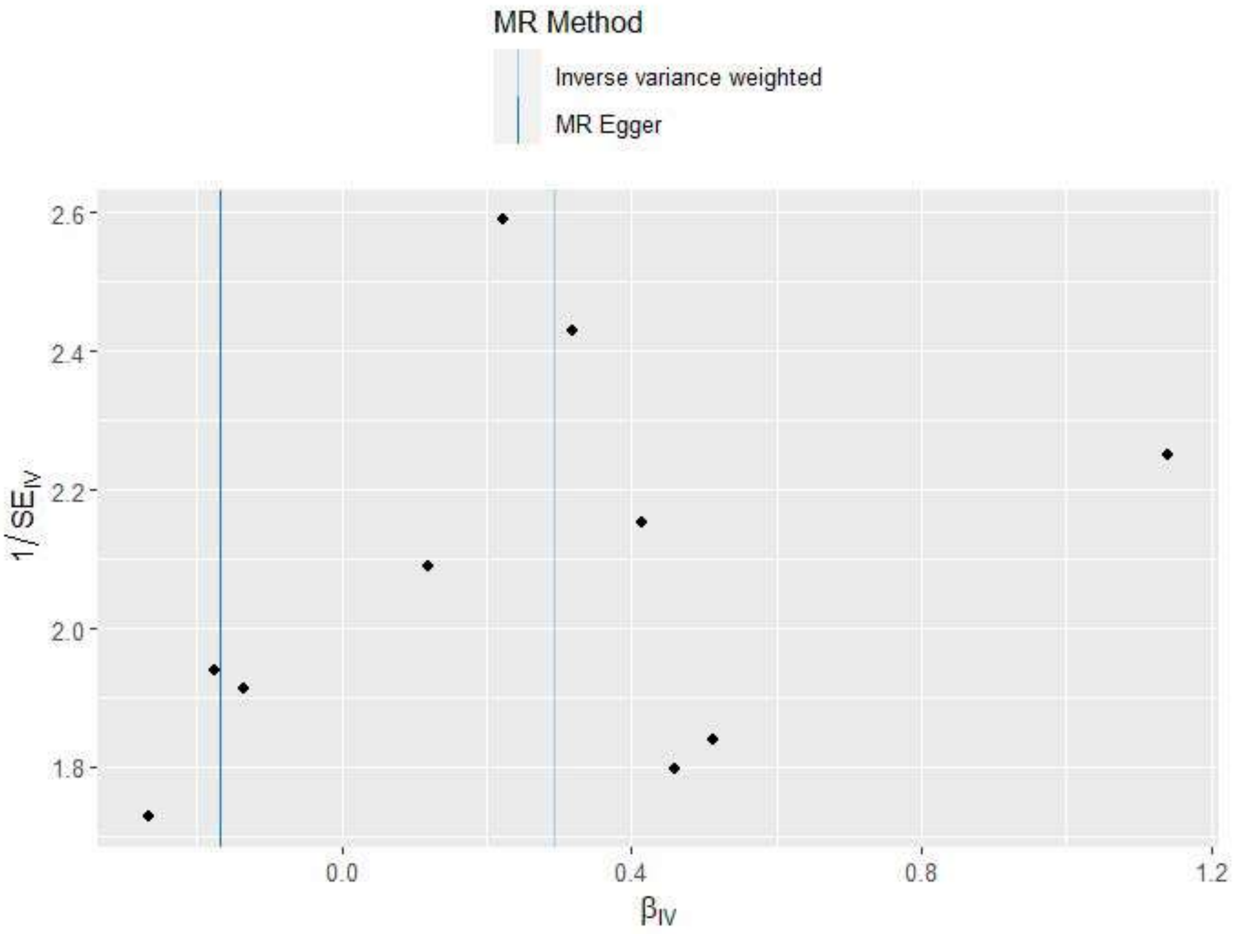

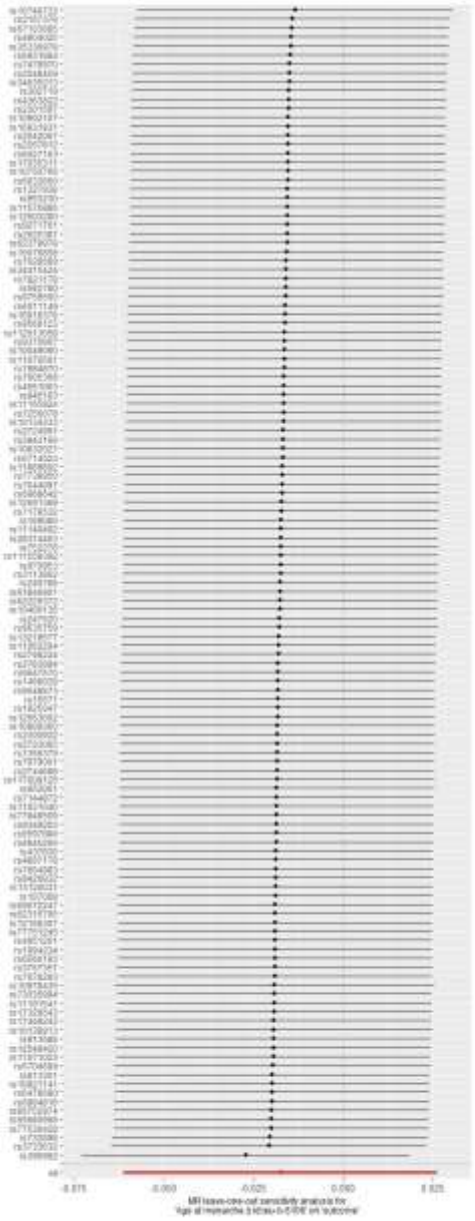

Supplementary figure 4 a

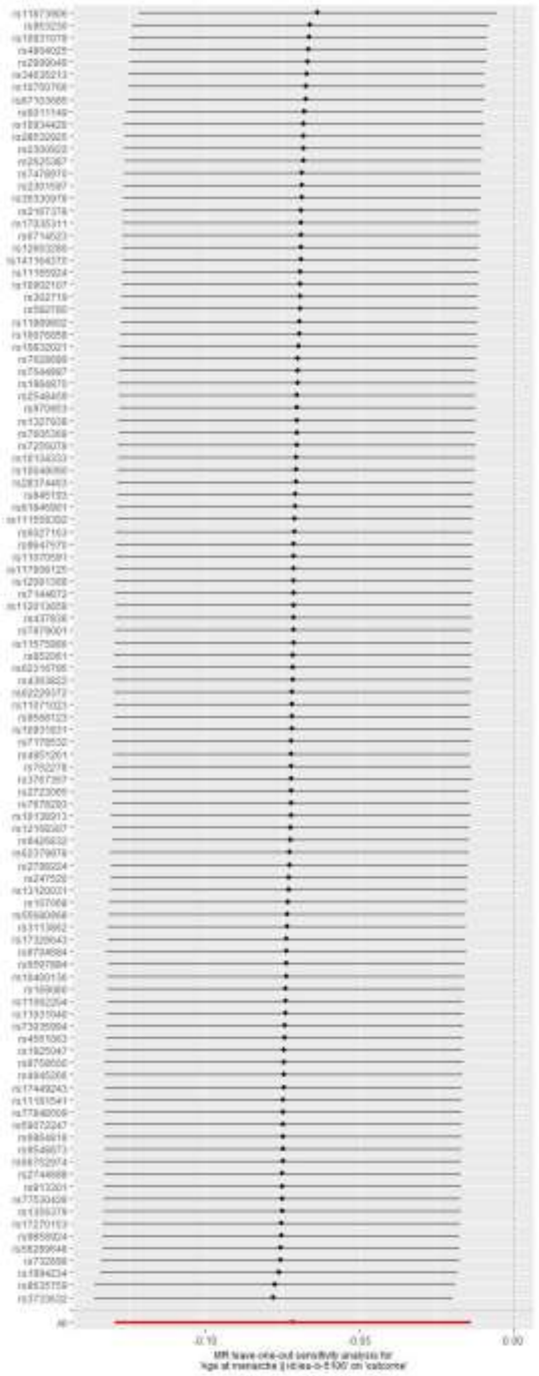

Supplementary figure 4 b

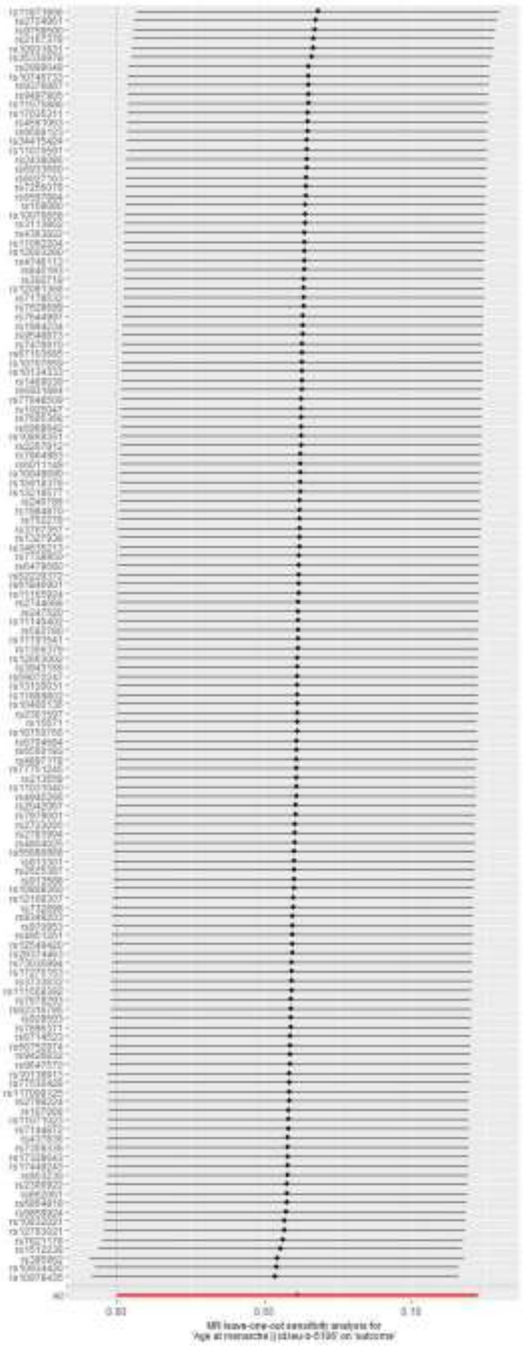

Supplementary figure 4 c

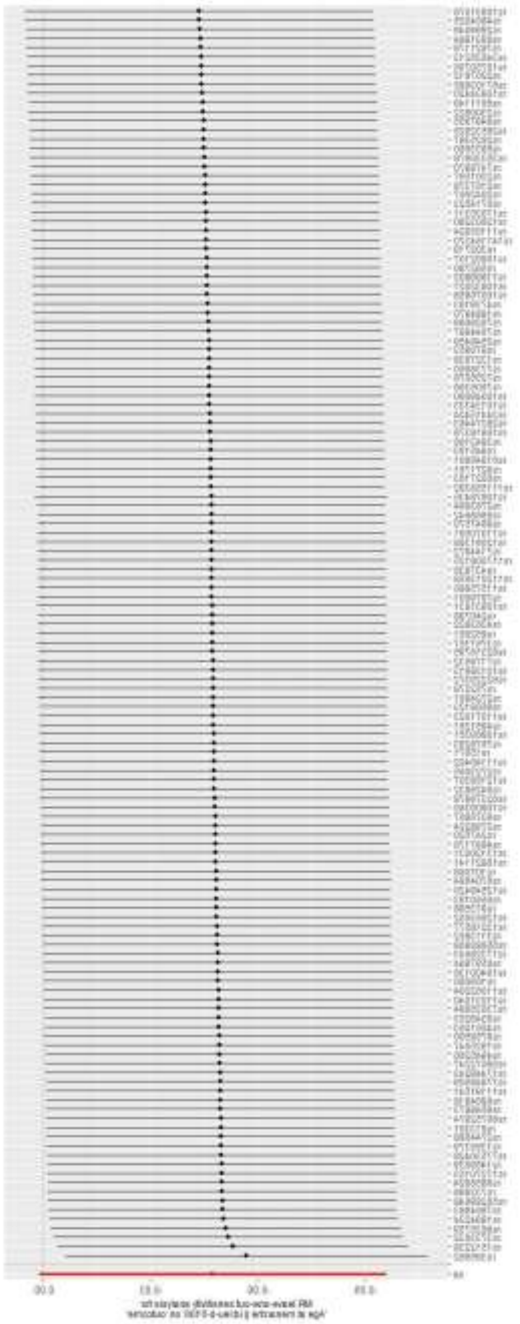

Supplementary figure 4 d

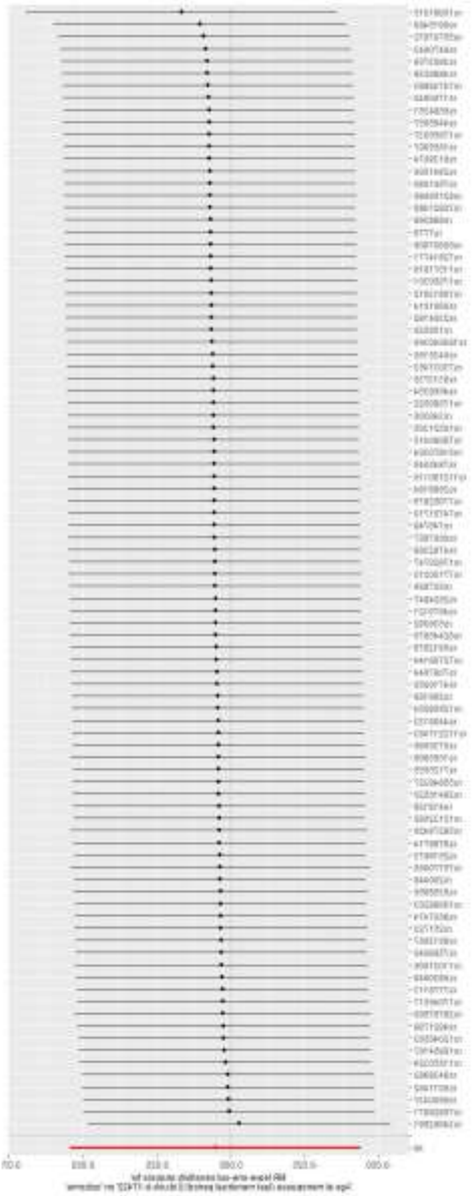

Supplementary figure 4 e

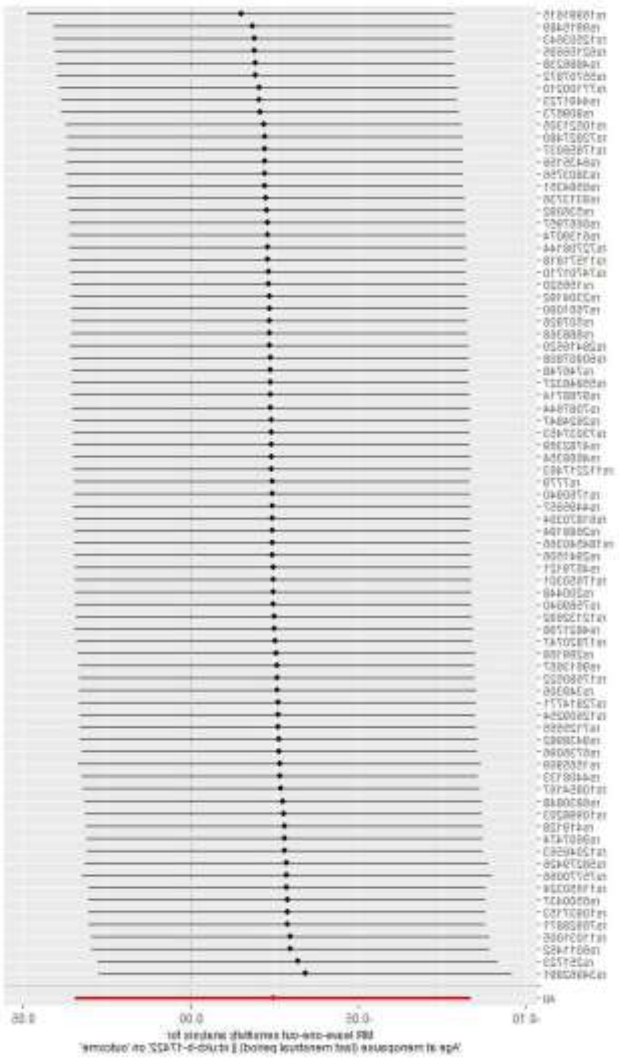

Supplementary figure 4 f

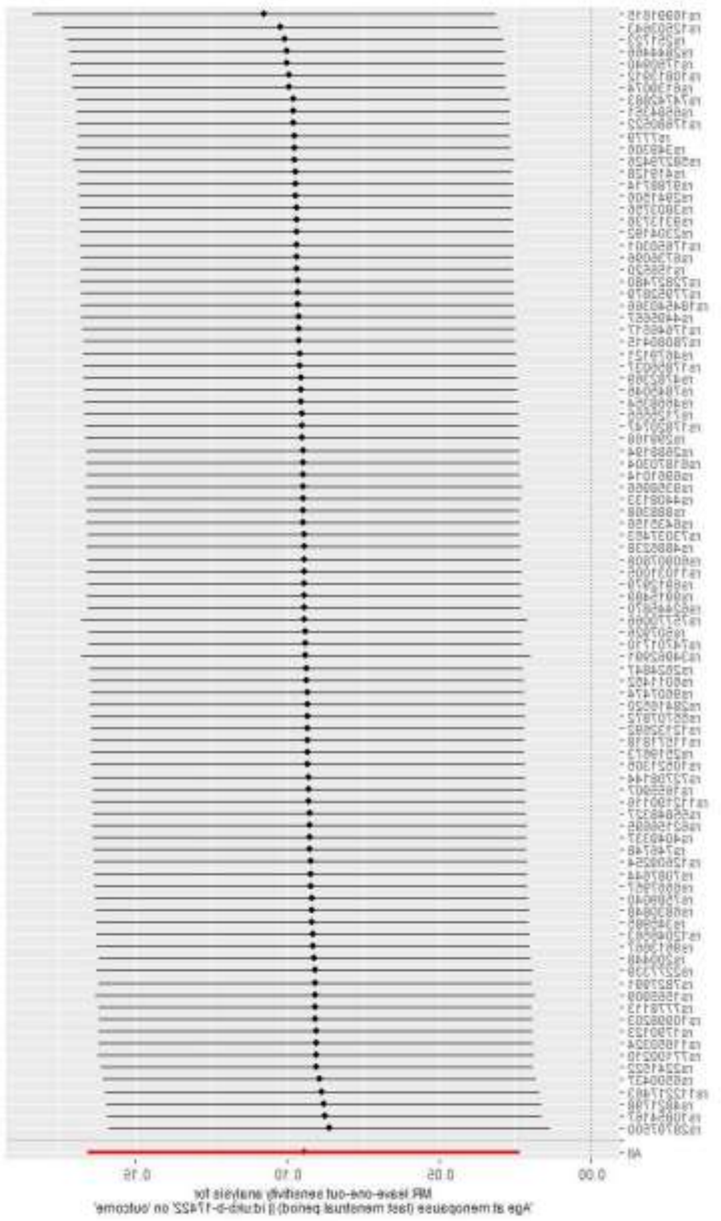


Supplementary figure 4 g

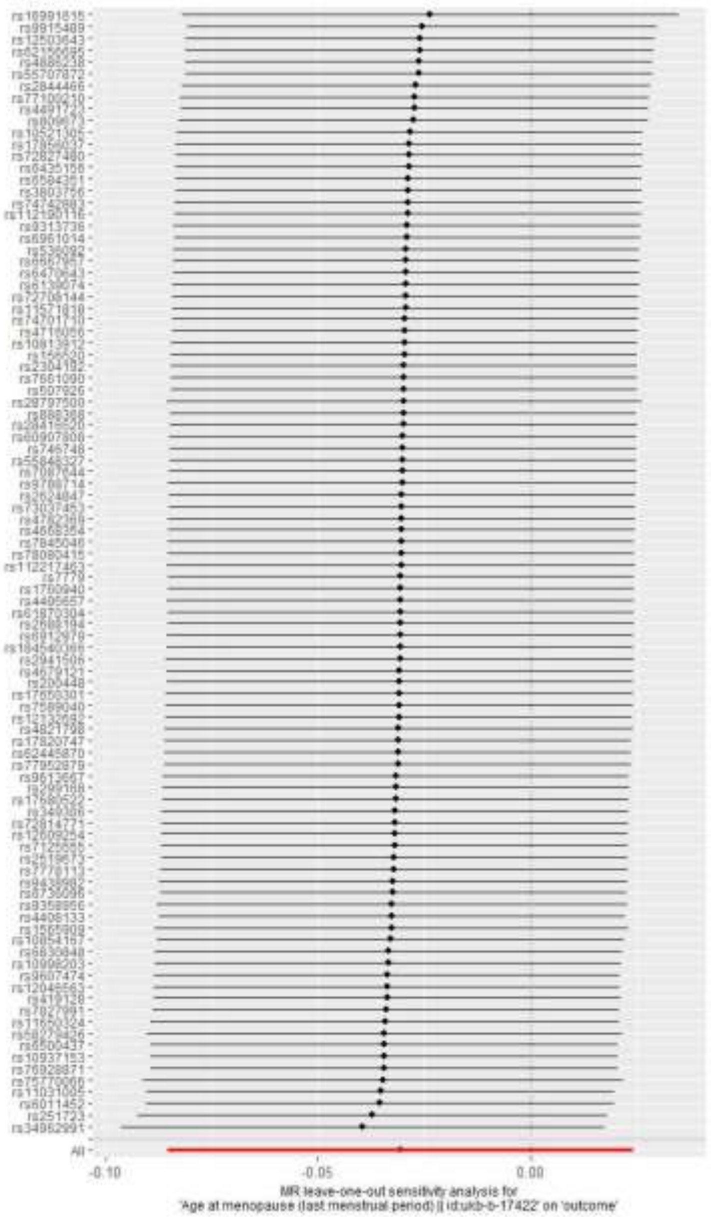


Supplementary figure 4 h

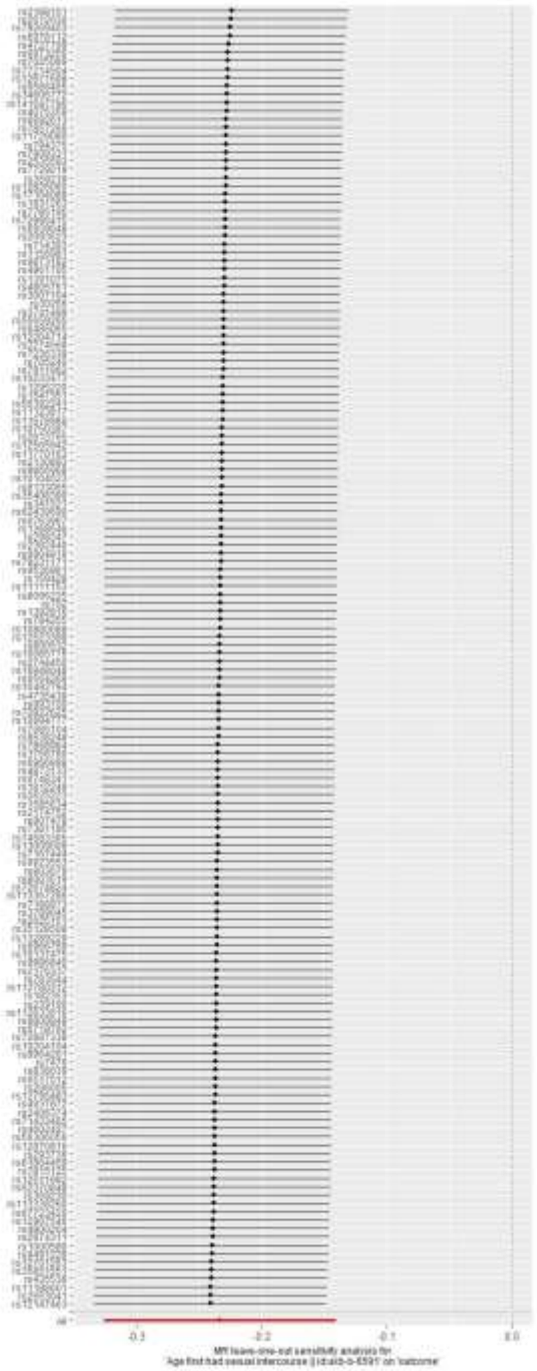


Supplementary figure 4 i

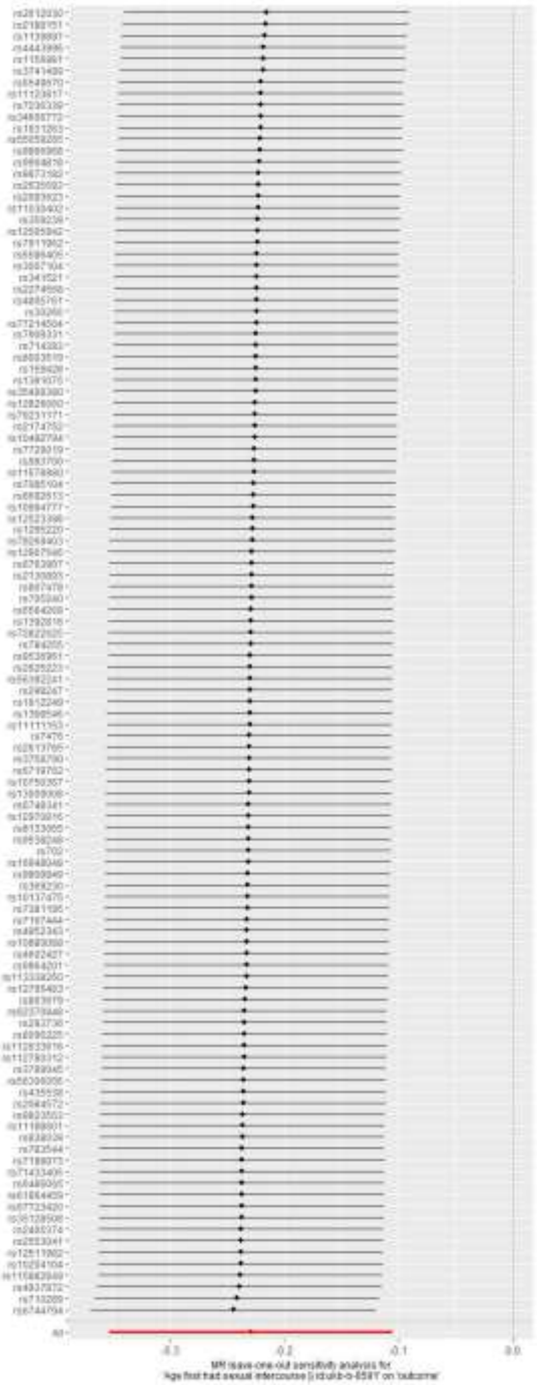


Supplementary figure 4 j

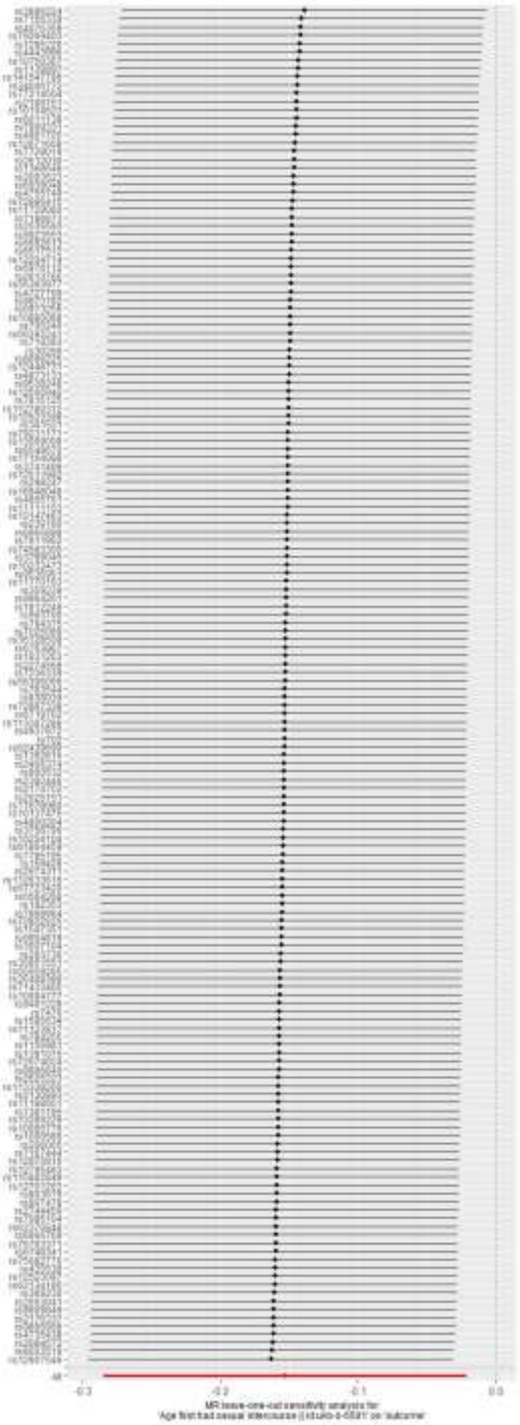


Supplementary figure k

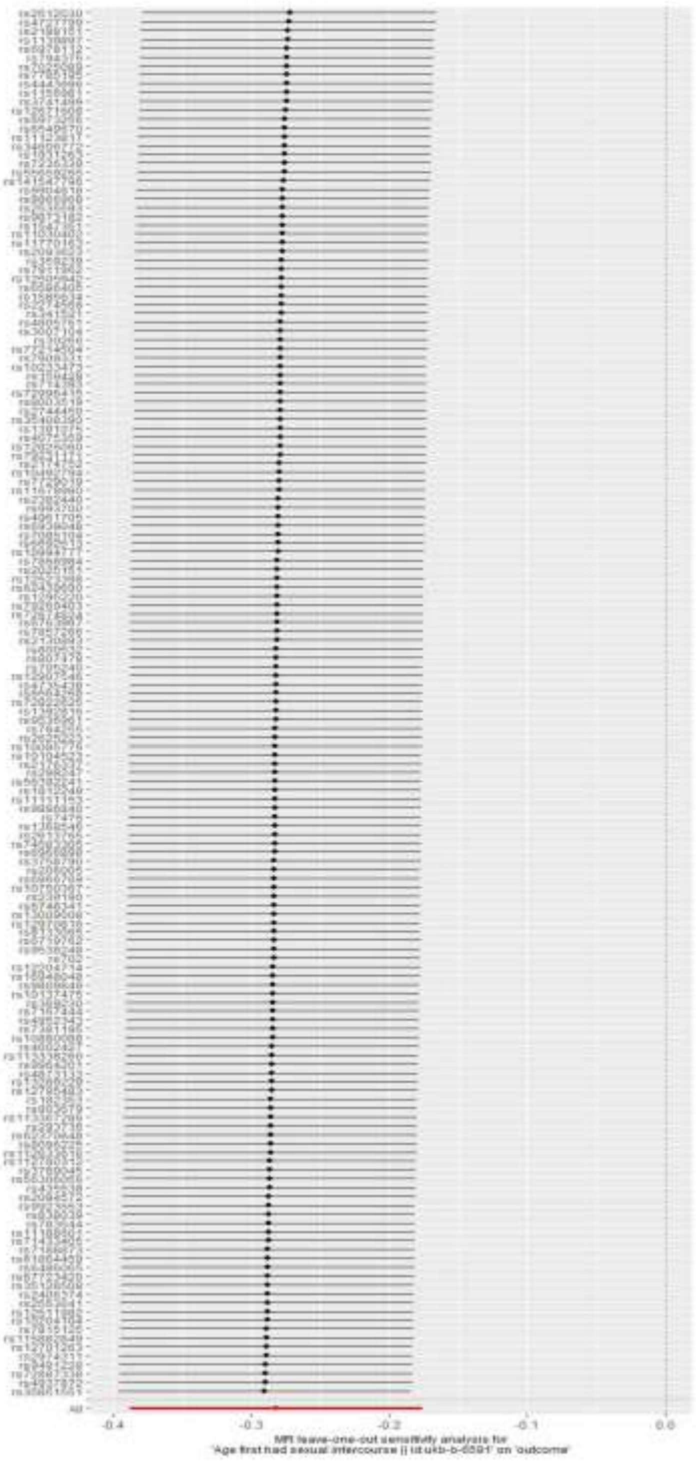

Supplementary figure l

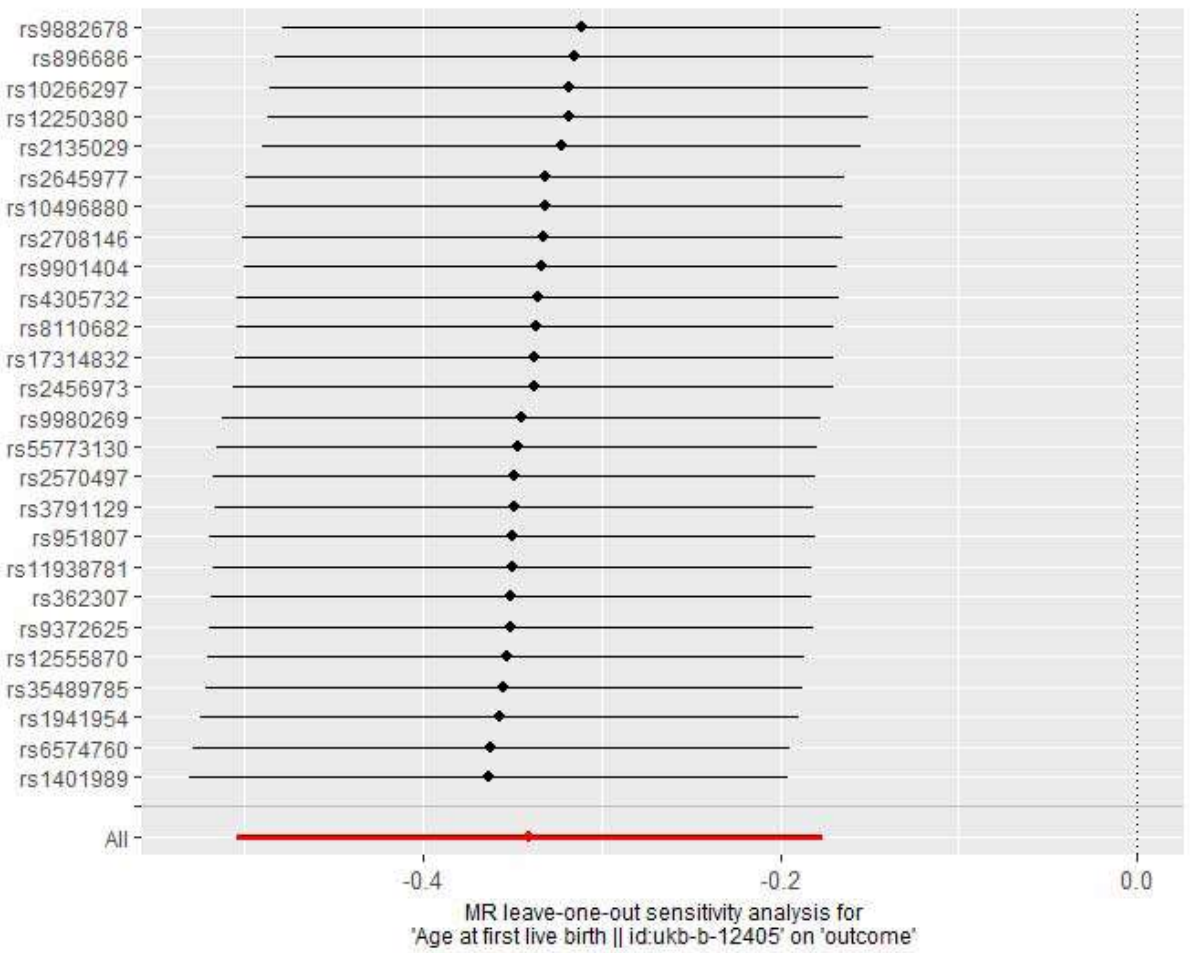


Supplementary figure 4 m

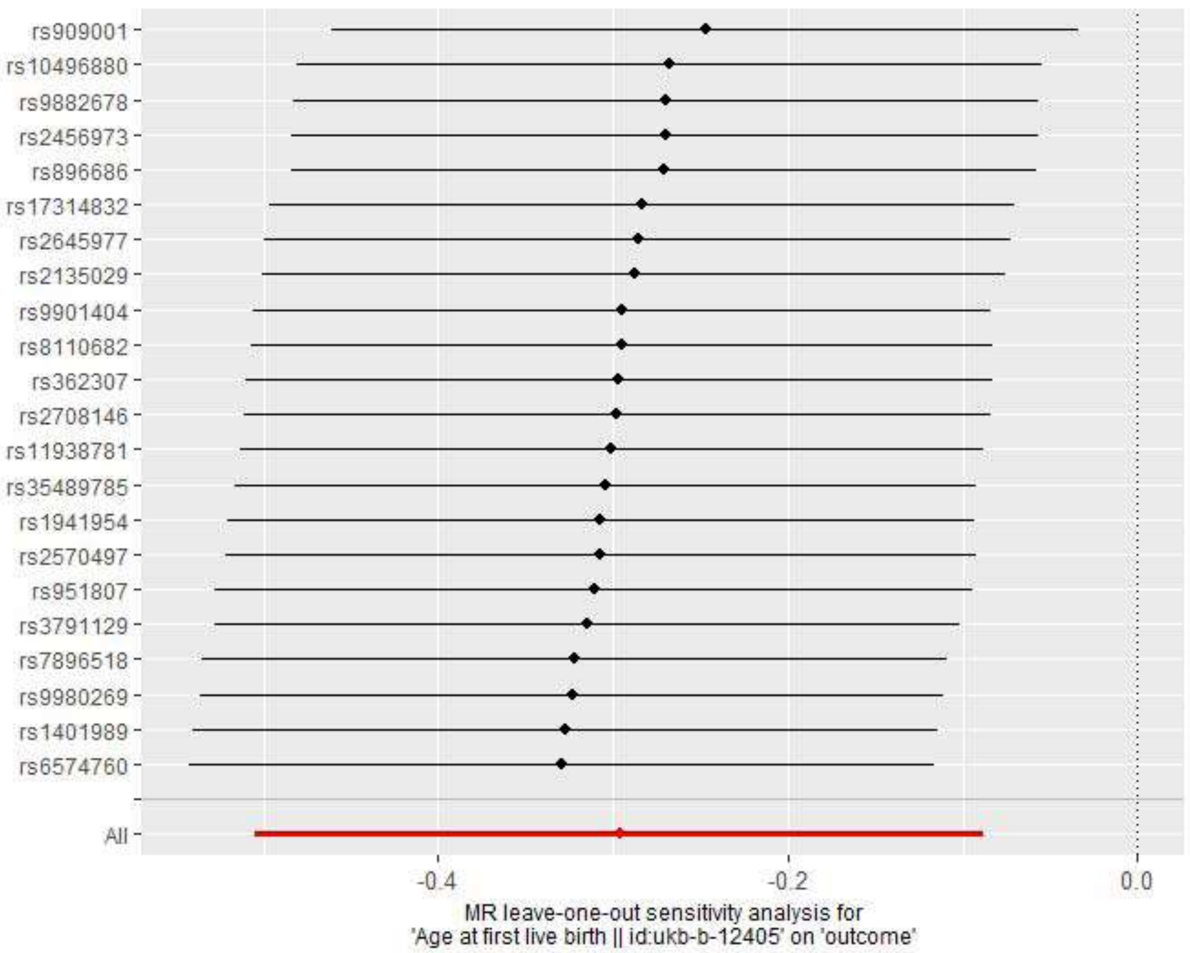


Supplementary figure 4 n

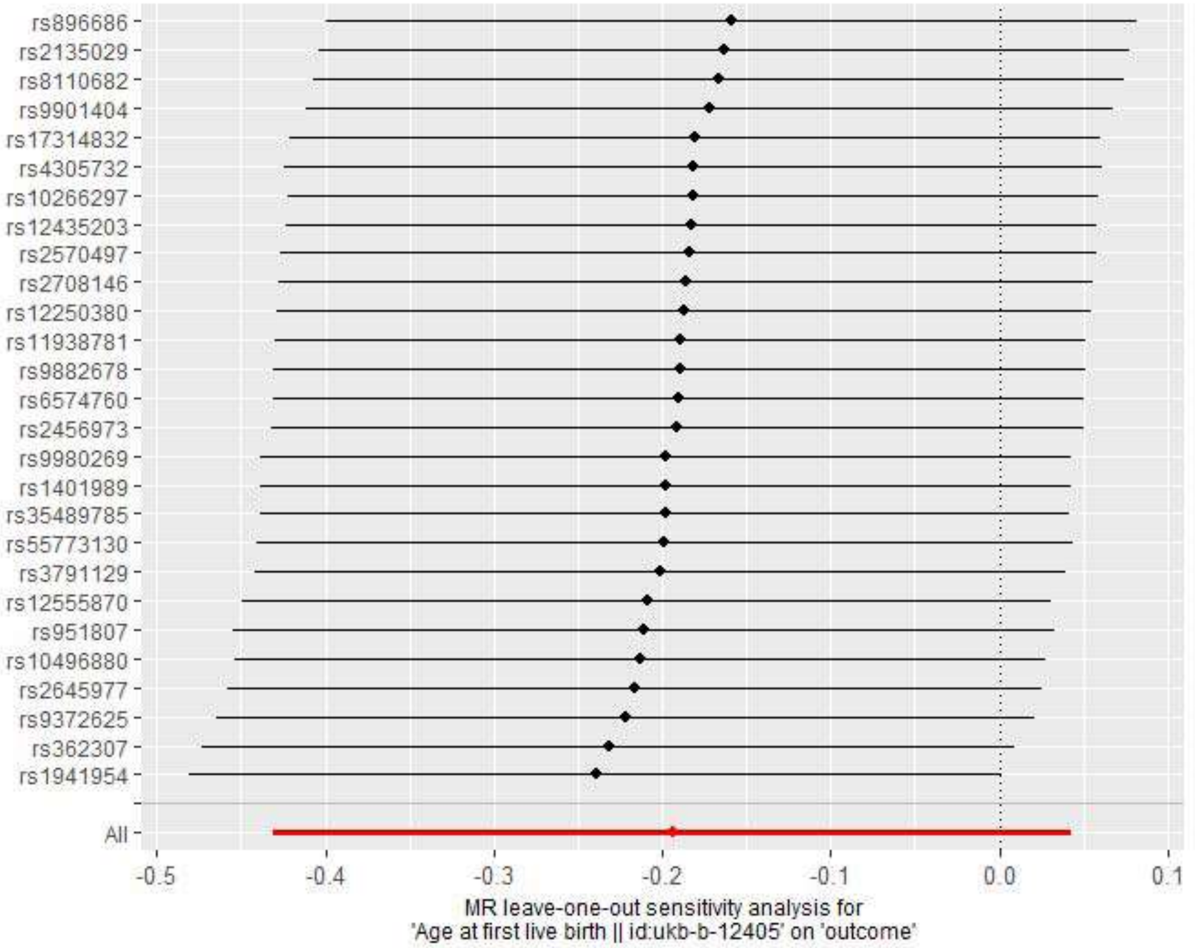


Supplementary figure 4 o

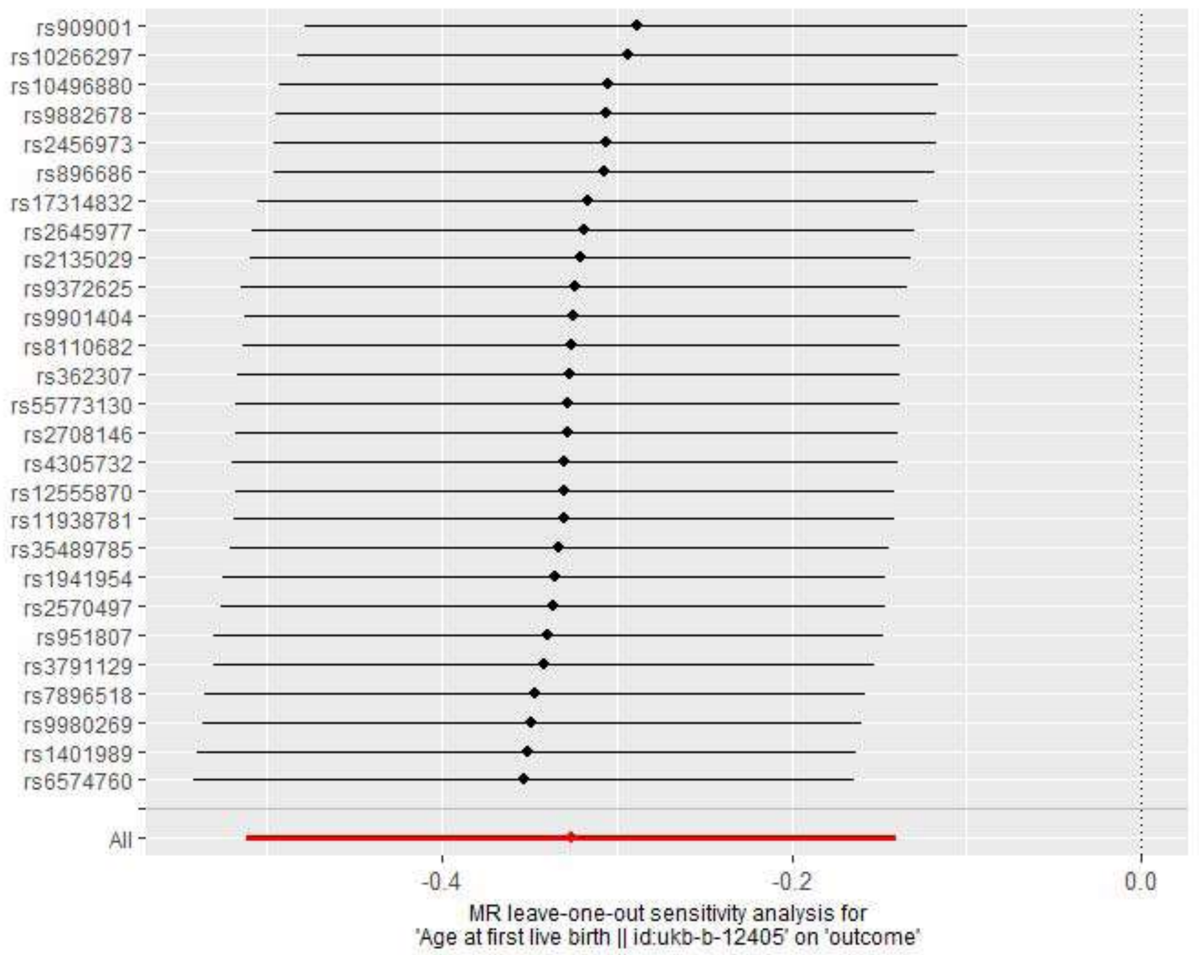


Supplementary figure 4 p

Supplementary figure 4 q

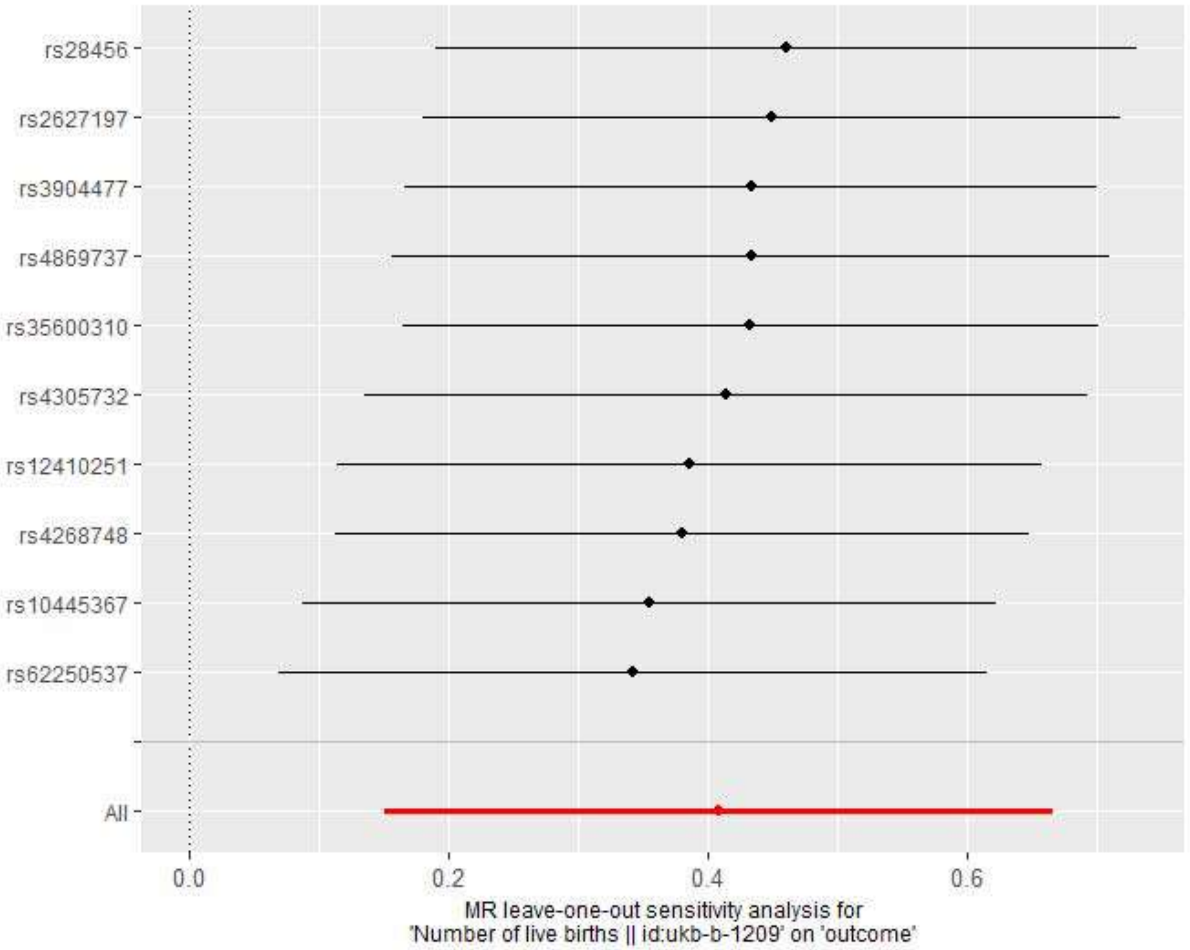

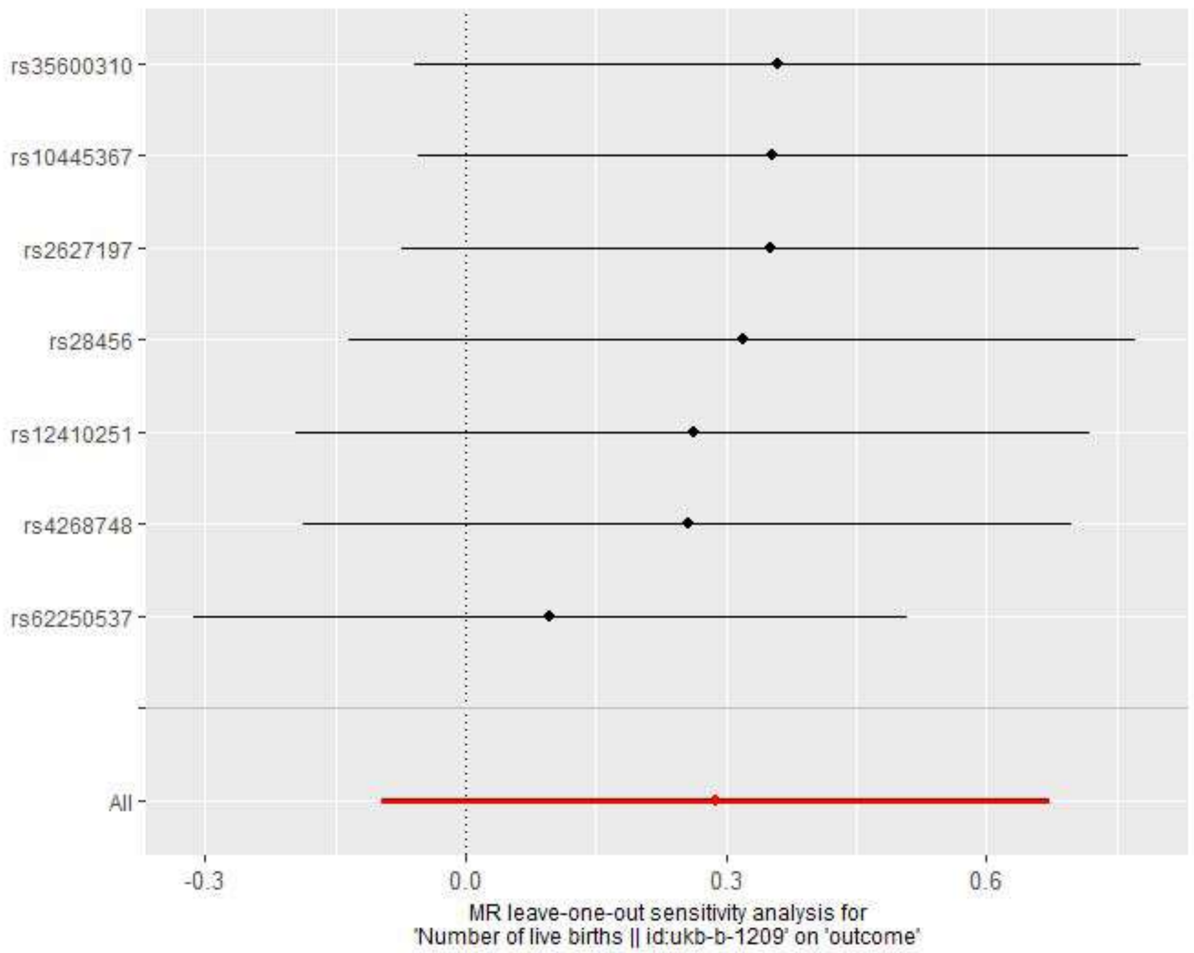


Supplementary figure 4 r

Supplementary figure 4 s

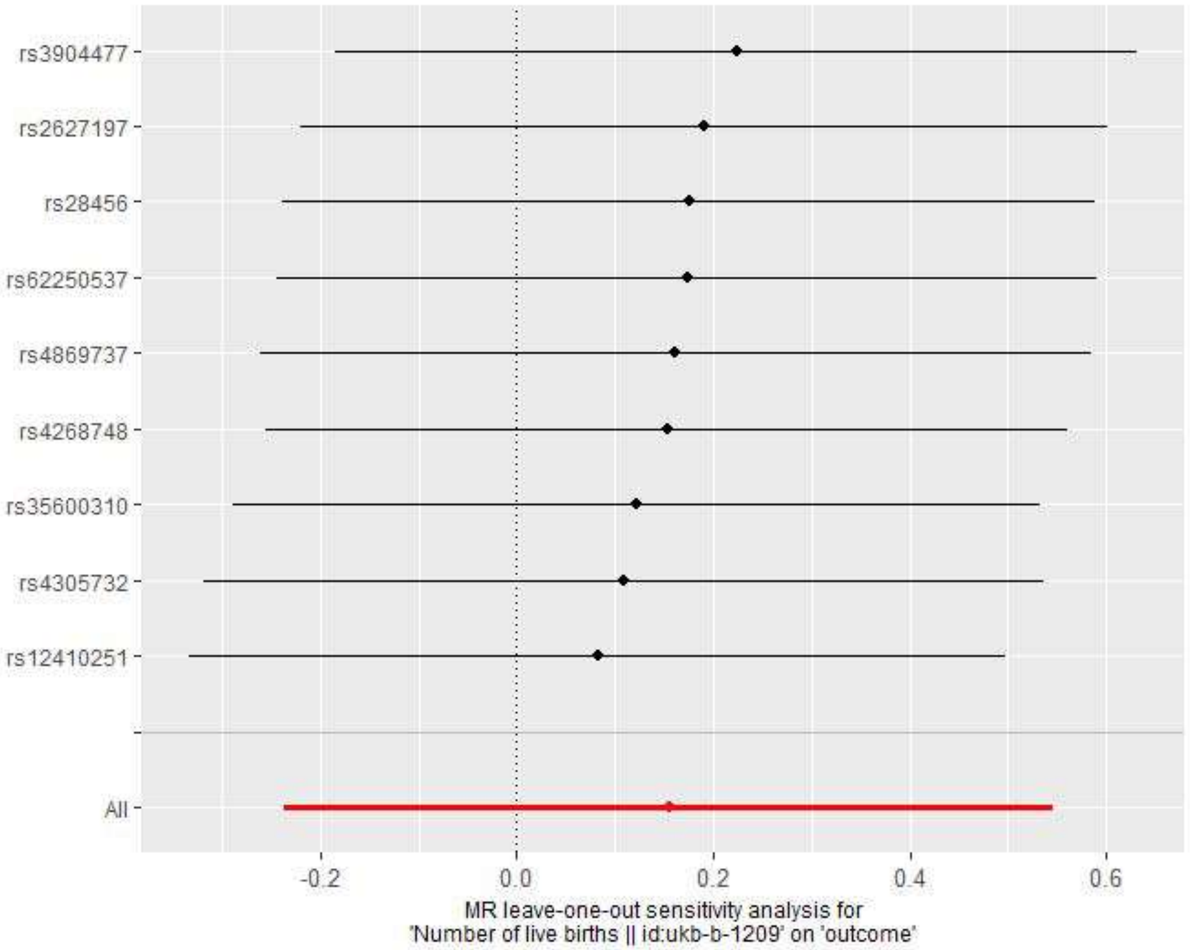

Supplementary figure 4 t

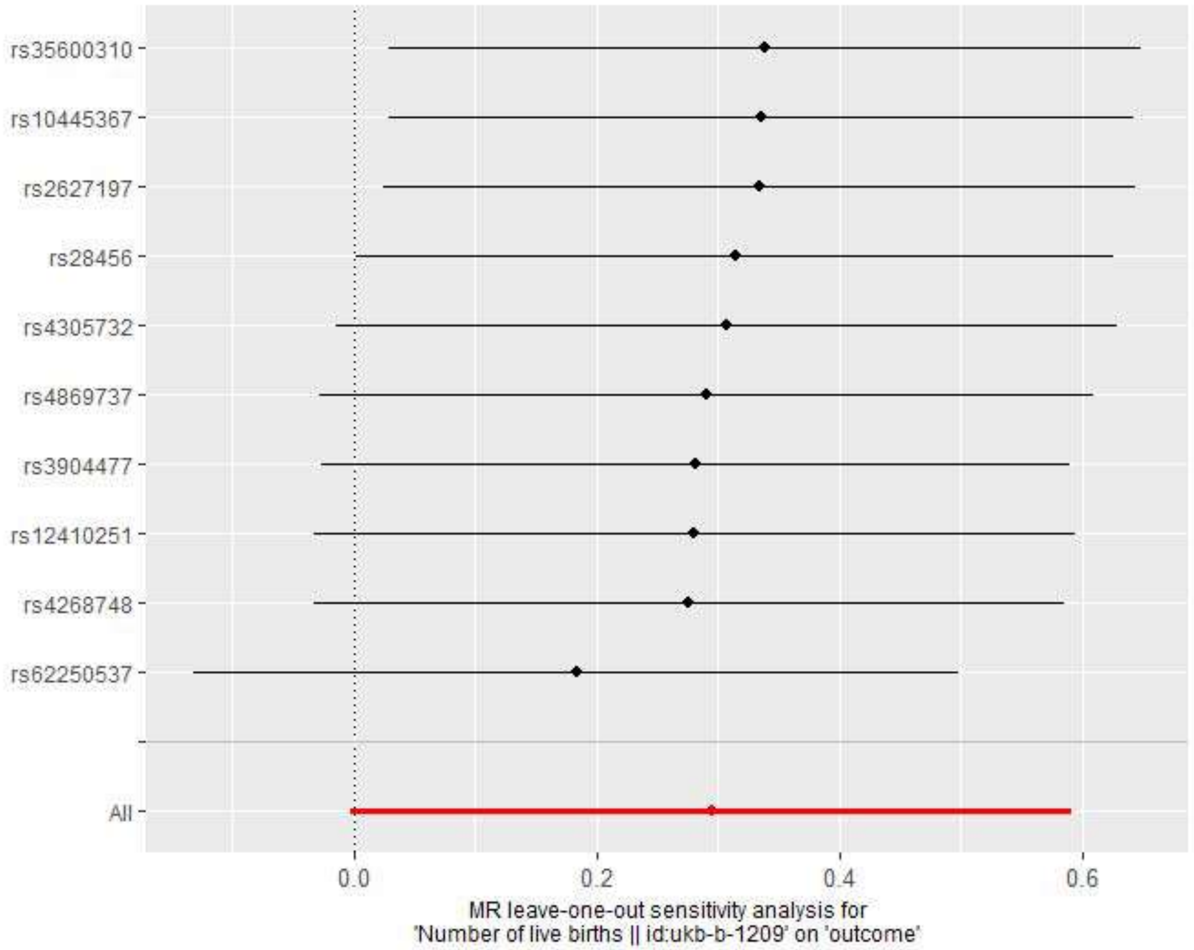

Supplement: Supplementary file 2 [file medi-104-e41362-s002.pdf]
